# Supplementary material for: Molecular folding governs switchable singlet oxygen photoproduction in porphyrin-decorated bistable rotaxanes
Source: Commun Chem. 2024 Aug 7;7:171. doi: 10.1038/s42004-024-01247-7 (PMC11306352; doi:10.1038/s42004-024-01247-7)
Supplement: Supplementary file 2 — Supporting Information [file 42004_2024_1247_MOESM2_ESM.pdf]

Supporting Information

Molecular folding governs switchable singlet oxygen photoproduction in porphyrin-decorated bistable rotaxanes

Jan Riebe[1], Benedikt Bädorf[2], Sarah Löffelsender[2], Matias Ezequiel Gutierrez Suburu[3], María Belén Rivas Aiello[3], Cristian Alejandro Strassert[3], Stefan Grimme[2]*, Jochen Niemeyer[1]*

[1] Faculty of Chemistry (Organic Chemistry) and Center for Nanointegration Duisburg-Essen (CENIDE), University of Duisburg-Essen,Universitätsstrasse 7, 45141 Essen, Germany. email: jochen.niemeyer@uni-due.de

[2] Mulliken Center for Theoretical Chemistry, Rheinische Friedrich-Wilhelms-Universität Bonn, Beringstrasse 4, 53115 Bonn, Germany. email: grimme@thch.uni-bonn.de

[3] Institut für Anorganische und Analytische Chemie, CeNTech, CiMIC, SoN, Universität Münster, Heisenbergstr. 11, 48149 Münster, Germany.

Table of Contents

1 Supplementary methods: Synthesis and characterization 1

1.1 General information 1

1.1.1 Chemicals and materials 1

1.1.2 Analytical methods 2

1.2 Syntheses 2

1.2.1 Syntheses of known compounds and precursors 2

1.2.2 Synthesis of new compounds 4

1.3 Pseudorotaxanes between ammonium half-threads and crown-ether 33

1.4 Photophysical characterization and measurements of singlet oxygen production 35

1.4.1 Measurements of singlet oxygen production with DPBF 35

1.4.2 Förster radius of the ZnTPP/BHQ-2-pair 37

1.4.3 Photophysical characterization and singlet oxygen phosphorescence 38

2 Supplementary methods: Theoretical calculations 49

2.1 Computational details 49

2.1.1 Generation of structures 49

2.1.2 Computation of Gibbs free energies 50

2.2 Results and discussion 51

2.2.1 **H-1a**$^{2+}$ and **1a**$^{+}$: Conformer searches 51

2.2.2 **H-1a**$^{2+}$ and **1a**$^{+}$: Gibbs free energies 52

2.2.3 **H-1b**$^{2+}$ and **1b**$^{+}$: Conformer searches 57

2.2.4 **H-1b**$^{2+}$ and **1b**$^{+}$: Gibbs free energies 57

2.3 Förster radius 60

3 NMR-spectra of new compounds 64

4 Supplementary References 84

1 Supplementary methods: Synthesis and characterization

1.1 General information

1.1.1 Chemicals and materials

For thin layer chromatography (TLC) analysis, Polygram® SIL G/UV254 TLC plates (silica gel 0.2 mm, 40 × 80 mm) were used. Visualization of the spots was carried out under a 254/366 nm UV light source. Flash column chromatography was performed using silica gel 60M (40-63 µm) from MACHEREY-NAGEL GmbH & Co. KG. Automated flash column chromatography was performed on a Teledyne Isco NextGen 300+ instrument with different sized silica columns by Teledyne Isco or MACHEREY-NAGEL GmbH & Co. KG. Detection wavelengths were 254, 420 or 560 nm. Dry dichloromethane was distilled from CaH_2 and stored under argon, dry tetrahydrofuran was distilled from sodium/benzophenone and stored under argon, dry DMF was distilled from CaH_2 under reduced pressure and stored over molecular sieves under argon. Acetonitrile was dried over molecular sieves under argon. Aqueous work-ups and column chromatographies were carried out using technical grade solvent. Technical grade ethyl acetate and cyclohexane were distilled before use.

Ammonium hexafluorophosphate and cesium carbonate were purchased from Carbolution.

Methyl iodide, HCl in 1,4-dioxane (4 M), potassium hexafluorophosphate, anthracene-9-carbaldehyde, thionyl chloride, diphenylisobenzofuran, sodium borohydride and lithium aluminium hydride in tetrahydrofuran (2 M) were purchased from Acros Organics.

Dibenzo-24-crown-8, tetrakis(acetonitrile)copper(I) hexafluorophosphate, Fast Black K-salt (CAS-Nr.: 64071-86-9), 2-*N*-metyhlanilinoethanol and hydroxylamine hydrochloride were purchased from TCI.

Trifluoroacetic acid, 10-undecyn-1-ol, 4-(dimethylamino)-pyridine, 3,5-di-*tert*-butylbenzyl bromide and 3,5-di-*tert*-butylbenzaldehyde were purchased from Fluorochem.

4-Toluenesulfonyl chloride, 4-pentyn-1-ol, 4-hydroxybenzylamine, 4-hydroxybenzaldehyde, catechol, tin-(II)-chloride dihydrate, (2-chlorethoxy)ethoxy)ethanol, ethyl-3,4-dihydroxy benzoate, 3-(ethylendiamino)-propyl functionalized silica gel, sodium azide and hexafluorophosphoric acid were purchased from Sigma-Aldrich.

Dimethylformamide and hydrochloric acid were purchased from Carl Roth. Triethylamine, sodium hydroxide, sodium nitrite, zinc acetate-dihydrate, methanol, acetone, dichloromethane, toluene and diethyl ether were purchased from Fischer Scientific. $CDCl_3$ and DMSO-d_6 were purchased from Deutero, CD_2Cl_2 was purchased from Eurisotop. All commercially available chemicals were used without further purification unless stated otherwise.

meso-Tetraphenylporphyrin[1] (**S7**) was synthesized according to literature methods. For the synthesis of other previously described intermediates see figures S1, S2 and S3.

1.1.2 Analytical methods

IR spectra were measured on a Jasco FT/IR-4600 spectrometer. The NMR spectra were recorded with a Bruker Avance NEO 400 spectrometer [^{1}H: 400 MHz, ^{19}F: 376 MHz, ^{31}P: 162 MHz, ^{13}C: 101 MHz] or a Bruker DRX 600 spectrometer [^{1}H: 600 MHz, ^{13}C: 151 MHz]. All measurements were performed at room temperature, using [D_1]-chloroform ($CDCl_3$), [D_2]-methylene chloride (CD_2Cl_2), [D_6]-dimethylsulfoxide (DMSO-d_6) or [D_8]-tetrahydrofurane (THF-d_8) as the solvent. The chemical shifts are referenced relative to the residual proton signals of the solvent in the ^{1}H-NMR ($CHCl_3$: δ = 7.26 ppm; CD_2Cl_2: δ = 5.32 ppm; DMSO-d_6: δ = 2.50 ppm; THF-d_8: δ = 1.73/3.58 ppm) or relative to the solvent signal in the ^{13}C-NMR ($CDCl_3$: δ = 77.16 ppm; CD_2Cl_2: δ = 53.8 ppm; DMSO-d_6: δ = 39.52 ppm). The coupling constants are given in Hertz. The description of the fine structure means: s = singlet, br s = broad singlet, d = doublet, t = triplet, quint = quintet, hept = heptet, sept = septet, m = multiplet. High resolution ESI mass spectra were recorded on a Bruker Maxis 4G spectrometer at the Institute of Organic Chemistry at the University of Duisburg-Essen. UV/Vis absorption spectra were recorded on a Varian Cary 300 Bio UV-Vis spectrophotometer in spectrophotometric grade tetrahydrofuran. Fluorescence spectra were recorded on a Varian Eclipse fluorescence spectrophotometer. For descriptions of the instruments used in measuring singlet lifetimes and singlet oxygen phosphorescence see chapter 1.4.3 for more details.

1.2 Syntheses

1.2.1 Syntheses of known compounds and precursors

The following compounds were prepared according to literature methods.

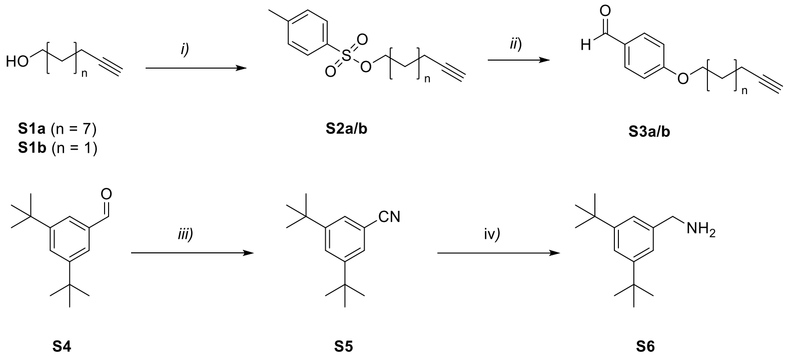

Figure S1: Synthesis of precursors for ammonium half-threads **4a/b-HPF₆**. Reagents and conditions: *i*) *p*-Toluenesulfonyl chloride, NEt_3, DMAP, DCM, 20 °C, 95%/89%[2, 3], *ii*) 4-hydroxybenzaldehyde, cesium carbonate, DMF, 85 °C, 92%/93%[4, 5], *iii*) hydroxylamine hydrochloride, EtOH, 78 °C, 94%[6, 7], *iv*) $LiAlH_4$, THF, 0 °C to 20 °C, 94%[8].

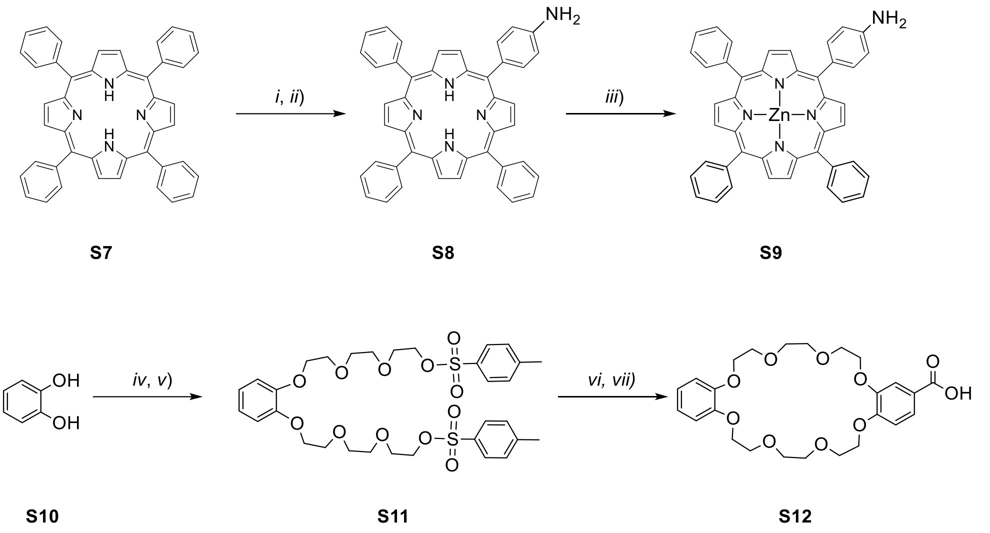

Figure S2: Synthesis of precursors for porphyrin decorated crown-ether **5**. Reagents and conditions: *i*) $NaNO_2$, glacial acetic acid, TFA, then *ii*) $SnCl_2$, conc. HCl (aq.), 100 °C, 56% over two steps[9, 10], *iii*) $Zn(OAc)_2$, $CHCl_3$/MeOH, 70 °C, 85%[11], *iv*) (2-chlorethoxy)ethoxy)ethanol, K_2CO_3, KI, DMF, 85 °C, 76%, *v*) *p*-toluenesulfonic acid, NEt_3, DMAP, DCM, 20 °C, 60%[12], *vi*) ethyl-3,4-dihydroxy benzoate, cesium carbonate, MeCN, 80 °C, 81%, *vii*) KOH, ethanol/water, 90 °C, 57%[13].

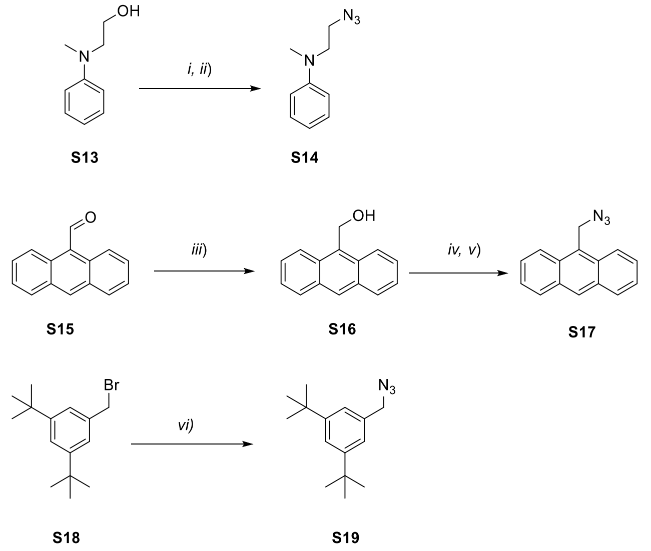

Figure S3: Synthesis of BHQ-2-precursor **S14** and stopper-azides **S17** and **S19**. Reagents and conditions: *i*) Mesyl chloride, NEt_3, DCM, 0 °C to 20 °C, then *ii*) NaN_3, MeCN, 80 °C, 81% over two steps[14], *iii*) $NaBH_4$, THF, 20 °C, 92%[15] *iv*) $SOCl_2$, DCM, then *v*) NaN_3, DMF, 60 °C, 93% over two steps[16], *vi*) NaN_3, DMF, 80 °C, 91%[17].

1.2.1.1 Synthesis of BHQ-2-azide (**6**)

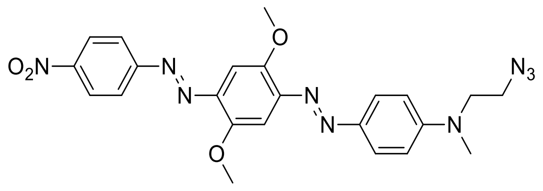

Azide **6** was prepared according to a modified literature procedure.[18]

Fast Black K-salt (CAS-Nr.: 64071-86-9) (2.75 g, 6.58 mmol, 1.2 eq.) was suspended in a mixture (10 mL, 1:1 (v:v)) of freshly prepared aqueous sodium actetate buffer (0.1 M, pH 4) and acetonitrile and sonicated for 5 minutes. This was vacuum filtered over a Por. 4 glass frit, which was rinsed with the same solvent mixture (1x5 mL). The clear brown filtrate was cooled to 0 °C in an ice bath. To this, an ice-cold solution of azide **S14** (950 mg, 5.39 mmol, 1 eq., see Figure S3) in acetonitrile (2 mL) was added in one portion with strong stirring. The color of the mixture turned dark purple after one minute, upon which the ice bath was removed and the reaction mixture was stirred at room temperature for 2 hours. The reaction mixture was vacuum filtered over a Por. 4 glass frit and the obtained solid was washed with a mixture of acetonitrile and water (2x5 mL, 1:1 (v:v)). The obtained solid was suspended in water and dried by lyophilization to yield the desired product as a very dark purple powder (294 mg, 601 µmol, 11.1% yield).

^{1}H-NMR (400 MHz, $CDCl_3$, 298 K): δ = 8.38 (d, *J* = 8.9 Hz, 2H, $H_{Nitroaryl}$), 8.05 (d, *J* = 8.9 Hz, 2H, $H_{Nitroaryl}$), 7.97 (d, *J* = 9.0 Hz, 2H, $H_{Aminoaryl}$), 7.50 (s, 1H, H_{Aryl}), 7.46 (s, 1H, H_{Aryl}), 6.82 (d, *J* = 9.0 Hz, 2H, $H_{Aminoaryl}$), 4.10 (s, 3H, OCH_3), 4.05 (s, 3H, OCH_3), 3.68 (t, *J* = 5.9 Hz, 2H, CH_2), 3.55 (t, *J* = 5.8 Hz, 2H, CH_2), 3.18 (s, 3H, NCH_3).

1.2.2 Synthesis of new compounds

1.2.2.1 Synthesis of dibenzylammonium salt **4a-HPF$_6$**

1.2.2.1.1 Synthesis of dibenzylamine **4a**

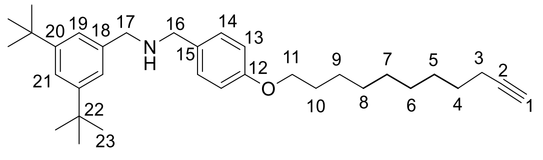

3,5-Di-*tert*-butylbenzylamine (**S6)** (24.2 mg, 110 µmol, 1 eq.) and alkylated benzaldehyde **S3a** (30.0 mg, 110 µmol, 1 eq.) were dissolved in methanol (1 mL) under argon atmosphere and heated to reflux for 18 hours. The resulting solution was cooled to 0 °C and sodium borohydride (41.7 mg, 1.26 mmol, 12 eq.) was added in one portion. The reaction mixture was stirred at 0 °C for 10 minutes and for another 20 hours at room temperature under argon atmosphere. After that, the reaction mixture was cooled to 0 °C and water (1 mL) was added. The resulting mixture was stirred at 0 °C for 10 minutes and then at room temperature for 10 minutes to yield a slightly cloudy solution. This was filtered through a pad of celite, which was washed with dichloromethane (3 x 10 mL). Concentration of the combined filtrates *in vacuo* led to a colorless oil, which was purified by column chromatography on silica gel (2 x 20 cm, eluent: dichloromethane/cyclohexane 95/5 + 2% triethylamine). The obtained product was a colorless oil (34.0 mg, 71.5 µmol, 65.0% yield).

$C_{33}H_{49}NO$: 475.76 g/mol

^{1}H-NMR (400 MHz, $CDCl_3$, 298 K): δ = 7.32 (t, *J* = 1.6 Hz, 1H, H-21), 7.26 (d, *J* = 8.6 Hz, 2H, H-14), 7.17 (d, *J* = 1.6 Hz, 2H, H-19), 6.86 (d, *J* = 8.6 Hz, 2H, H-13), 3.94 (t, *J* = 6.6 Hz, 2H, H-11), 3.79 (s, 2H, H-17), 3.77 (s, 2H, H-16), 2.18 (td, *J* = 7.1, 2.6 Hz, 2H, H-3), 1.94 (t, *J* = 2.7 Hz, 1H, H-1), 1.84 – 1.73 (m, 2H, H-10), 1.58 – 1.48 (m, 2H, H-4), 1.47 – 1.26 (m, 28H, H-5 to H-9 and H-23).

^{13}C{^{1}H}-NMR (101 MHz, $CDCl_3$, 298 K): δ = 158.4 (C-12), 150.9 (C-20), 129.6 (C-14), 122.5 (C-19), 121.1 (C-21), 114.5 (C-13), 84.9 (C-2), 68.2 (C-1), 68.1 (C-11), 53.7 (C-17), 52.7 (C-16), 35.0 (C-22), 31.7 (C-23), 29.5, 29.6, 29.2, 28.9, 26.2 (C-5 to C-9), 29.4 (C-10), 28.6 (C-4), 18.5 (C-3).

Full characterization was performed for the hexafluorophosphate-salt **4a-HPF$_6$**.

1.2.2.1.2 Formation of dibenzylammonium salt **4a-HPF$_6$**

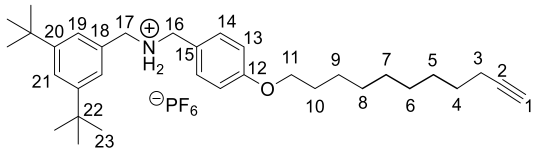

Dibenzylamine **4a** (32.5 mg, 68.3 µmol, 1 eq.) was dissolved in diethyl ether (2 mL) and hydrogen chloride (4 M in dry 1,4-dioxane, 0.30 mL, 1.20 mmol, 18 eq.) was added. The reaction mixture was stirred at room temperature for one hour. Volatiles were removed in vacuo and the remaining viscous oil was dissolved in dry dichloromethane (3 mL). After addition of solid ammonium hexafluorophosphate (560 mg, 3.44 mmol, 50 eq.), the reaction mixture was stirred at room temperature for 20 hours. Suspended solids were removed by filtration and the filtrate was concentrated in vacuo to yield the desired compound as a slightly yellow wax (34.5 mg, 55.5 µmol, 81.3% yield).

$C_{33}H_{50}NO$-PF_6: 621.73 g/mol

^{1}H-NMR (400 MHz, $CDCl_3$ 298 K): δ = 7.44 (t, *J* = 1.7 Hz, 1H, H-21), 7.26 (d, *J* = 8.7 Hz, 2H, H-14, partially overlapping with the solvent signal), 7.19 (d, *J* = 1.7 Hz, 2H, H-19), 6.86 (d, *J* = 8.7 Hz, 2H, H-13), 5.52 (br s, 2H, N*H$_2$*), 4.01 (s, 2H, H-17), 3.96 (s, 2H, H-16), 3.87 (*t*, *J* = 6.6 Hz, 2H, H-11), 2.18 (td, *J* = 7.1, 2.6 Hz, 2H, H-3), 1.93 (t, *J* = 2.6 Hz, 1H, H-1), 1.78 – 1.67 (m, 2H, H-10), 1.58 – 1.48 (m, 2H, H-4), 1.45 – 1.37 (m, 4H, H-9 and H-5), 1.36 – 1.28 (m, 24H, H-6 to H-8, H-23).

^{13}C{^{1}H}-NMR (101 MHz, $CDCl_3$, 298 K): δ = 160.2 (C-12), 152.3 (C-20), 131.4 (C-14), 129.8 (C-18), 124.0 (C-19), 123.7 (C-21), 122.3 (C-15), 115.2 (C-13), 84.9 (C-2), 68.2 (C-1), 68.1 (C-11), 51.2 (C-17), 50.2 (C-16), 35.1 (C-22), 31.4 (C-23), 29.5, 29.4, 29.3 (C-6, C-7, C-8), 29.2 (C-10), 28.8 (C-5), 28.6 (C-4), 26.1 (C-9), 18.5 (C-3).

^{19}F{^{1}H}-NMR (376 MHz, $CDCl_3$, 298 K): δ = -70.5 (d, *J* = 714.9 Hz, P*F*$_6^-$).

^{31}P{^{1}H}-NMR (162 MHz, $CDCl_3$, 298 K): δ = -144.0 (sept, *J* = 714.9 Hz, *P*F$_6^-$).

HSQC (400 MHz/101 MHz, $CDCl_3$, 298 K): δ (^{1}H) / δ (^{13}C) = 7.44/123.7 (H-21/C-21), 7.26/131.4 (H-14/C-14), 7.19/124.0 (H-19/C-19), 6.86/115.2 (H-13/C-13), 4.01/51.2 (H-17/C-17), 3.96/50.2 (H-16/C-16), 3.87/68.1 (H-11/C-11), 2.18/18.5 (H-3/C-3), 1.78 – 1.67/29.2 (H-10/C-10), 1.58 – 1.48/28.6 (H-4/C-4), 1.58 – 1.48/28.8, 26.1 (H-5/C-5 and H-9/C-9), 1.36 – 1.28/31.4, 29.5, 29.4, 29.3 (H-23/C-23 and H-6 to H-8/C-6 to C-8).

HMBC (400 MHz/101 MHz, $CDCl_3$): δ (^{1}H) / δ (^{13}C) = 7.44/123.7, 35.1 (H-21/C-21, C-22), 7.26/131.4, 50.2 (H-14/C-14, C-16), 7.19/123.7, 51.2, 35.1 (H-19/C-21, C-17, C-22), 6.86/160.2, 122.3, 115.2 (H-13/C-12, C-15, C-13), 4.01/129.8, 124.0, 50.2 (H-17/C-18, C-19, C-16), 3.96/131.4, 122.3, 51.2 (H-16/C-14, C-15, C-17), 3.87/160.2, 29.2, 26.1 (H-11/C-12, C-10, C-9), 2.18/84.9, 68.2, 28.6 (H-3/C-2, C-1, C-4), 1.78 – 1.67/68.1, 29.2, 26.1 (H-10/C-11, C-10, C-9), 1.58 – 1.48 /84.9, 28.8, 18.5 (H-4/C-2, C-5, C-3), 1.45 – 1.37/29.3 (H-9 and H-5/C-6 or C-8), 1.36 – 1.28/152.3, 35.1, 31.4, 28.8, 26.1 (H-6, H-7, H-8, H-23/C-20, C-22, C-23, C-5, C-9).

COSY (400 MHz/400 MHz, $CDCl_3$, 298 K): δ (^{1}H) / δ (^{1}H) = 7.44/7.19 (H-21/H-19), 7.26/6.86 (H-14/H-13), 7.19/7.44, 4.01 (H-19/H-21, H17), 6.86/7.26 (H-13/H-14), 4.01/7.19 (H-17/H-19), 3.87/1.78 – 1.67 (H-11/H-10), 2.18/1.93, 1.58 – 1.48 (H-3/H-1, H-4), 1.93/2.18 (H-1/H-3), 1.78 – 1.67/3.87, 1.45 – 1.37 (H-10/H-11, H-9), 1.58 – 1.48/2.18, 1.45 – 1.37 (H-4/H-3, H-5), 1.45 – 1.37/1.78 – 1.67, 1.58 – 1.48, 1.36 – 1.28 (H-5 and H-9/H-10, H-4, H-6 and H-8), 1.36 – 1.28/1.45 – 1.37 (H-6 and H-8/H-9 and H-5).

IR (ATR-FT): $\tilde{\nu}$ = 3258 (w), 3229 (w), 2952 (broad), 2863 (m), 1610 (w), 1586 (w), 1517 (w), 1462 (w), 1403 (m), 1364 (w), 1303 (w), 1249 (m), 1184 (w), 1026 (w), 929 (w), 882 (s), 810 (s), 762 (s), 711 (m), 627 (m), 555 (s) cm^{-1}.

MS (ESI-pos, MeOH): m/z = 476.3893 ($[M-PF_6]^+$, calcd. 476.3887 for $[C_{33}H_{50}NO]^+$).

1.2.2.2 Synthesis of dibenzylamonium salt with shorter alkyne **4b-HPF$_6$**

1.2.2.2.1 Synthesis of dibenzylamine **4b**

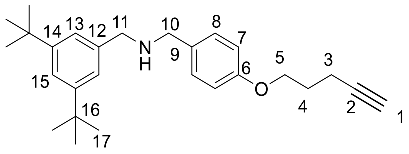

Di-*tert*-butylbenzylamine (**S6**) (100 mg, 456 µmol, 1 eq.) and alkylated benzaldehyde **S3b** (85.8 mg, 456 µmol, 1 eq.) were dissolved in methanol (2.5 mL) and heated to reflux under argon atmosphere for 2 hours. The solution was then cooled in an ice bath and sodium borohydride (173 mg, 4.56 mmol, 10 eq.) was added. The mixture was stirred at 0 °C for 5 minutes, then the ice bath was removed and the mixture was stirred at room temperature for 14 hours. Then the solution was cooled to 0 °C and water (2.5 mL) was carefully added dropwise. After complete addition, the mixture was stirred at room temperature for 10 minutes and then transferred to a separatory funnel and extracted with chloroform (3 x 10 mL). The combined organic phases were dried over sodium sulphate, filtered and concentrated *in vacuo*. The crude product was purified by column chromatography on silica gel (2 x 15 cm, eluent: cyclohexane/ethyl acetate 8/2 + 2% triethylamine). The desired amine (80.2 mg, 205 µmol, 44.9% yield) was obtained as a colorless oil.

$C_{27}H_{37}NO$: 391.5990 g/mol

^{1}H-NMR (400 MHz, $CDCl_3$, 298 K) δ = 7.36 (ps t, *J* = 1.8 Hz, 1H, H-15), 7.30 (d, *J* = 8.6 Hz, 2H, H-8), 7.21 (d, *J* = 1.8 Hz, 2H, H-13), 6.92 (d, *J* = 8.6 Hz, 2H, H-7), 4.10 (t, *J* = 6.1 Hz, 2H, H-5), 3.83 (s, 2H, H-11), 3.81 (s, 2H, H-10), 2.44 (dt, J = 7.0, 2.7 Hz, 2H, H-3), 2.08 – 1.98 (m, 3H, H-4 and H-1), 1.37 (s, 18H, H-17).

$^{13}C\{^1H\}$-NMR (101 MHz, $CDCl_3$, 298 K) δ = 158.0 (C-6), 150.9 (C-14), 139.4 (C-12), 132.7 (C-9), 129.5 (C-8), 122.5 (C-13), 121.1 (C-15), 114.5 (C-7), 83.6 (C-2), 69.0 (C-1), 66.3 (C-5), 53.8 (C-11), 52.8 (C-10), 34.9 (C-16), 31.6 (C-17), 28.3 (C-4), 15.3 (C-3).

Full characterization was performed for the hexafluorophosphate-salt **4b-HPF_6**.

1.2.2.2.2 Synthesis of dibenzylammonium salt **4b-HPF_6**

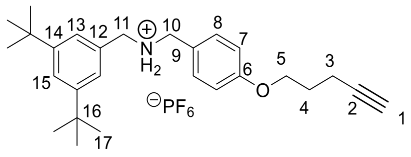

The amine **4b** (67.5 mg, 172 µmol, 1 eq.) was dissolved in methanol (2 mL) and aqueos hexafluorophosphoric acid (70%, 25 µL, 1.1 eq.) was added. The mixture was diluted with water (5 mL) and extracted with ethyl acetate (3 x 15 mL). The organic phases were dried over sodium sulphate, filtered and dried *in vacuo*, yielding the desired ammonium salt as a yellow solidified oil (92.0 mg, 171 µmol, 98.2% yield).

$C_{27}H_{38}NO$-PF_6 : 537.5712 g/mol

^1H-NMR (400 MHz, $CDCl_3$, 298 K) δ = 8.68 (br s, 2H, N*H*$_2$), 7.41 (ps t, *J* = 1.9 Hz, 1H, H-15), 7.29 – 7.23 (m, overlapping with solvent signal, 2H, H-8), 7.20 (d, *J* = 1.9 Hz, 2H, H-13), 6.83 (d, *J* = 8.2 Hz, 2H, H-7), 4.03 – 3.88 (m, 6H, H-11, H-10, H-5), 2.35 (dt, *J* = 7.0, 2.6, 2H, H-3), 2.00 – 1.88 (m, 3H, H-1 and H-4), 1.30 (s, 18H, H-17).

$^{13}C\{^1H\}$-NMR (101 MHz, $CDCl_3$, 298 K) δ = 159.8 (C-6), 152.1 (C-14), 131.7 (C-8), 129.3 (C-12), 124.3 (C-13), 123.5 (C-15), 122.1 (C-9), 115.1 (C-7), 83.5 (C-2), 69.1 (C-1), 66.2 (C-5), 49.7 (C-11), 48.6 (C-10), 35.1 (C-16), 31.5 (C-17), 28.2 (C-4), 15.3 (C-3).

HSQC (400 MHz / 101 MHz, $CDCl_3$, 298 K) δ (^{1}H) / δ (^{13}C) = 7.41/123.5 (H-15/C-15), 7.29 – 7.23/131.7 (H-8/C-8), 7.20/124.3 (H-13/C-13), 6.83/115.1 (H-7/C-7), 4.03 – 3.88/66.2, 49.7, 48.6 (H-5/C-5 and H-11/C-11 and H-10/C-10), 2.35/15.3 (H-3/C-3), 2.00 – 1.88/69.1, 28.2 (H-1/C-1 and H-4/C-4), 1.30/31.5 (H-17/C-17).

HMBC (400 MHz / 101 MHz, $CDCl_3$, 298 K) δ (^{1}H) / δ (^{13}C) = 7.41/124.3, 35.1 (H-15/C-13, C-16), 7.29 – 7.23/159.8 (H-8/C-6), 7.20/123.5, 49.7 (H-13/C-15, C-11), 6.83/159.8, 122.1, 115.1 (H-7/C-6, C-9, C-7), 4.03 – 3.88/28.2, 15.3 (H-5/C-4, C-3), 2.35/83.5, 28.2 (H-3/C-2, C-4), 2.00 – 1.88/83.5, 66.2, 15.3 (H-1/C-2, H-4/C-5, C-3), 1.30/152.1, 35.1 (H-17/C-14, C-16).

COSY (400 MHz / 400 MHz, $CDCl_3$, 298 K) δ (^{1}H) / δ (^{1}H) = 8.68/4.03 – 3.88 (N*H*$_2$/H-11, H-10), 7.41/7.20 (H-15/H-13), 7.29 – 7.23/6.83 (H-8/H-7), 7.20/7.41 (H-13/H-15), 6.83/7.29 – 7.23 (H-7/H-8), 4.03 – 3.88/8.68, 2.00 – 1.88 (H-11 and H-10/N*H*$_2$, H-5/H4), 2.35/2.00 – 1.88 (H-3/H-1, H-4), 2.00 – 1.88/4.03 – 3.88, 2.35 (H-4/H-5, H-1 and H-4/H-3).

$^{19}F\{^1H\}$-NMR (376 MHz, $CDCl_3$, 298 K): δ = -70.7 (d, *J* = 714.9 Hz, P*F*$_6^-$).

$^{31}P\{^1H\}$-NMR (162 MHz, $CDCl_3$, 298 K): δ = -144.0 (sept, *J* = 714.9 Hz, *P*F$_6^-$).

IR (ATR-FT): $\tilde{\nu}$ = 3299 (w), 2957 (m), 2904 (w), 2871 (w), 1607 (m), 1515 (m), 1463 (w), 1432 (w), 1364 (w), 1300 (w), 1278 (s), 1042 (m), 986 (m), 872 (w), 828 (s), 740 (m), 711 (s), 633 (m), 493 (s) cm^{-1}.

MS (ESI-pos, MeOH): m/z = 392.2950 ([M-PF$_6$]$^+$, calcd. 392.2948 for [$C_{27}H_{38}NO$]$^+$).

1.2.2.3 Synthesis of porphyrin decorated macroclyce **5**

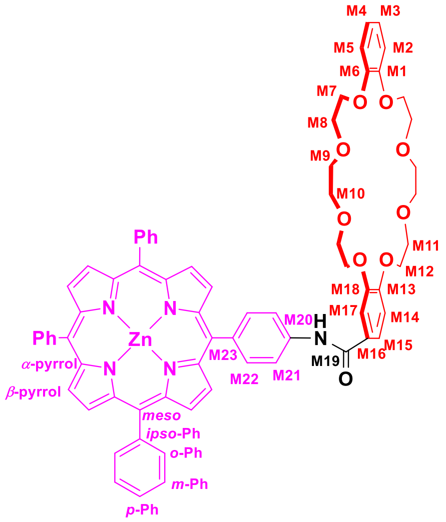

The functionalized crown ether **S12** (100 mg, 203 µmol, 1 eq.) was suspended in chloroform (1.5 mL) under argon atmosphere. Thionyl chloride (162 µL, 223 µmol, 11 eq.) was added and the mixture was refluxed for one hour. Volatiles were removed *in vacuo* to yield the acyl chloride as a slightly yellow waxy solid. This was dissolved in dry tetrahydrofuran (7 mL) and added to a solution of ZnTPP-NH$_2$ (**S9**) (139 mg, 201 µmol, 0.99 eq.) and triethylamine (45.0 µL, 325 µmol, 1.6 eq.) in dry tetrahydrofuran (20 mL) under argon *via* syringe. The reaction mixture was stirred under argon at room temperature for 22 hours. Then volatiles were removed *in vacuo* and the crude product was purified by flash column chromatography on a Teledyne Isco NextGen 300+ using a 12g "Redisep" silica column with a dichloromethane/methanol gradient (see Figure S4). The desired product was obtained as a purple powder (215 mg, 184 µmol, 90.7% yield).

$C_{69}H_{59}N_5O_9Zn$: 1167.6370 g/mol

^{1}H-NMR (400 MHz, DMSO-d$_6$, 298 K): δ = 10.50 (s, 1H, amide-N*H*), 8.89 (d, *J* = 4.6 Hz, 2H, H$_{\beta\text{-pyrrol}}$), 8.82 – 8.76 (m, 6H, H$_{\beta\text{-pyrrol'}}$), 8.28 – 8.13 (m, 10H, H$_{o\text{-Ph}}$ and H-M22 and H-M21), 7.85 – 7.70 (m, 11H, H$_{m\text{-Ph}}$ and H$_{p\text{-Ph}}$ and H-M15 and H-M17), 7.16 (d, *J* = 8.5 Hz, 2H, H-M14), 6.97 – 6.84 (m, 4H, H-M2, H-M3, H-M4 and H-M5), 4.27 – 4.23 (m, 2H, H-M12), 4.21 – 4.16 (m, 2H, H-M12'), 4.08 – 4.02 (m, 4H, H-M7), 3.87 – 3.82 (m, 2H, H-M11), 3.82 – 3.78 (m, 2H, H-M11'), 3.77 – 3.72 (m, 4H, H-M8), 3.72 – 3.61 (m, 8H, H-M9 and H-M-10).

^{13}C{^{1}H}-NMR (101 MHz, DMSO-d$_6$, 298 K): δ = 165.3 (C-M19), 151.4 (C-M13), 149.5 (C$_{\alpha\text{-pyrrole}}$), 149.4 – 149.2 (overlapping signals, C$_{\alpha\text{-pyrrole}}$), 148.6 – 148.4 (overlapping signals, C-M1 and C-M6), 147.9 (C-M18), 142.8 (C$_{ipso\text{-}Ph}$), 138.8 (C-M20), 137.8 (C-M23), 134.5 (C-M22), 134.2 (C$_{o\text{-Ph}}$), 131.9 – 131.4 (overlapping signals, C$_{\beta\text{-pyrrole}}$), 127.6 – 127.4 (overlapping signals, C$_{p\text{-Ph}}$ and C-M16), 126.6 (C$_{m\text{-Ph}}$), 121.6 (C-M15), 121.4 – 121.1 (overlapping signals, C-M3 and C-M4), 120.3 (overlapping signals, C$_{meso}$), 118.4 (C-M21), 114.2 – 113.9 (overlapping signals, C-M2 and C-M5), 113.3 (C-M17), 112.6 (C-M14), 70.5 – 70.4 (several overlapping signals: C-M9 and C-M10), 69.3 – 68.6 (several overlapping signals C-M12, and C-M11, C-M8, C-M7).

HSQC (400 MHz/101 MHz, DMSO-d$_6$, 298 K): δ (^{1}H) / δ (^{13}C) = 8.89/131.9 – 131.4 ($H_{\beta\text{-pyrrol}}$/$C_{\beta\text{-pyrrol}}$), 8.82 – 8.76/131.9 – 131.4 ($H_{\beta\text{-pyrrol'}}$/$C_{\beta\text{-pyrrol'}}$), 8.28 – 8.13/134.5, 134.2, 118.4 (H-M22/C-M22 and $H_{o\text{-Ph}}$/$C_{o\text{-Ph}}$ and H-M21/C-M21), 7.85 – 7.70/127.6 – 127.4, 126.6, 121.6, 113.3 ($H_{p\text{-Ph}}$/$C_{p\text{-Ph}}$ and $H_{m\text{-Ph}}$/$C_{m\text{-Ph}}$ and H-M15/C-M15 and H-M17/C-M17), 7.16/112.6 (H-M14/C-M14), 6.97 – 6.84/121.4 – 121.1, 114.2 – 113.9 (H-M2, H-M3, H-M4, H-M5/C-M2, C-M3, C-M4 and C-M5), 4.27 – 4.23/69.3 – 68.6 (H-M12/C-M12), 4.21 – 4.16/69.3 – 68.6 (H-M12'/C-M12'), 4.08 – 4.02/69.3 – 68.6 (H-M7/C-M7), 3.87 – 3.82/69.3 – 68.6 (H-M11/C-M11), 3.82 – 3.78/69.3 – 68.6 (H-M11'/C-M11'), 3.77 – 3.72/69.3 – 68.6 (H-M8/C-M8), 3.72 – 3.61/70.5-70.4 (H-M9 and H-M10/ C-M9 and C-M10).

HMBC (400 MHz/101 MHz, DMSO-d$_6$, 298 K): δ (^{1}H) / δ (^{13}C) = 10.50/ 165.3, 118.4 (amide-N*H*/C-M19, C-M21), 8.89/149.5, 149.4 – 149.2, 131.9 – 131.4 ($H_{\beta\text{-pyrrol}}$/$C_{\alpha\text{-pyrrol}}$, $C_{\alpha\text{-pyrrol'}}$, $C_{\beta\text{-pyrrol}}$), 8.82 – 8.76/149.5, 149.4 – 149.2, 131.9 – 131.4 ($H_{\beta\text{-pyrrole}}$/$C_{\alpha\text{-pyrrol}}$, $C_{\alpha\text{-pyrrol'}}$, $C_{\beta\text{-pyrrol}}$), 8.28 – 8.13/138.8, 137.8, 134.5, 134.2, 127.6 – 127.4, 120.3, 118.4 ($H_{o\text{-Ph}}$, H-M22/C-M20, C-M23, C-M22, $C_{o\text{-Ph}}$, $C_{p\text{-Ph}}$, C_{meso} and H-M21/C-M23, C-M21), 7.85 – 7.70/165.3, 151.4, 147.9, 142.8, 134.2, 126.6, 121.6, 113.3 (H-M17/C-M19, C-M13, C-M18, and H-M15/C-M18, C-M16 and $H_{m\text{-Ph}}$/$C_{ipso\text{-}Ph}$, $C_{o\text{-Ph}}$ and $H_{p\text{-Ph}}$/$C_{m\text{-Ph}}$), 7.16/147.9, 127.6 – 127.4, (H-M14/C-M18, C-M16), 6.97 – 6.84/148.6 – 148.4, 121.4 – 121.1 (H-M3, H-M4/C-M6, C-M1 and H-M2, H-M5/C-M-6, C-M1), 4.08 – 4.02 /69.3 – 68.6 (H-M7/C-M8), 3.77 – 3.72/70.5 – 70.4 (H-M8/C-M9), 3.72 – 3.61/69.3 – 68.6 (H-M9 and H-M10/ C-M8 and C-M11).

COSY (400 MHz/400 MHz, DMSO-d$_6$, 298 K): δ (^{1}H) / δ (^{1}H) = 8.89/8.82 – 8.76 ($H_{\beta\text{-pyrrol}}$/$H_{\beta\text{-pyrrol'}}$), 8.82 – 8.76/8.89 ($H_{\beta\text{-pyrrol'}}$/$H_{\beta\text{-pyrrol}}$), 8.28 – 8.13/7.85 – 7.70 ($H_{o\text{-Ph}}$/$H_{m\text{-Ph}}$), 7.85 – 7.70/8.28 – 8.13, 7.16 ($H_{m\text{-Ph}}$/$H_{o\text{-Ph}}$ and H-M15/H-M14), 7.16/7.85 – 7.70 (H-M14/H-M15), 4.27 – 4.23/3.87 – 3.82 (H-M12/H-M11), 4.21 – 4.16/3.82 – 3.78 (H-M12'/H-M11'), 4.08 – 4.02/3.77 – 3.72 (H-M7/H-M8), 3.87 – 3.82/4.27 – 4.23 (H-M11/H-M12), 3.82 – 3.78/4.21 – 4.16 (H-M11'/H-M12'), 3.77 – 3.72/4.08 – 4.02 (H-M8/H-M7).

IR (ATR-FT): $\tilde{\nu}$ = 3049 (w), 2920 (w), 2867 (w), 1671 (w) 1650 (w), 1593.4 (m), 1496.49 (s), 1452 (m), 1440 (m), 1398 (w), 1337 (w), 1314 (m), 1266 (m), 1254 (m), 1202 (m), 1123 (m), 1065 (w), 1052 (w), 993 (s), 927 (w), 795 (s), 744 (m), 718 (m), 701 (m), 659 (w) cm^{-1}.

MS (ESI-pos, MeOH): m/z = 1188.3501 ([M+Na]$^+$, calcd. 1188.3496 for [$C_{69}H_{59}N_5O_9ZnNa$]$^+$); 594.6801 ([M+Na+H]$^{2+}$, calcd. 594.6785 for [$C_{69}H_{60}N_5O_9ZnNa$]$^{2+}$).

Chromatography

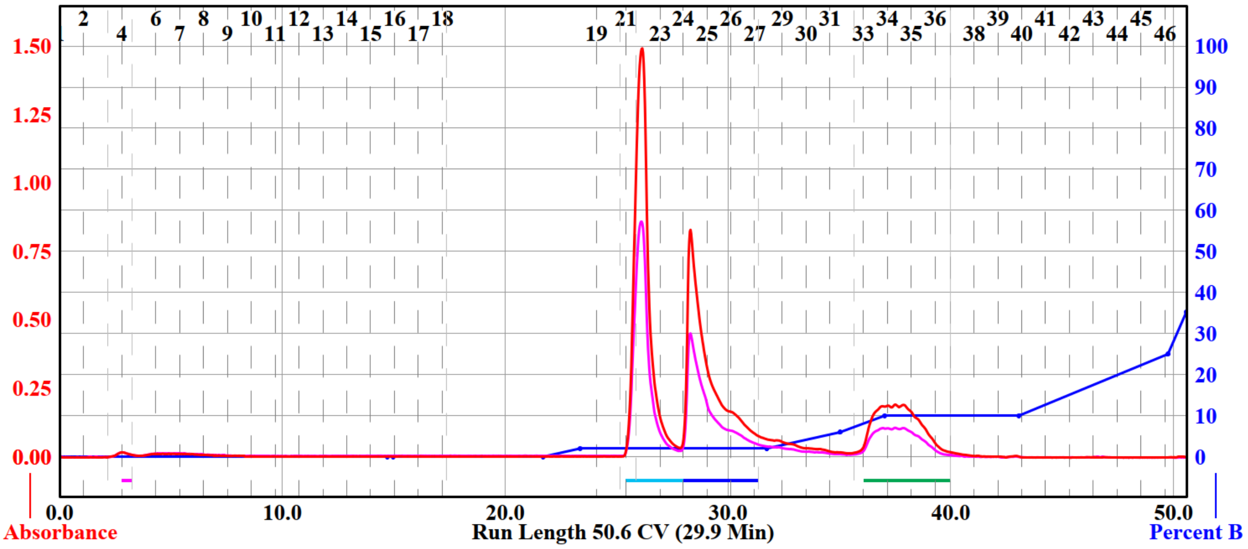

Figure S4: Chromatogram for purification of **5** using a gradient of (A) DCM/(B) methanol with a flowrate of 30 mL/min. Fractions 20 to 24 were combined.

1.2.2.4 General Procedure A: Synthesis of switchable rotaxanes

To a 10 mL Schlenk-tube under argon atmosphere, dibenzylammonium half-thread **4a/b-HPF$_6$** (1 eq.), crown-ether macrocycle **5** (1.2 eq.) and the corresponding azide (1.3 eq.) were added. The Schlenk-tube was evacuated and backfilled with argon three times. Then dichloromethane was added (4 mL per mmol of **4a/b-HPF$_6$**) and the solution was degassed by purging with argon for five minutes. Then tetrakis(acetonitrile)copper(I) hexafluorophosphate (1.5 eq.) was added and the mixture was stirred at room temperature for 23 h, covered in aluminium foil to protect it from light. 3-(Ethylendiamino)-propyl functionalized silica gel (loading 1.4 mmol/g, 10 eq. in respect to added copper) was added and the mixture was stirred for two hours. The mixture was filtered and the crude product was purified by column chromatography using a dichloromethane/methanol gradient. Since not all of the rotaxanes could be obtained as pure compounds, the presence of the interlocked structure was confirmed by mass spectrometry and the impure compounds were used for the methylation and further purified and fully characterized.

For methylation, the triazole containing rotaxane was dissolved in methyl iodide (0.1 mL/mg) and stirred at room temperature under ambient atmosphere with exclusion of light until full conversion of the starting material was observed by thin layer chromatography (2-10 days). Excess methyl iodide was removed *in vacuo* and the methylated compound was purified by flash column chromatography on silica (dichloromethane/methanol gradient) if necessary. The methylated rotaxane was then dissolved in dichloromethane (2 mL) and solid ammonium hexafluorophosphate (20 eq.) was added. The suspension was stirred at room temperature with the exclusion of light for 20 hours, filtered over a polyamide syringe filter and the solvent was removed *in vacuo*.

1.2.2.5 Synthesis of ZnTPP-BHQ-2-rotaxane **1a-H(PF$_6$)$_2$**

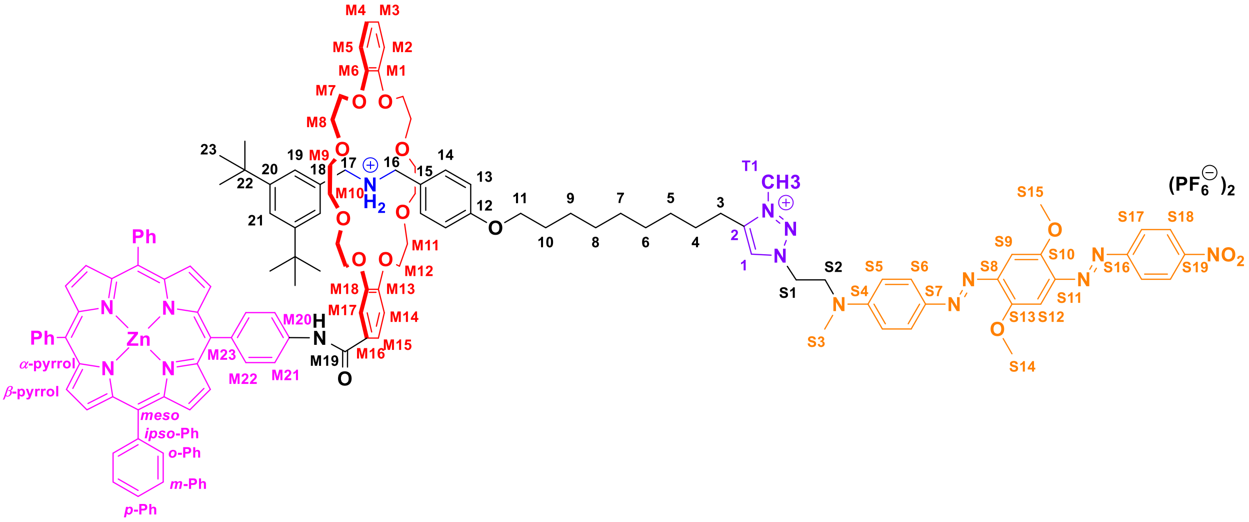

Rotaxane **1a-H(PF$_6$)$_2$** was synthesized according to general procedure A using dibenzylammonium half-thread **4a-HPF$_6$** (30.0 mg, 48.3 µmol, 1 eq.), Zn-porphyrin decorated macrocycle **5** (81.4 mg, 69.7 µmol, 1.4 eq.) and BHQ-2-azide (**6**) (27.0 mg, 55.2 µmol, 1.1 eq.) After purification by column chromatography on a Teledyne Isco NextGen 300+ using a 12g “Redisep gold” silica column with a dichloromethane/methanol gradient (see figure Figure S5), the desired product was obtained as a purple glass (67.5 mg, 27.7 µmol, 57.3% yield). The rotaxane (78.4 mg, 34.4 µmol) was methylated (reaction time 10 days, purification by column chromatography, see Figure S6). After anion exchange, the desired compound was obtained as a purple glass (15.3 mg, 6.27 µmol, 18.2% yield).

$C_{126}H_{135}N_{15}O_{14}Zn(PF_6)_2$: 2438,8654 g/mol

^{1}H-NMR (400 MHz, CD_2Cl_2, 298 K): δ = 8.92 (d, *J* = 4.7 Hz, 2H, H$_{\beta\text{-pyrrole}}$), 8.89 – 8.82 (m, 6H, H$_{\beta\text{-pyrrole'}}$), 8.41 (s, 1H, amide-N*H*), 8.26 – 8.11 (m, 10H, H$_{o\text{-Ph}}$ and H-M22 and H-S18), 7.90 – 7.70 (m, 14H, H$_{m\text{-Ph}}$ and H$_{p\text{-Ph}}$ and H-S17 and H-M21 and H-1), 7.56 (d, *J* = 8.7 Hz, 2H, H-S6), 7.51 – 7.40 (m, 3H, N*H*$_2$ and H-21), 7.32 (d, *J* = 1.9 Hz, 2H, H-19), 7.29 (d, *J* = 8.6 Hz, 1H, H-M15), 7.17 (s, 1H, H-M17), 7.04 (d, *J* = 8.2 Hz, 2H, H-14), 6.90 – 6.70 (m, 7H, H-M2, H-M3, H-M4 and H-M5, H-M14, H-S9, H-S12), 6.50 – 6.40 (m, 4H, H-13, H-S5), 4.73/53.3 (ps t, *J* = 6.6 Hz, 2H, H-17), 4.51 (ps t, *J* = 6.6 Hz, 2H, H-16), 4.38 (ps t, *J* = 6.2 Hz, 2H, H-S1), 4.27 – 4.16 (m, 2H, H-glycol),, 4.13 – 4.00 (m, 6H, H-glycol), 3.88– 3.65 (m, 16H, H-11, H-S2, H-glycol), 3.64 (s, 3H, H-S15), 3.57 – 3.41 (m, 10H, H-S14, and H-T1 and H-glycol), 2.88 (s, 3H, H-S3), 2.20 – 2.07 (m, 2H, H-3), 1.70 – 1.60 (m, 2H, H-10), 1.43 – 1.09 (m, 34H, H-4 to H-9 and H-23; the observed integral is slightly too large, probably caused by overlap with residual grease). H-M7 to H-M12 could not individually be assigned and are grouped together and abbreviated as H-glycol.

^{13}C{^{1}H}-NMR (101 MHz, CD_2Cl_2, 298 K): δ = 165.4 (C-M19), 160.2 (C-12), 156.6 (C-S16), 153.2 and 151.0 (C-S10 and C-S13), 152.2 (C-20), 151.5 (C-S4), 150.7 – 150.4 (overlapping signals, C$_{\alpha\text{-pyrrole}}$, C-M13), 148.8 (C-S19), 147.7 (C-M18), 145.8 (overlapping signals, C-M1, C-M6 and C-S11), 145.3 (C-S7), 145.2 (C-2), 143.4 (C$_{ipso\text{-Ph}}$), 142.2 (C-S8), 139.1 (C-M23), 138.4 (C-M20), 135.5 (C-M22), 135.1 (C$_{o\text{-Ph}}$), 132.5 – 132.1 (overlapping signals, C$_{\beta\text{-pyrrol}}$, C-18), 131.2 (C-14), 128.2 – 127.8 (overlapping signals, C$_{p\text{-Ph}}$ and C-1 and C-M16), 127.2 (C$_{m\text{-Ph}}$), 126.0 (C-S6), 125.0 (C-S18), 124.2 – 123.7 (overlapping signals, C-21 and C-19 and C-S17), 123.4 (C-15), 122.1

(overlapping signals, C-M3, C-M4), 121.5 (C_{meso}), 121.2 (C-M15), 118.8 (C-M21), 114.7 (C-13), 113.2 – 112.9 (overlapping signals, C-M2, C-M5), 112.2 (C-M14), 112.1 – 111.8 (overlapping signals, C-M17 and C-S5), 100.6 (C-S12), 99.9 (C-S9), 71.6 – 70.4 (overlapping signals, C-glycol), 69.1 – 68.1 (overlapping signals, C-glycol and C-11), 56.9 (C-S15), 56.8 (C-S14), 53.3 (overlapping with solvent signal, C-17 assigned by HSQC), 52.9 (C-16), 51.3 (C-S2), 51.1 (C-S1), 37.4 (C-T1), 38.6 (C-S3), 35.4 (C-22), 31.7 (C-23), 30.3, 29.7, 29.6, 29.2, 29.1 (C-10 and C-8 to C-5), 26.8 (C-4), 26.4 (C-9), 23.3 (C-3).

^{19}F{^{1}H}-NMR (376 MHz, CD_2Cl_2, 298 K): δ = -72.6 (d, *J* = 712.0 Hz, PF_6^-).

^{31}P{^{1}H}-NMR (162 MHz, CD_2Cl_2, 298 K): δ = -144.5 (sept, *J* = 712.0 Hz, PF_6^-).

HSQC (400 MHz /101 MHz, CD_2Cl_2, 298 K): δ (^{1}H) / δ (^{13}C) = 8.92/132.5 – 132.1 ($H_{\beta\text{-pyrrole}}$ / $C_{\beta\text{-pyrrole}}$), 8.89 – 8.82/132.5 – 132.1 ($C_{\beta\text{-pyrrole'}}$/ $C_{\beta\text{-pyrrole'}}$), 8.28 – 8.13/135.5, 135.1, 125.0 (C-M22/H-M22 and $H_{o\text{-Ph}}$/$C_{o\text{-Ph}}$ and H-S18/C-S18), 7.90 – 7.70/128.2 – 127.8, 127.2, 124.2 – 123.7, 118.8 ($H_{p\text{-Ph}}$/$C_{p\text{-Ph}}$ and H-1/C-1 and $H_{m\text{-Ph}}$/$C_{m\text{-Ph}}$ and H-S17/C-S17 and H-M21/C-M21), 7.16/126.0 (H-S6/C-S6), 7.51 – 7.40/124.2 – 123.7 (H-21/C-21), 7.32/124.2 – 123.7 (H-19/C-19), 7.29/121.2 (H-M15/C-M15), 7.17/112.1 – 111.8 (H-M17/C-M17), 7.04/131.2 (H-14/C-14), 6.90 – 6.70/122.1, 113.2 – 112.9, 112.2, 100.6, 99.9 (H-M3, H-M4/C-M3, C-M4 and H-M2, H-M5/C-M2, C-M5 and H-M14/C-M14 and H-S9, H-S12/C-S9, C-S12), 6.50 – 6.40/114.7, 112.1 – 111.8 (H-13/C-13 and H-S5/C-S5), 4.73/53.3 (H-17/C-17), 4.51/52.9 (H-16/C-16) 4.38/51.1 (H-S1/C-S1), 4.21 – 4.16/69.1 – 68.1 (H-glycol/C-glycol), 4.13 – 4.00/69.1 – 68.1 (H-glycol/C-glycol), 3.87 – 3.82/71.6 – 70.4, 69.1 – 68.1, 51.1, 51.3 (H-glycol/C-glycol, H 11/C-11, H-glycol/C-glycol, H-S2/C-S2), 3.64/56.9 (H-S15/C-S15), 3.57 – 3.41/71.6 – 70.4, 56.8, 37.4 (H-glykol/C-glykol, H-S14 /C-S14, H-T1/C-T1), 2.88/38.6 (H-S3/C-S3), 2.20 – 2.07/23.3 (H-3/C-3), 1.70 – 1.60/29.6 (H-10/C-10), 1.43 – 1.09/31.7, 30.3, 29.7, 29.2, 29.1, 26.8, 26.4 (H-23/C-23 and H-10 to H-4/C-10 to C-4).

HMBC (400 MHz /101 MHz, CD_2Cl_2, 298 K): δ (^{1}H) / δ (^{13}C) = 8.89/150.7 – 150.4, 132.5 – 132.1 ($H_{\beta\text{-pyrrol}}$/$C_{\alpha\text{-pyrrol}}$, $C_{\alpha\text{-pyrrol'}}$, $C_{\beta\text{-pyrrol}}$, $C_{\beta\text{-pyrrol'}}$), 8.89 – 8.82/150.7 – 150.4, 132.5 – 132.1, 121.5 ($H_{\beta\text{-pyrrole'}}$/$C_{\alpha\text{-pyrrol'}}$, $C_{\alpha\text{-pyrrol}}$, $C_{\beta\text{-pyrrol'}}$, $C_{\beta\text{-pyrrol}}$, C_{meso}), 8.41/165.4, 118.8 (amide-N*H*/C-M19, C-M21), 8.26 – 8.11/156.6, 148.8, 138.4, 135.5, 135.1, 128.2 – 127.8 (H-S18/C-S16, C-S19 and H-M22/C-M20, C-M22 and $H_{o\text{-Ph}}$/$C_{o\text{-Ph}}$, $C_{p\text{-Ph}}$), 7.90 – 7.70/148.8, 145.2, 143.4, 139.1, 135.1, 127.2, 124.2 – 123.7, 118.8 (H-S17/C-S19 and H-1/C-2 and $H_{m\text{-Ph}}$/$C_{ipso\text{-Ph}}$ and H-M21/C-M23 and $H_{p\text{-Ph}}$/$C_{o\text{-Ph}}$ and $H_{m\text{-Ph}}$/$C_{m\text{-Ph}}$ and H-S17/C-S17 and H-M21/C-M21), 7.56/151.5, 145.3, 126.0 (H-S6/C-S4, C-S7, C-S6), 7.51 – 7.40/124.2 – 123.7, 35.4 (H-21/C-19, C-22), 7.32/124.2 – 123.7, 53.3, 35.4 (H-19/C-21, C-17, C-22), 7.29/165.4, 150.7 – 150.4, 112.1 – 111.8 (H-M15/C-M19, C-M13, C-M17) 7.17/150.7 – 150.4, 147.7, 121.2 (H-M17/C-M13, C-M18, C-M15), 7.04/160.2, 131.2, 52.9 (H-14/C-12, C-14, C-16), 6.90 – 6.70/153.2, 151.0, 147.7, 145.8, 142.2, 128.2 – 127.8, 122.1, 113.2 – 112.9 (H-S9, H-S12/C-S10, C-S13 and H-M14/C-M18 and H-M3, H-M4/C-M1, C-M6, C-M2, C-M5 and H-M2, H-M5/C-M3, C-M4 and H-S9/C-S11 and H-S12/C-S8 and H-M14/C-M16), 6.50 – 6.40/160.2, 145.3, 123.4, 114.7, 112.1 – 111.8 (H-13/C-12 and H-S5/C-S7 and H-13/C-15, C-13 and H-S5/C-S5), 4.73/132.5 – 132.1, 124.2 – 123.7 (H-17/C-18, C-19), 4.51/131.2, 123.4 (H-16/C-14, C-15), 3.88 – 3.65/160.2, 71.6 – 70.4, 38.6, 29.6, 26.4 (H-11/C-12, H-glycol/C-glycol, H-S2/C-S3, C-10, C-9), 3.64/153.2 (H-S15/C-S10), 3.57 – 3.41/151.0, 145.2, 71.6 – 70.4 (H-S14 /C-S13 and H-T1/C-2 and H-glycol/C-glycol), 2.88/151.5, 51.3 (H-S3/C-S4, C-S2), 1.70 – 1.60/69.1 – 68.1, 26.4 (H-10/C-11, C-9), 1.43 – 1.09/152.2, 35.4, 31.7 (H-23/C-20, C-22, C-23).

COSY (400 MHz /400 MHz, CD_2Cl_2, 298 K): δ (^{1}H) / δ (^{1}H) = 8.26 – 8.11/7.90 – 7.70 ($H_{o\text{-Ph}}$/$H_{m\text{-Ph}}$ and H-S18/H-S17 and H-M22/H-M21), 7.90 – 7.70/8.26 – 8.11 ($H_{m\text{-Ph}}$/$H_{o\text{-Ph}}$ and H-S17/H-S18 and H-M21/H-M22), 7.56/6.50 – 6.40 (H-S6/H-S5), 7.51 – 7.40/7.32, 4.73, 4.51 (H-21/H-19 and NH_2/H-17, H-16), 7.32/7.51 – 7.40 (H-19/H-21), 7.29/6.90 – 6.70 (H-M15/H-M14), 7.04/6.50 – 6.40 (H-14/H-13), 6.90 – 6.70/7.29 (H-M14/H-M15), 6.50 – 6.40/7.56, 7.04 (H-S5/H-S6 and H-13/H-14), 4.73/7.51 – 7.40 (H-17/NH_2), 4.51/7.51 – 7.40 (H-16/NH_2), 4.38/3.88 – 3.65 (H-S1/H-S2), 4.27 – 4.16/4.13 – 4.00 (H-glycol/H-glycol), 4.13 – 4.00/4.27 – 4.16, 3.88– 3.65 (H-glycol/ H-glycol), 3.88– 3.65/4.38, 4.13 – 4.00, 3.57 – 3.41, 1.70 – 1.60 (H-S2/H-S1 and H-glycol/H-glycol and H-11/H-10), 3.57 – 3.41/3.88– 3.65 (H-glycol/H-glycol), 2.20 – 2.07/1.43 – 1.09 (H-3/H-4), 1.70 – 1.60/3.88 – 3.65, 1.43 – 1.09 (H-10/H-11, H-9), 1.43 – 1.09/2.20 – 2.07, 1.70 – 1.60 (H-4/H-3 and H-10/H-9).

IR (ATR-FT): $\tilde{\nu}$ = 3312 (broad), 2954 (m), 2886 (w), 1596 (w), 1508 (w), 1457 (w), 1439 (w), 1366 (w), 1340 (m), 1319 (w), 1248 (w), 1191 (w), 1158 (w), 1098 (m), 1064 (s), 1036 (s), 962 (m), 925 (m), 844 (s), 751 (m), 558 (m) cm^{-1}.

MS (ESI-pos, MeOH): m/z = 1072.9803 ($[M\text{-}2(PF_6)]^{2+}$, calcd. 1072.9797 for $[C_{126}H_{135}N_{15}O_{14}Zn]^{2+}$).

Chromatography (before methylation)

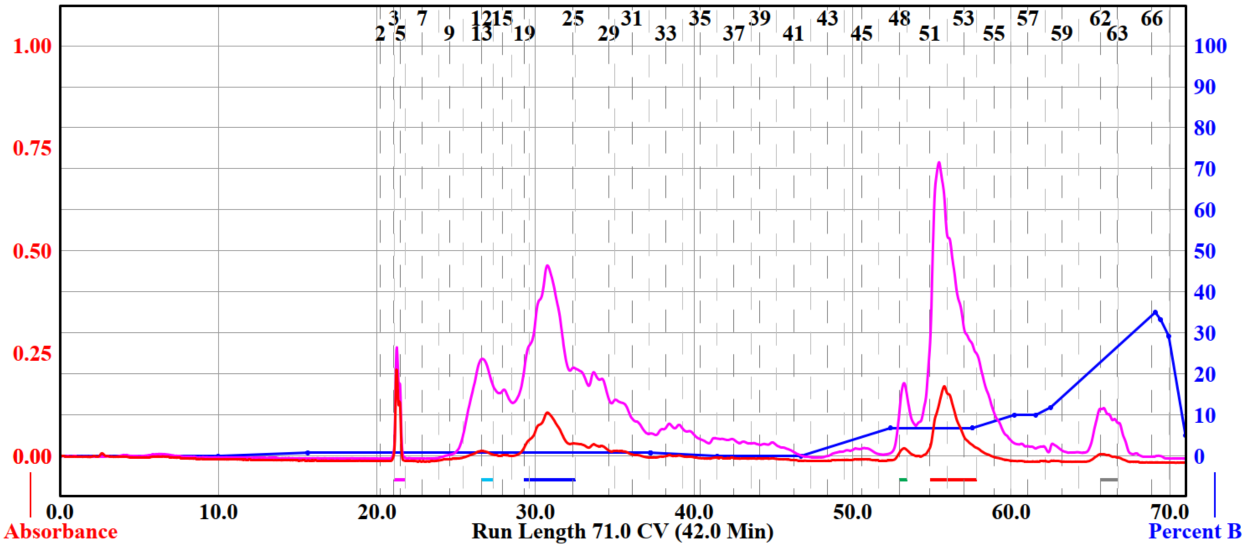

Figure S5: Chromatogram for purification of the unmethylated rotaxane using a gradient of (A) DCM/(B) methanol with a flowrate of 20 mL/min; fractions 21 to 31 were combined.

Chromatography (after methylation)

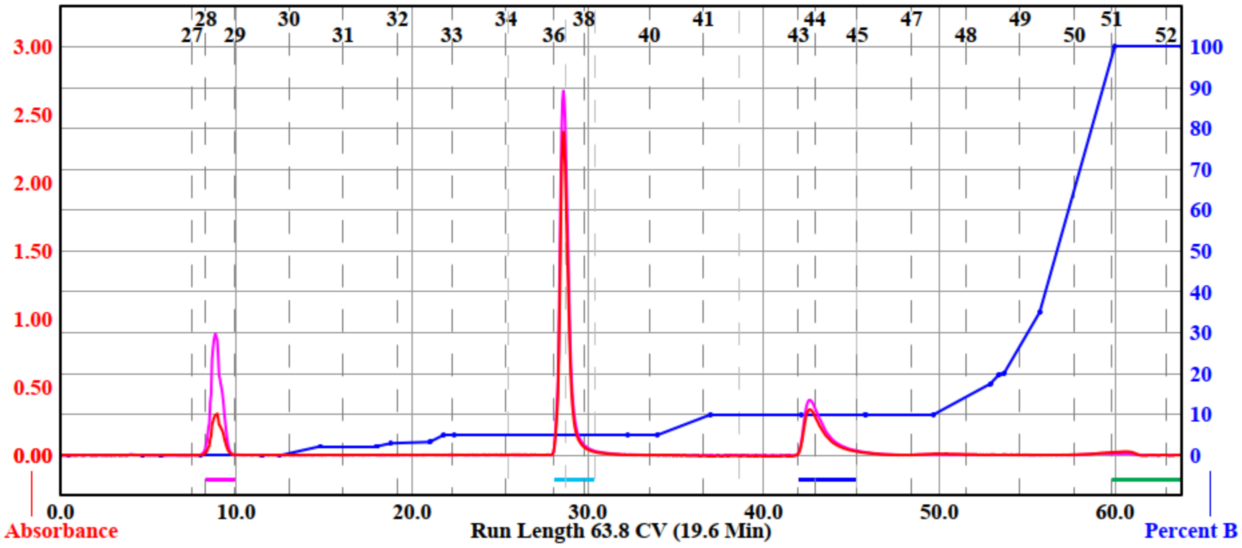

Figure S6: Chromatogram for purification of rotaxane **H-1a**$^{2+}$ after methylation using a gradient of (A) DCM/(B) methanol with a flowrate of 20 mL/min; fractions 42 to 44 were combined.

1.2.2.6 Synthesis of short ZnTPP-BHQ-2-rotaxane **1b-H(PF$_6$)$_2$**

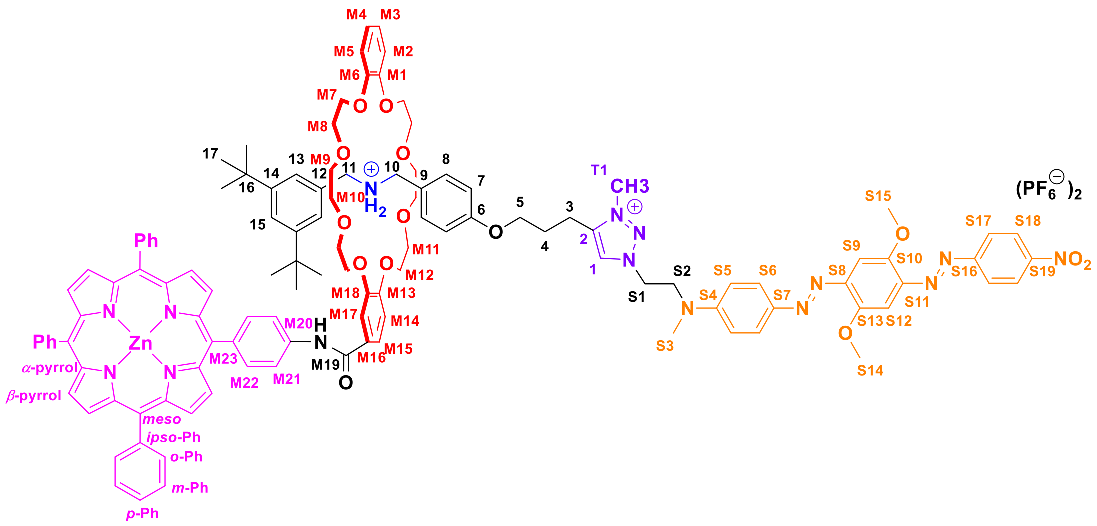

Rotaxane **1b** was synthesized according to general procedure A using dibenzylammonium half-thread **4b-PF$_6$** (20.9 mg, 38.9 µmol, 1 eq.), Zn-porphyrin decorated macrocycle **5** (58.9 mg, 50.4 µmol, 1.3 eq.) and BHQ-2-azide (**6**) (21.0 mg, 42.9 µmol, 1.1 eq.) After purification by column chromatography on a Teledyne Isco NextGen 300+ using a 6g "Chromabond gold" silica column with a dichloromethane/methanol gradient (see figure Figure S7), the desired product was obtained as a mixture containing unreacted macrocycle **5** (52.3 mg, approximately 70% purity, 44% yield). The crude product (36.0 mg, approximately 70% purity, 11.4 µmol) was methylated (reaction time 48 hours) and further purified by manual column chromatography (2 x 20 cm) on silica gel with a dichloromethane/methanol gradient. After ion exchange, the desired product (8.2 mg, 30.7% in respect to 11.4 µmol unmethylated rotaxane) was obtained as a purple film.

$C_{120}H_{123}N_{15}O_{14}Zn$-$(PF_6)_2$: 2354.7034 g/mol

^{1}H-NMR (600 MHz, CD_2Cl_2, 298 K): δ = 8.90 – 8.80 (m, 8H, $H_{\beta\text{-pyrrole}}$), 8.59 (s, 1H, amide-N*H*), 8.26 – 8.14 (m, m, 12H, $H_{o\text{-Ph}}$ and H-M22 and H-M21 and H-S18), 8.09 – 8.05 (m, 2H, H_{Aryl}), 7.85 – 7.70 (m, 11H, $H_{m\text{-Ph}}$ and $H_{p\text{-Ph}}$ and H-S17), 7.66 – 7.50 (m, 3H, H-M15 and NH_2), 7.50 – 7.37 (m, 4H, H-S6, H-15, H-M17), 7.36 – 7.31 (m, 2H, H-13), 7.30 – 7.09 (m, 3H, H-1 and 2xH_{Aryl}), 7.07 (d, *J* = 8.2 Hz, 2H, H-8), 6.90 – 6.80 (m, 5H, H-M2 to H-M5 and H-M14), 6.56 – 6.41 (m, 6H, H-7, H-S5, H-S9, H-S12), 4.73 (ps t, *J* = 6.8 Hz, 2H, H-11), 4.66 – 4.60 (m, 4H H-5, H-S2), 4.54 (ps t, *J* = 6.3 Hz, 2H, H-10), 4.38 – 4.21 (m, 2H, H-glycol), 4.20 – 3.95 (m, 11H, H-glycol, H-T1), 3.94 – 3.65 (m, 18H, H-glycol, H-4, H-S1), 3.61-3.51 (m, 4H, H-glycol), 3.50 – 3.37 (m, 5H, H-glycol, H-S15), 3.29 (s, 3H, H-S14), 2.86 (s, 3H, H-S3), 2.10 (ps t, *J* = 6.5 Hz, 2H, H-3), 1.25 – 1.13 (s, 18H, H-17). H-M7 to H-M12 could not individually be assigned and are grouped together and abbreviated as H-glycol. The sum of integrals for the glycol signals is slightly too large.

^{13}C{^{1}H}-NMR (151 MHz, CD_2Cl_2, 298 K): δ = 165.9 (C_{quart}), 165.7 (C-M19), 159.4 (C-6), 156.6 (C-S16), 153.0 (C-S10), 152.3 (C-14), 151.3 (C-S4), 150.7 – 150.4 (several overlapping signals, $C_{\alpha\text{-pyrrole}}$, C-S13, C-M13), 148.9 (C-S19), 148.4 – 148.3 (overlapping signals, C-M1 and C-M6), 147.5 (no HMBC or HSQC cross-peak detected. Possibly C-M18), 145.6 (C-S11), 145.3 (C-S7), 145.1 (C-2), 143.6 (overlapping signals, $C_{ipso\text{-}Ph}$), 141.9 (no HSQC or HMBC-cross peaks detected, possibly C-S8), 139.3 (no HSQC or HMBC-cross peaks detected, possibly C-M23), 138.6 (C-M20), 135.5 (C-M22), 135.1 ($C_{o\text{-Ph}}$), 134.5 – 134.3 (multiple signals), 132.5 – 132.1 (multiple signals, $C_{\beta\text{-}}$

pyrrole), 132.1 (C-12), 131.3 (C-8), 130.5 (C_{Aryl}), 130.3 (C_{Aryl}, no HMBC or HSQC-cross peak detected), 129.8 (C-1), 129.5 (C_{Aryl}), 128.8 (no HMBC cross-peaks detected, possibly C-M18), 128.0 ($C_{p\text{-Ph}}$), 127.1 (overlapping signals, $C_{m\text{-Ph}}$), 126.0 (C-S6), 125.8 (C_{Aryl}, no HSQC or HMBC-cross peaks detected), 125.1 (C-S18), 124.1 – 123.9 (C-S17 and C-13, C15 and C-9), 122.1 (C-M3, C-M4), 122.0 (C-M15), 121.6 – 121.5 (overlapping signals, C_{meso}), 119.3 (C-M21), 114.9 (C-7), 113.2 – 112.9 (overlapping signals, C-M2, C-M5), 112.2 (C-M14), 111.9 (overlapping signals, C-S5, C-M17), 100.4 (C-S12), 99.8 (C-S9), 71.6 – 70.5 and 68.9 – 66.6 (C-glycol), 63.6 (C-5), 56.8 – 56.7 (overlapping signals, C-S14 and C-S15), 53.4 (C-11), 52.9 (C-10), 51.3 (C-S2), 51.4 (C-S1), 41.0 (C-4), 38.8 (C-S3), 38.2 (C-T1), 35.4 (C-16), 31.7 (C-17), 26.7 (C-3).

The following positions could not be unambiguously assigned: C-M18, C-M16, C-M23, C-S8.

The following signals could not be unambiguously assigned: 165.9 (C_{quart}), 147.5 (possibly C-M18), 141.9 (possibly C-S8) 139.9 (possibly C-M23), 134.5 – 134.3 (C_{Aryl}), 130.5 (C_{Aryl}), 130.3 (C_{Aryl}), 129.5 (C_{Aryl}), 128.8 (possibly C-M18), 125.8 (C_{Aryl})

The unexpected number of signals could be due to amide-rotamers caused by tight folding.

^{19}F{^{1}H}-NMR (376 MHz, CD_2Cl_2, 298 K) δ = -72.5 (d, *J* = 711.8 Hz, P*F*$_6^-$).

^{31}P{^{1}H}-NMR (162 MHz, CD_2Cl_2, 298 K) δ = -144.4 (sept, *J* = 711.8 Hz, *P*F$_6^-$).

HSQC (600 MHz /151 MHz, CD_2Cl_2, 298 K): δ (^{1}H) / δ (^{13}C) = 8.89/132.5 – 132.1 ($H_{\beta\text{-pyrrole}}$/$C_{\beta\text{-pyrrole}}$), 8.26 – 8.14/135.5, 135.1, 125.1, 119. (H-M22/C-M22 and $H_{o\text{-Ph}}$/$C_{o\text{-Ph}}$ and H-S18/C-S18 and H-M21/C-M21), 8.09 – 8.05/130.3 (H_{Aryl}/C_{Aryl}), 7.90 – 7.70/128.0, 127.1, 124.1 – 123.9 ($H_{p\text{-Ph}}$/C*p*-Ph and $H_{m\text{-Ph}}$/$C_{m\text{-Ph}}$ and H-S17/C-S17), 7.66 – 7.50/122.0 (H-M15/C-M15), 7.50 – 7.37/126.0, 124.1 – 123.9, 111.9 (H-S6/C-S6 and H-15/C-15 and H-M17/C-M17), 7.36 – 7.31/124.1 – 123.9 (H-13/C-13), 7.30 – 7.09 /129.8, 128.8, 129.5 (H-1/C-1, 2x H_{Aryl}/C_{Aryl}), 7.07/131.3 (H-8/C-8), 6.90 – 6.80/122.1, 113.2 – 112.9, 112.2 (H-M3, H-M4/C-M3, C-M4 and H-M2, H-M5/C-M2, C-M5 and H-M14/C-M14), 6.56 – 6.41/114.9, 111.9, 100.4, 99.8 (H-7/C-7 and H-S5/C-S5 and H-12/C-S12 and H-S9/C-S9), 4.73/53.4 (H-11/C-11), 4.66 – 4.60/63.6, 51.3 (H-5/C-5, H-S2/C-S2), 4.54/52.9 (H-10/C-10), 4.21 – 4.16/68.9 – 66.6 (H-glycol /C-glycol), 4.20 – 3.95/68.9 – 66.6, 38.2 (H-glycol/C-glycol, and H-T1/C-T1), 3.94– 3.65/71.6 – 70.5 and 68.9 – 66.6, 51.4, 41.0 (H-glycol/C-glycol and, H-S1/C-S1, H-4/C-4), 3.61 – 3.51/71.6 – 70.5 (H-glycol/C-glycol), 73.50 – 3.37/71.6 – 70.5 56.8 – 56.7 (H-glycol/C-glycol and H-S15/C-S15), 3.29/56.8 – 56.7 (H-S14/C-14), 2.86/38.8 (H-S3/C-S3), 2.10/26.7 (H-3/C-3), 1.25 – 1.13 /31.7 (H-17/C-17).

HMBC (600 MHz /151 MHz, CD_2Cl_2, 298 K): δ (^{1}H) / δ (^{13}C) = 8.90 – 8.80/150.7 – 150.4, 132.5 – 132.1 ($H_{\beta\text{-pyrrole}}$/$C_{\alpha\text{-pyrrole}}$, $C_{\beta\text{-pyrrole}}$), 8.26 – 8.14/156.6, 148.9, 138.6, 135.5, 135.1, 128.0, 121.6 – 121.5 H-S18/C-S16, C-S19 and H-M22/C-M20, C-M22 and $H_{o\text{-Ph}}$/$C_{o\text{-Ph}}$, C*p*-Ph, C_{meso}), 8.09 – 8.05/165.9, 134.4 (H_{Aryl}/C_{quart}, C_{Aryl}), 7.90 – 7.70/148.9, 143.6, 127.1, 124.1 – 123.9 (H-S17/C-S19, C-S11 and $H_{m\text{-Ph}}$/$C_{m\text{-Ph}}$ and H-S17/C-S17), 7.50 – 7.37/151.3, 150.7 – 150.4, 145.2, 126.0, 124.1 – 123.9, 35.4 (H-S6/C-S4 and H-M17/C-M13 and H-S6/C-S7, C-S6 and H-15/C-13, C-16), 7.36 – 7.31/124.1 – 123.9, 53.4, 35.4 (H-13/C-15, C-11, C-16), 7.30 – 7.09/129.8, 41.0 (H-1/C-1, C-4), 7.07/159.4, 131.3, 52.9 (H-8/C-6, C-8, C-10), 6.90 – 6.80/122.1 (H-M5/C-M3 and H-M2/C-M4), 6.56 – 6.41/145.3, 124.1 – 123.9 (H-S5/C-S7 and H-7/C-9), 4.73/132.1, 124.1 – 123.9 (H-11/C-12, C-13), 4.51/131.3, 124.1 – 123.9 (H-10/C-8, C-9), 3.88– 3.65/129.8 (H-S1/C-1), 3.50 – 3.37/153.0 (H-S15/C-S10), 3.29/150.7 – 150.4 (H-S14/C-S12), 2.86/151.3 (H-S3/C-S4), 1.25 – 1.13/152.3, 35.4, 31.7 (H-17/C-14, C-16, C-17).

COSY (600 MHz /600 MHz, CD_2Cl_2, 298 K): δ (^{1}H) / δ (^{1}H) = 8.26 – 8.14 /7.85 – 7.70 ($H_{o\text{-Ph}}$/$H_{m\text{-Ph}}$ and H-S18/H-S17), 7.85 – 7.70/8.26 – 8.14 (H-S17/H-S18 and $H_{m\text{-Ph}}$/$H_{o\text{-Ph}}$), 7.66 – 7.50/6.90 – 6.80, 4.74, 4.54 (H-M15/H-M14 and NH_2/H11, H-10), 7.50 – 7.37/6.56 – 6.41 (H-S6/H-S5), 7.07/6.56 – 6.41 (H-8/H-7), 6.90 – 6.80/7.66 – 7.50 (H-M14/H-M15), 6.56 – 6.41/7.50 – 7.37, 7.07 (H-S5/H-S6 and H-7/H-8), 4.73/7.66 – 7.50 (H-11/NH_2), 4.66 – 4.60/3.94 – 3.65 (H-5/H-4), 4.54/7.66 – 7.50 (H-10/NH_2), 4.27 – 4.16/4.20 – 3.95 (H-glycol /H-glycol), 4.20 – 3.95/4.38 – 4.21 (H-M12/H-M11), 3.94 – 3.65/3.61 – 3.51, 3.50 – 3.37, 2.10 (H-glycol/ H-glycol and H-4/H-3), 3.61-3.51/3.94 – 3.65 (H-glycol / H-glycol), 3.50 – 3.37/3.94 – 3.65 (H-glycol /H-glycol), 2.10/3.94 – 3.65 (H-3/H-4), 1.25 – 1.13/152.3, 35.4, 31.7 (H-17/C-14, C-16, C-17).

IR (ATR-FT): $\tilde{\nu}$ = 2956 (m), 2927 (w), 2889 (w), 1722 (w), 1596 (m), 1512 (m), 1455 (w), 1443 (w), 1366 (w), 1341 (w), 1319 (w), 1249 (m), 1203 (w), 1101 (m), 1066 (s), 958 (m), 927 (m), 845 (s), 559 (w) cm^{-1}.

MS (ESI-pos, MeOH): m/z = 2060.8533 ($[M\text{-}H\text{-}2(PF_6)]^+$, calcd. 2060.8582 for $[C_{120}H_{122}N_{15}O_{14}Zn]^+$); 1030.9315 ($[M\text{-}2(PF_6)]^{2+}$, calcd. 1030.9327 for $[C_{120}H_{123}N_{15}O_{14}Zn]^{2+}$).

Chromatography (before methylation)

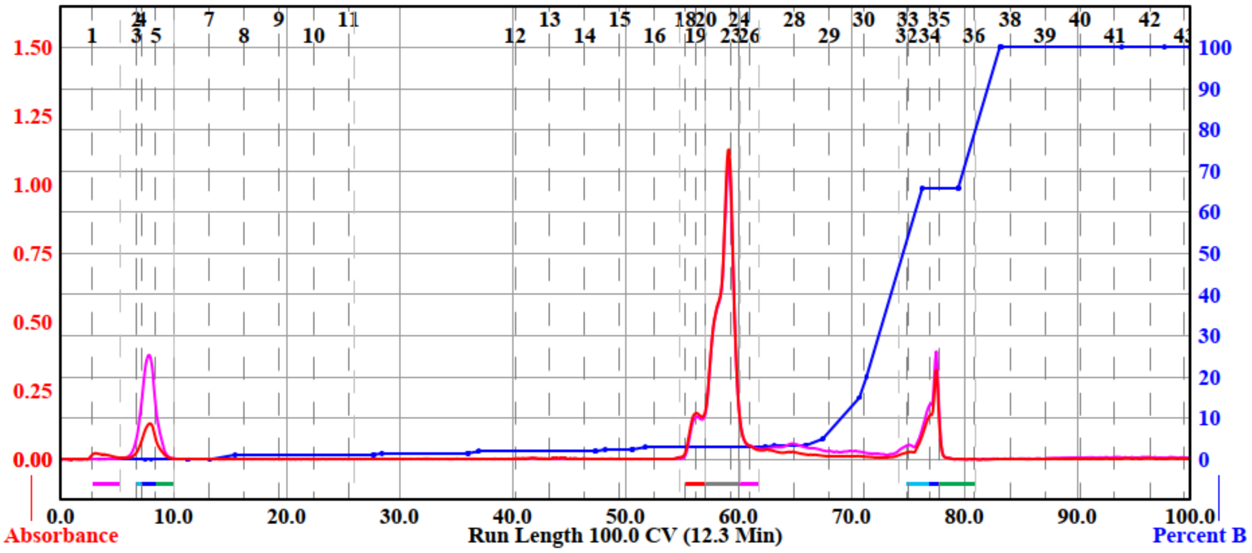

Figure S7: Chromatogram for purification of the unmethylated rotaxane using a gradient of (A) DCM/(B) methanol with a flowrate of 30 mL/min; fractions 20 to 23 were combined.

1.2.2.7 Synthesis of ZnTPP-Dummy-rotaxane (**2-H(PF6)**$_2$)

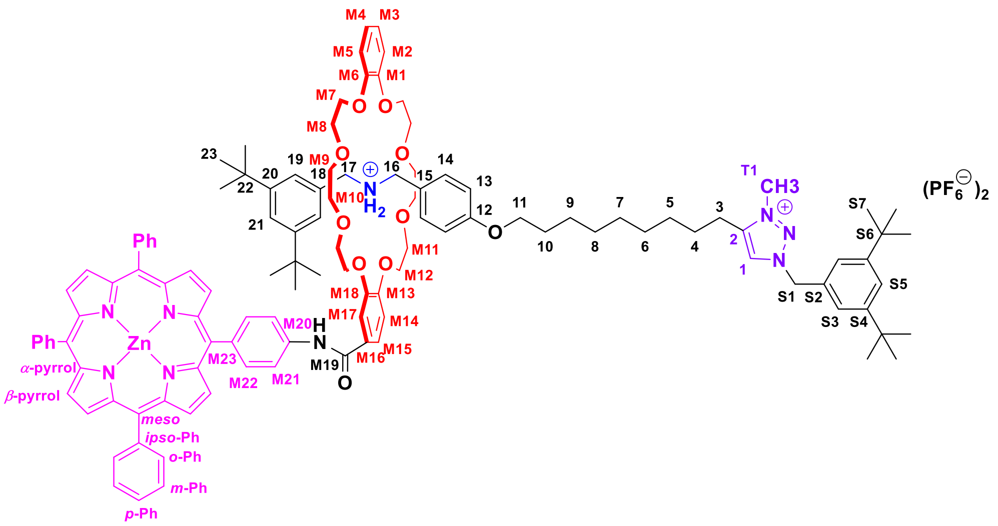

Rotaxane **2** was synthesized according to general procedure A using dibenzylammonium half-thread **4a-HPF**$_6$ (22.0 mg, 35.4 µmol, 1 eq.), Zn-porphyrin decorated macrocycle **5** (52.8 mg, 45.2 µmol, 1.3 eq.) and azide **S19** (see Figure S3) (11.2 mg, 45.6 µmol, 1.3 eq.). After purification by column chromatography on a Teledyne Isco NextGen 300+ using a 4g "Chromabond gold" silica column with a dichloromethane/methanol gradient (see Figure S8) the desired product was obtained as a purple glass (42.9 mg, 21.1 µmol, 59.6% yield). The obtained rotaxane (42.7 mg, 21.0 µmol) was dissolved in methyl iodide (reaction time 4 days, no further purification by column chromatography). After ion exchange, the desired compound was obtained as a purple glass (46.0 mg, 21.0 µmol, quantitative yield).

$C_{118}H_{135}N_9O_{10}Zn(PF_6)_2$ 2194.7394 g/mol

^{1}H-NMR (400 MHz, CD_2Cl_2, 298 K): δ = 9.00 (d, J = 4.6 Hz, 2H, $H_{β\text{-pyrrol}}$), 8.95-8.91 (m, 6H, $H_{β\text{-pyrrol'}}$), 8.45 (s, 1H, amide-N*H*), 8.26 – 8.16 (m, 8H, $H_{o\text{-Ph}}$, H-M22), 8.07 (d, J = 8.4 Hz, 2H, H-M21), 7.83-7.71 (m, 9H, $H_{m\text{-Ph}}$, $H_{p\text{-Ph}}$), 7.57 – 7.48 (m, 3H, NH_2 and H-M15), 7.44 (ps t, J = 1.4 Hz, 1H, H-21), 7.42 (ps t, J = 1.5 Hz, 1H, H-S5), 7.38 (d, J = 1.4 Hz, 2H, H-M17), 7.33 (d, J = 1.3 Hz, 2H, H-19), 7.22 (s, 1H, H-1), 7.09 (d, J = 8.4 Hz, 2H, H-14), 6.99 (d, J = 1.7 Hz, 2H, H-S3), 6.93 – 6.76 (m, 5H, H-M2 to H-M5, H-M14), 6.52 (d, J = 8.4 Hz, 2H, H-13), 4.92 (s, 2H, H-S1), 4.76 (ps t, J = 6.6 Hz, 2H, H-17), 4.54 (ps t, J = 6.5 Hz, 2H, H-16), 4.32 – 4.22 (m, 2H, H-glycol), 4.17 – 4.15 (m, 6H, H-glycol), 3.91 – 3.65 (m, 14H, H-glycol, H-11), 3.60 – 3.43 (m, 4H, H-glycol), 3.32 (s, 3H, H-T1), 1.93 – 1.86 (m, 2H, H-3), 1.72 (quint, J = 6.7 Hz, 2H, H-10), 1.47 – 1.09 (m, 48H, H-23, H-S7 and H-4 to H-9). H-M7 to H-M12 could not individually be assigned and are grouped together and abbreviated as H-glycol.

^{13}C{^{1}H}-NMR (151 MHz, CD_2Cl_2, 298 K): δ = 165.4 (C-M19), 160.3 (C-12), 152.9 (C-S4), 152.3 (C-20), 150.8 (C–M13), 150.7 – 150.5 (overlapping signals, $C_{α\text{-pyrrol}}$), 148.2 – 148.1 (overlapping signals, C-M1, C-M6), 147.8 (C-M18), 144.8 (C-2), 143.6 ($C_{ipso\text{-Ph}}$), 139.3 (C-M23), 138.6 (C-M20), 135.6 (C-M22), 135.1 ($C_{o\text{-Ph}}$), 132.6 and 132.5 ($C_{β\text{-pyrrol}}$ and $C_{β\text{-pyrrol'}}$), 132.1 (C-18), 131.1 (C-14), 130.5 (C-S2), 128.4 (C-M16), 128.0 and 127.1 ($C_{m\text{-Ph}}$ and $C_{p\text{-Ph}}$), 126.9 (C-1), 124.7 (C-21), 124.1 – 123.9 (overlapping signals, C-S3, C-S5, C-19), 123.4 (C-15), 122.1 (C-M3, C-M4), 121.5 (C_{meso}), 121.2 (C-M15), 118.8 (C-M21), 114.7 (C-13), 113.2 – 112.9 (overlapping signals, C-M2, C-M5), 112.3 (C-M14), 112.2 (C-M17), 71.7 – 70.3 (several overlapping signals, C-glycol), 69.1 – 68.0 (several overlapping signals, C-glycol and C-11), 58.2 (C-S1), 53.4 (overlapping with solvent signal, assigned by HSQC, C-

17), 53.0 (C-16), 37.2 (C-T1), 35.5 – 35.3 (overlapping signals, C-22, C-S6), 31.7 and 31.5 (C-23 and C-S7), 29.9 – 29.7 and 29.4 – 29.2 (overlapping signals, C-10 and C-8 to C-5), 26.8 (C-4), 26.4 (C-9), 23.2 (C-3).

^{19}F{^{1}H}-NMR (376 MHz, CD_2Cl_2, 298 K): δ = -72.9 (d, *J* = 711.8 Hz, P*F*$_6^-$).

^{31}P{^{1}H}-NMR (162 MHz, CD_2Cl_2, 298 K): δ = -144.5 (sept, *J* = 711.8 Hz, *P*F$_6^-$).

HSQC (400 MHz /101 MHz, CD_2Cl_2, 298 K): δ (^{1}H) / δ (^{13}C) = 9.00/132.6 ($H_{\beta\text{-pyrrol}}$/$C_{\beta\text{-pyrrol}}$), 8.95-8.91/132.5 ($H_{\beta\text{-pyrrol'}}$/$C_{\beta\text{-pyrrol'}}$), 8.26 – 8.16/135.6, 135.1 (H-M22/C-M22 and $H_{o\text{-Ph}}$/$C_{o\text{-Ph}}$), 8.07/118.8 (H-M21/C-M21), 7.83-7.71/128.0, 127.1 ($H_{p\text{-Ph}}$/$C_{p\text{-Ph}}$ and $H_{m\text{-Ph}}$/$C_{m\text{-Ph}}$), 7.57 – 7.48/121.2 (H-M15/C-M15), 7.44/124.1 – 123.9 (H-21/C-21), 7.42/124.7 (H-S5/C-S5), 7.38/112.2 (H-M17/C-M17), 7.33/124.1 – 123.9 (H-19/C-19), 7.22/126.9 (H-1/C-1), 7.09/131.1 (H-14/C-14), 6.99/124.1 – 123.9 (H-S3/C-S3), 6.93 – 6.76/122.1, 113.2 – 112.9, 112.3 (H-M3, H-M4/C-M3, C-M4, H-M2, H-M5/C-M2, C-M5 and H-M14/C-M14), 6.52/114.7 (H-13/C-13), 4.92/58.2 (H-S1/C-S1), 4.76/53.4 (H-17/C-17), 4.54/53.0 (H-16/C-16), 4.32 – 4.22/69.1 – 68.0 (H-glycol/C-glycol), 4.17 – 4.15/69.1 – 68.0 (H-glycol/C-glycol), 3.91 – 3.65/ 71.7 – 70.3, 69.1 – 68.0 (H-glycol/C-glycol and H-11/C-11), 3.60 – 3.43/71.7 – 70.3 (H-glycol/C-glycol), 33.2/37.2 (H-T1/C-T1), 1.93-1.86/23.2 (H-3/C-3), 1.72/29.9 – 29.7 (H-10/C-10), 1.47 – 1.09/31.7, 31.5, 29.9 – 29.7, 29.4 – 29.2, 26.4, 26.8 (H-23, H-S7/ C-23, C-S7 and H-4, H-5, H-6, H-7, H-8, H-9, / C-4, C-5, C-6, C-7, C-8, C-9).

HMBC (400 MHz /101 MHz, CD_2Cl_2, 298 K): δ (^{1}H) / δ (^{13}C) = 9.00/150.7 – 150.5, 132.5 ($H_{\beta\text{-pyrrol}}$/$C_{\alpha\text{-pyrrol}}$, $C_{\beta\text{-pyrrol'}}$), 8.95-8.91/150.7 – 150.5, 132.6 ($H_{\beta\text{-pyrrol}}$'/$C_{\alpha\text{-pyrrol}}$, $C_{\beta\text{-pyrrol}}$), 8.45/118.8 (amide-N*H*/C-M21), 8.26 – 8.16/138.6, 135.1, 128.0, 121.5 ($H_{o\text{-Ph}}$, H-M22/C-M22, $C_{p\text{-Ph}}$, C_{meso}, C-M20, $C_{o\text{-Ph}}$), 8.07/139.3, 118.8 (H-M21/C-M23, C-M21), 7.83 – 7.71/143.6, 135.1, 127.1 ($H_{m\text{-Ph}}$/$C_{ipso\text{-Ph}}$ and $H_{p\text{-Ph}}$/$C_{o\text{-Ph}}$, $C_{m\text{-Ph}}$), 7.57 – 7.48/165.4, 150.8, 112.2 (H-M15/C-M19, C M13, C-M17), 7.44/124.1 – 123.9, 35.5 – 35.3 (H-21/C-19, C-22), 7.41/124.1 – 123.9, 35.5 – 35.3 (H-S5/C-S3, C-S6), 7.38/165.4, 150.8, 147.8, 128.4, 121.2 (H-M17/C-M19, C-M13, C-M18, C-M16, C-M15), 7.33/124.1 – 123.9, 53.4, 35.5 – 35.3 (H-19/C-21, C-17, C-22), 7.22/144.8 (H-1/C-2), 7.09/160.3, 131.1, 53.0 (H-14/C-12, C-14, C16), 6.99/124.1 – 123.9, 58.2, 35.5 – 35.3 (H-S3/C-S3, C-S1, C-S6), 6.93 – 6.76/148.2 – 148.1, 147.8, 128.4, 122.1 (H-M3, H-M4/C-M1, C-M6 and H-M14/C-M18, C-M16 and H-M2, H-M5/C-M4, C-M3), 6.52/160.3, 123.4, 114.7 (H-13/C-12, C-15, C-13), 4.92/130.5, 126.9, 124.1 – 123.9 (H-S1/C-S2, C-1, C-S3), 4.76/132.1, 124.1 – 123.9 (H-17/C-18, C-19), 4.54/131.1, 123.4 (H-16/C-14, C-15), 3.91 – 3.65/71.7 – 70.3, 29.9 – 29.7 , 26.4 (H-glycol/C-glycol, and H-11/C-10, C-9), 3.60 – 3.43/71.7 – 70.3 (H-glycol/C-glycol), 3.32/144.8 (H-T1/C-2), 1.93 – 1.86/144.8, 26.8 (H-3/C-2, C-4), 1.72/69.1 – 68.0 (H-10/C-11), 1.47 – 1.09/152.9, 152.3, 35.5 – 35.3 (H 23/C-20, C-22 and H-S7/C-S4, C-S6).

COSY (400 MHz / 400 MHz, CD_2Cl_2, 298 K): δ = 9.00/8.95-8.91 ($H_{\beta\text{-pyrrol}}$/$H_{\beta\text{-pyrrol'}}$), 8.95-8.91/9.00 ($H_{\beta\text{-pyrrol'}}$/$H_{\beta\text{-pyrrol}}$), 8.26 – 8.16/8.07, 7.83-7.71 ($H_{o\text{-Ph}}$, H-M22/H-M21, $H_{m\text{-Ph}}$, $H_{p\text{-Ph}}$), 8.07/8.26 – 8.16 (H-M21/H-M22), 7.83 – 7.71/8.26 – 8.16 ($H_{m\text{-Ph}}$, $H_{p\text{-Ph}}$ / $H_{o\text{-Ph}}$), 7.57 – 7.48/6.93 – 6.76, 4.76, 4.54 (H-M15/H-M14 and NH_2/H-17, H-16), 7.09/6.52 (H-14/H-13), 6.93 – 6.76/7.57 – 7.48 (H-M14/H-M-15), 6.52/7.09 (H-14/H-13), 4.76/7.57 – 7.48 (H-17/NH_2), 4.54/7.57 – 7.48 (H-16/NH_2), 4.32 – 4.22/4.17 – 4.15, 3.91 – 3.65 (H-glycol/H-glycol, H-glycol), 4.17 – 4.15/4.32 – 4.22, 3.91 – 3.65 (H-glycol/H-glycol, H-glycol), 3.91 – 3.65/4.32 – 4.22, 4.17 – 4.15, 1.72 (H-glycol/H-glycol and H-11/H-10), 3.60 – 3.43/3.91 – 3.65 (H-glycol/H-glycol), 1.93-1.86/1.47 – 1.09 (H-3/H-4), 1.72/3.91 – 3.65 (H-10/H-11), 1.47 – 1.09/1.93-1.86, 1.72 (H-4/H-3 and H-9/H-10).

IR (ATR-FT): $\tilde{\nu}$ = 2953 (m), 2929 (m), 2872 (m), 1598 (w), 1506 (m), 1457 (w), 1441 (w), 1364 (w), 1339 (w), 1319 (w), 1265 (w), 1250 (w), 1203 (w), 1062 (m), 993 (m), 841 (s), 754 (w), 557 (m) cm^{-1}.

MS (ESI-pos, MeOH): m/z = 950.9799 ($[M-2(PF_6)]^{2+}$, calcd. 950.9806 for $[C_{118}H_{135}N_9O_{10}Zn]^{2+}$).

Chromatography (before methylation)

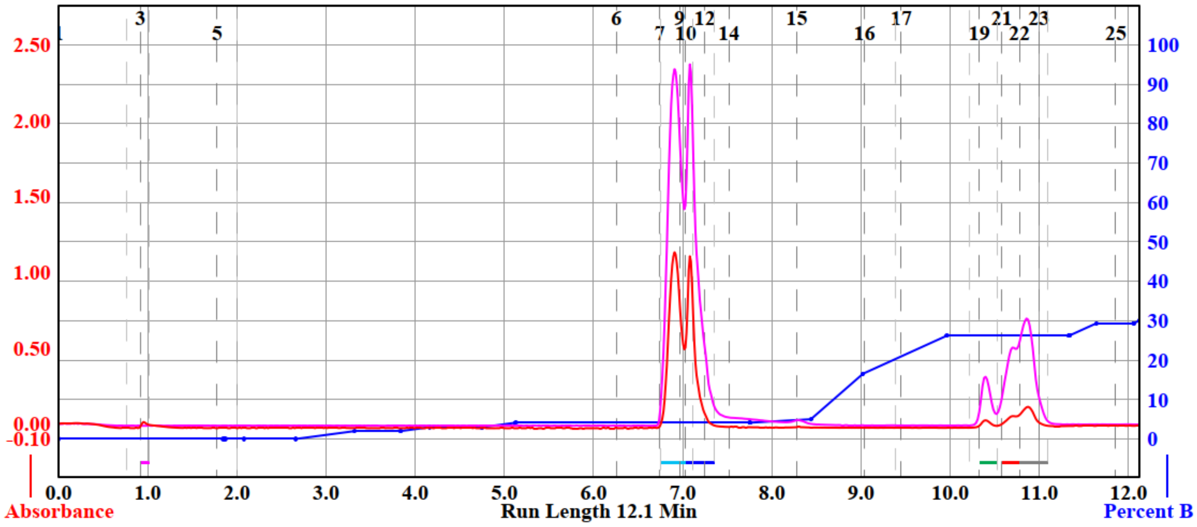

Figure S8: Chromatogram for purification of the unmethylated rotaxane using a gradient of (A) DCM/(B) methanol with a flowrate of 25 mL/min; fractions 8 and 10 were combined after independent NMR analysis of fractions 7 to 12.

1.2.2.8 Synthesis of ZnTPP-Anthracene-rotaxane (**3-H(PF$_6$)$_2$**)

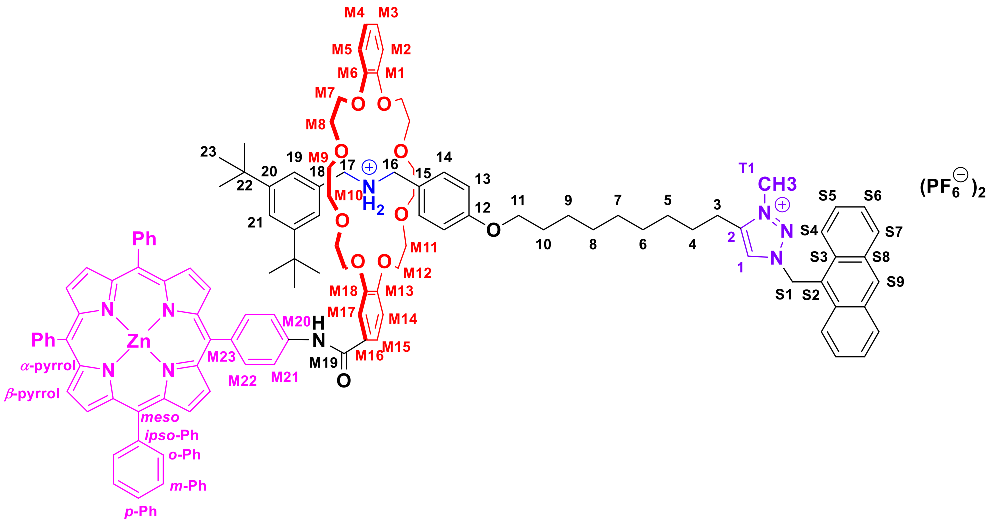

Rotaxane **3** was synthesized according to general procedure A using dibenzylammonium half-thread **4a-HPF$_6$** (20.0 mg, 32.2 µmol, 1 eq.), Zn-porphyrin decorated macrocycle **5** (50.1 mg, 42.9 µmol, 1.2 eq.) and anthracene-azide **S17** (see Figure S3) (9.8 mg, 42 µmol, 1.3 eq.) After purification by column chromatography on a Teledyne Isco NextGen 300+ using a 4g "Chromabond gold" silica column with a dichloromethane/methanol gradient (see Figure S9), the desired product was obtained as a purple glass (37.8 mg, 18.7 µmol, 58.0% yield). The non-methylated rotaxane (13.6 mg, 6.72 µmol) was dissolved in methyl iodide (reaction time 4 days, purification by column chromatography, see Figure S10). After ion exchange, the desired compound was obtained as a purple glass (3.8 mg, 1.7 µmol, 26% yield in respect to unmethylated rotaxane)

$C_{118}H_{123}N_9O_{10}Zn(PF_6)_2$: 2182.6434 g/mol

^{1}H-NMR (400 MHz, CD_2Cl_2, 298 K): δ = 9.03-8.87 (m, 8H, H$_{\beta\text{-pyrrol}}$), 8.37 (s, 1H, amide-N*H*), 8.29 (s, 1H, H-S9), 8.25 – 8.16 (m, 6H, Ho-Ph), 8.13 (d, *J* = 8.3 Hz, 2H, H-M22), 8.04 (d, *J* = 8.3 Hz, 2H, H-M21), 7.90 (d, *J* = 8.9 Hz, 2H, H-S4), 7.84 – 7.71 (m, 11H, H$_{m\text{-Ph}}$, H$_{p\text{-Ph}}$, H-S7), 7.62 – 7.49 (m, 4H, N*H*$_2$ and H-M15 and H$_{\text{Aryl}}$), 7.47 – 7.39 (m, 5H, H-S5 and H-21 and H-M17 and H-1), 7.35 (d, *J* = 1.8 Hz, 2H, H-19), 7.33 – 7.26 (m, 2 H, H-S6), 7.11 (d, *J* = 8.6 Hz, 2H, H-14), 6.96 – 6.75 (m, 6H, H-M2 to H-M5 and H-M14), 6.54 (d, *J* = 8.5 Hz, 2H, H-13), 6.22 (s, 2H, H-S1), 4.77 (ps t, *J* = 6.7 Hz, 2H, H-17), 4.57 (ps t, *J* = 6.5 Hz, 2H, H-16), 4.36 – 4.23 (m, 2H, H-glycol), 4.20 – 4.05 (m, 6H, H-glycol), 3.93 – 3.47 (m, 21H, H-M8, H-M9, H-M10, H-11, H-T1), 2.40 – 2.24 (m, 2H, H-3), 1.70 (quint, *J* = 6.7 Hz, 2H, H-10), 1.35 – 1.15 (m, 30H, H-4 to H9 and H-23; observed integral is 48, likely caused by overlap with residual h-grease). H-M7 to H-M12 could not individually be assigned and are grouped together and abbreviated as H-glycol.

^{13}C{^{1}H}-NMR (101 MHz, CD_2Cl_2, 298 K) δ = 160.2 (not observed in the 1D-spectrum, assigned by HMBC, C-12), 152.3 (C-M20), 150.9 – 150.6 (overlapping signals, C$_{\alpha\text{-pyrrol}}$, C-M13**)**, 148.2 – 147.9 (overlapping signals, C-M1, C-M6, C-M18), 145.4 (C-2), 143.3 (C$_{ipso\text{-}Ph}$), 139.0 (not observed in the 1D-spectrum, assigned by HMBC, C-M23), 138.5 (not observed in the 1D-spectrum, assigned by HMBC, C-M20), 135.5 (C-M22), 135.1 (C$_{o\text{-Ph}}$), 132.7 – 132.5 (overlapping signals, C$_{\beta\text{-pyrrol}}$), 132.1 (C-18),131.7 (C-S9), 131.6 – 131.3 (overlapping signals, C-S3 and C-S8 and C-14), 129.9 (C-S7), 128.9 (C-S5), 128.1 (C$_{p\text{-Ph}}$), 127.2 (C$_{m\text{-Ph}}$), 126.0 (C-S6), 124.1 (overlapping singals, C-19 and C-21, C$_{\text{Aryl}}$), 123.5 (not observed in the 1-D spectrum, assigned by HMBC, C-15), 122.5 (C-S4), 122.1

(overlapping signals, C-M3, C-M4), 121.2 (C-M15), 120.1 (C-S2), 118.8 (C-M21), 114.8 (C-13), 113.1 – 113.0 (overlapping signals, C-M2, C-M5 and C-M14), 112.3 (C-M17), 71.5 – 70.4 and 69.5 – 68.1 (overlapping signals, C-M7 to C-M12 and C-11), 53.4 (overlapping with solvent signal, assigned by HSQC, C-17), 53.0 (C-16), 50.2 (C-S1), 37.8 (C-T1), 35.4 (C-22), 31.7 (C-23), 30.4 – 28.9 (overlapping signals, C-4 to C-10), 23.5 (C-3).

C-M19, C-M16, C_{meso} and C-1 were not observed.

$^{19}F\{^1H\}$-NMR (376 MHz, CD_2Cl_2, 298 K): δ = -73.1 (d, *J* = 710.8 Hz, PF_6^-).

$^{31}P\{^1H\}$-NMR (162 MHz, CD_2Cl_2, 298 K): δ = -144.6 (sept, *J* = 711.3 Hz, PF_6^-)

HSQC (400 MHz /101 MHz, CD_2Cl_2, 298 K): δ (^{1}H) / δ (^{13}C) = 9.03-8.87/132.7 – 132.5 ($H_{\beta\text{-pyrrol}}$/$C_{\beta\text{-pyrrol}}$), 8.29/131.7 (H-S9/C-S9), 8.25 – 8.16/135.1 ($H_{o\text{-Ph}}$/$C_{o\text{-Ph}}$), 8.13/135.5 (H-M22/C-M22), 8.04/118.8 (H-M21/C-M21), 7.90/122.5 (H-S4/C-S4), 7.84 – 7.71/129.9, 128.1, 127.2 (H-S7/C-S7, $H_{p\text{-Ph}}$/$C_{p\text{-Ph}}$, $H_{m\text{-Ph}}$/$C_{m\text{-Ph}}$), 7.62 – 7.49/124.1, 121.2 (H_{Aryl}/C_{Aryl}, H-M15/C-M15), 7.47 – 7.39/128.9, 124.1, 112.3 (H-S5/C-S5, H-21/C-21, H-M17/C-M17), 7.35/124.1 (H-19/C-19), 7.33 – 7.26/126.0 (H-S6/C-S6), 7.11/131.6 – 131.3 (H-14/C-14), 6.96 – 6.75/122.1, 113.1 – 113.0 (H-M3, H-M4/C-M3, C-M4 and H-M2, H-M5/C-M2, C-M5 and H-M14/C-M14), 6.54/114.8 (H-13/C-13), 6.22/50.2 (H-S1/C-S1), .77/53.4 (H-17/C-17), 4.57/53.0 (H-16/C-16), 4.36 – 4.23/69.5 – 68.1 (H-glycol/C-glycol), 4.20/4.05/71.5 – 70.4, 69.5 – 68.1 (H-glycol/C-glycol and H-glycol/C-glycol), 3.93 – 3.64 /71.5 – 70.4, 69.5 – 68.1, 37.8 (H-glycol/C-glycol and H-11/C-11 and H-T1/C-T1), 2.40 – 2.24/23.5 (H-3/C-3), 1.70/30.4 – 28.9 (H-10/C-10), 1.35 – 1.15/31.7, 30.4 – 28.9, (H-23/C-23 and H-5 to H-9/C-5 to C-9).

HMBC (400 MHz /101 MHz, CD_2Cl_2, 298 K): δ (^{1}H) / δ (^{13}C) = 9.03-8.87/150.9 – 150.6 ($H_{\beta\text{-pyrrol}}$/$C_{\alpha\text{-pyrrol}}$), 8.29/131.6 – 131.3, 129.9 (H-S9/C-S8, C-S7), 8.25 – 8.16/135.1, 128.1 ($H_{o\text{-Ph}}$/ $C_{o\text{-Ph}}$, $C_{p\text{-Ph}}$), 8.13/138.5 (H-M22/C-M20), 8.04/139.0 (H-M21/C-M23), 7.90/131.6 – 131.3, 126.0 (H-S4/C-S3, C-S6), 7.84 – 7.71/143.3, 131.6 – 131.3, 128.9, 127.2 ($C_{m\text{-Ph}}$/$C_{ipso\text{-Ph}}$ and H-S7/C-S3, C-S5 and $H_{m\text{-Ph}}$/$C_{m\text{-Ph}}$), 7.47 – 7.39/150.9 – 150.6, 145.4, 124.2 (H-M17/C-M13 and H-1/C-2 and H-21/C-19), 7.35/124.1, 53.4, 35.4 (H-19/C-21, C-17, C-22), 7.33 – 7.26/131.6 – 131.3 (H-S6/C-S8), 7.11/160.2, 131.6 – 131.3 (H-14/C-12, C-14), 6.96 – 6.75/148.2 – 147.9, 122.1 (H-M3, H-M4/C-M1, C-M6 and H-14/C-M18, H-17), 6.54/123.5 (H-13/C-15), 6.22/131.6 – 131.3, 120.1 (H S1/C-S3, C-S2), 3.93 – 3.64/145.4 (H-T1/C-2), 2.40 – 2.24/145.5 (H-3/C-2), 1.35 – 1.15/152.3, 35.4, 31.7 (H-23/C-20, C-22, C-23).

COSY (400 MHz /400 MHz, CD_2Cl_2, 298 K): δ (^{1}H) / δ (^{1}H) = 8.25 – 8.16/7.84 – 7.71 ($H_{o\text{-Ph}}$/$H_{m\text{-Ph}}$), 8.13/8.04 (H-M22/H-M21), 8.04/8.13 (H-M21/H-M22), 7.90/7.47 – 7.39 (H-S4/H-S5), 7.84 – 7.71/8.25 – 8.16, 7.90, 7.33 – 7.26 ($H_{m\text{-Ph}}$/$H_{o\text{-Ph}}$ and H-S7/H-S4, H-S6), 7.62 – 7.49/6.96 – 6.75, 4.77, 4.56 (H-M15/H-M14 and N*H*$_2$/H-17, H-16), 7.47 – 7.39/7.90, 7.35, 7.33 – 7.26 (H-S5/H-S4, H-S6 and H-21/H-19), 7.35/7.47 – 7.39 (H-19/H-21), 7.33 – 7.26/7.84 – 7.71, 7.47 – 7.39 (H-S6/H-S7, H-S5), 7.11/6.54 (H-14/H-13), 6.96 – 6.75/7.62 – 7.49 (H-M14/H-M15), 6.54/7.11 (H-13/H-14), 4.77/7.62 – 7.49 (H-17/N*H*$_2$), 4.57/7.62 – 7.49 (H-17/N*H*$_2$), 4.36 – 4.23/4.20 – 4.05, 3.93 – 3.47 (H-glycol/H-glycol, H-glycol), 4.20 – 4.05/4.36 – 4.23, 3.93 – 3.64 (H-glycol/H-glycol and H-glycol/H-glycol and H-glycol/H-glycol), 3.93 – 3.64/4.36 – 4.23, 4.20 – 4.05, 1.70 (H-glycol/H-glycol and H-glycol/H-glycol and H-M12/H-M11 and H-11/H-10), 2.40 – 2.24/1.35 – 1.15 (H-3/H-4), 1.70/3.93 – 3.47 (H-10/H-11), 1.35 – 1.15/2.40 – 2.24 (H-4/H-3).

IR (ATR-FT): $\tilde{\nu}$ = 2953 (m), 2929 (w), 2872 (w), 1598 (w), 1506 (m), 1457 (w), 1441 (w), 1364 (w), 1339 (w), 1320 (w), 1250 (m), 1203 (w), 1062 (m), 993 (m), 925 (w), 841 (s), 797 (w), 754 (w), 719 (w), 704 (w), 557 (m) cm^{-1}.

MS (ESI-pos, MeOH): m/z = 944.9337 ([M-2(PF_6)]$^{2+}$, calcd. 944.9337 for [$C_{118}H_{123}N_9O_{10}Zn$]$^{2+}$).

Chromatography (before methylation)

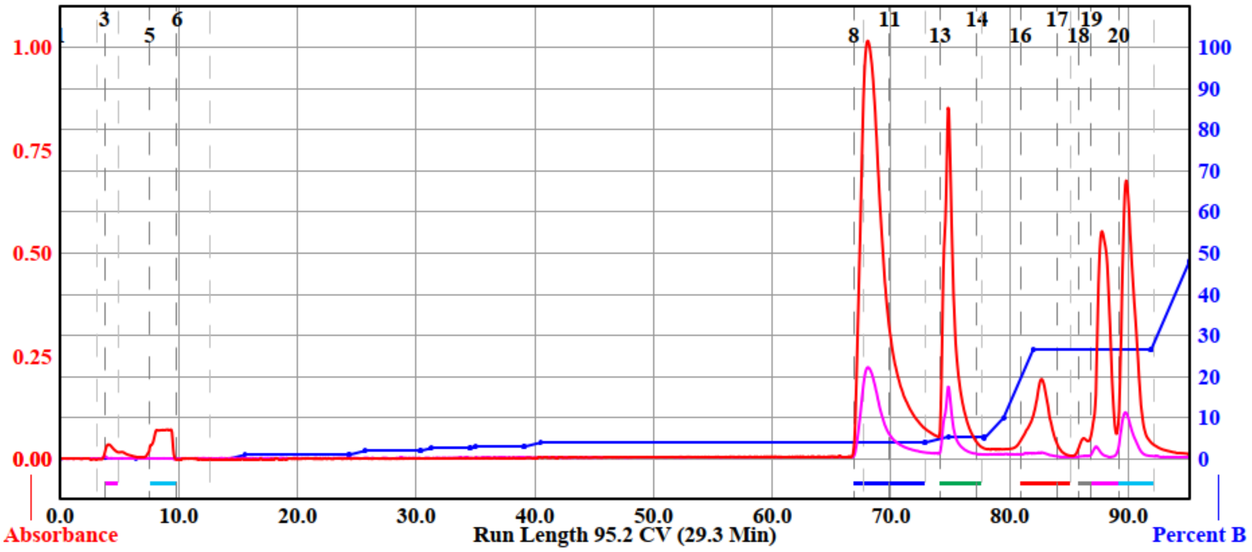

Figure S9: Chromatogram for purification of the unmethylated rotaxane using a gradient of (A) DCM/(B) methanol with a flowrate of 20 mL/min; fractions 8 to 12 were combined.

Chromatography (after methylation)

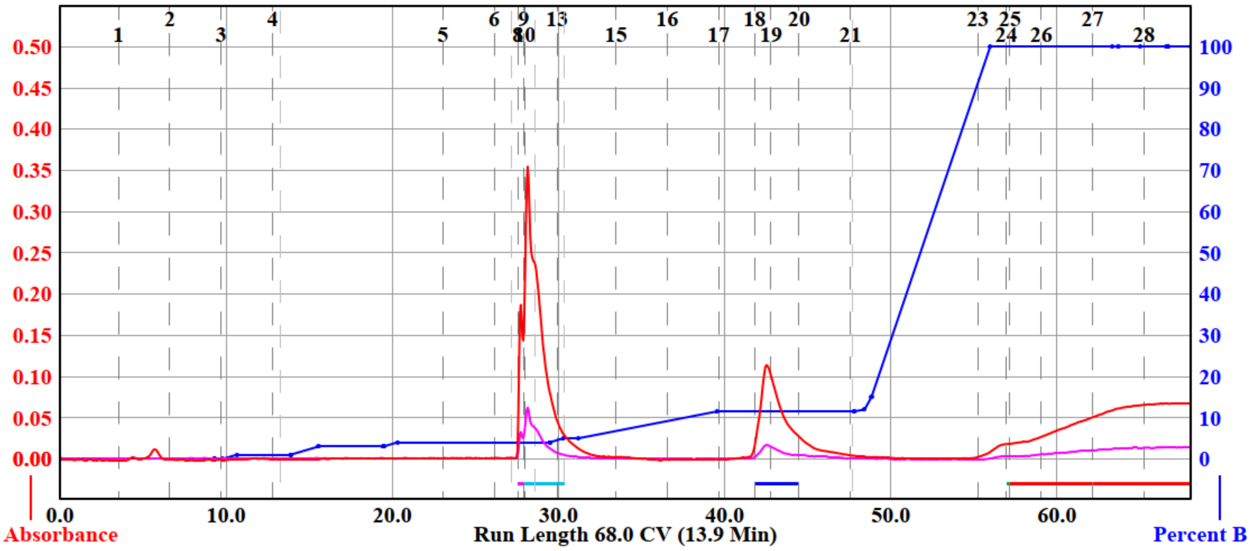

Figure S10: Chromatogram for purification of rotaxane **H-3**$^{2+}$ after methylation using a gradient of (A) DCM/(B) methanol with a flowrate of 30 mL/min; fractions 11 to 14 were combined.

1.2.2.9 Anthracene-dummy-rotaxane (**S20-H(PF₆)₂**)

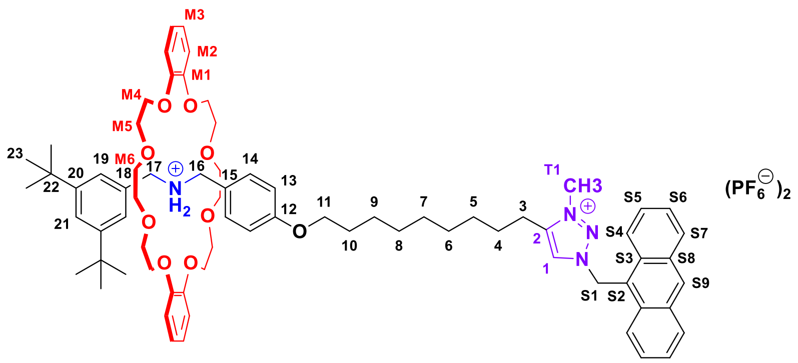

Rotaxane **S20** was synthesized according to general procedure A using dibenzylammonium **4a-HPF$_6$** (30.0 mg, 48.3 µmol mmol, 1 eq.), dibenzo-24-crown-8 (26.0 mg, 58.0 µmol, 1.2 eq.) and anthracene-azide **S17** (see Figure S3) (14.6 mg, 62,6 µmol, 1.3 eq.). After purification by column chromatography on a Teledyne Isco NextGen 300+ using a 4g "Chromabond gold" silica column with a dichloromethane/methanol gradient (see Figure S11) the desired product was obtained as a yellow oil (45.8 mg, 0.035 mmol, 72.8% yield). The obtained rotaxane (40.0 mg, 30.7 µmol) was methylated (reaction time 6 days, no further purification) and after ion exchange, the desired methylated rotaxane (44.9 mg 30.7 µmol, quantitative) was obtained as an orange solid.

$C_{73}H_{96}N_4O_9(PF_6)_2$: 1463,5184 g/mol

^{1}H-NMR (600 MHz, CD_2Cl_2, 298 K): δ = 8.71 (s, 1H, H-S9), 8.30 (dd, *J* = 8.9, 1.0 Hz, 2H, H-S4), 8.15 (dd, *J* = 8.2 Hz, 1.2, 2H, H-S7), 7.86 (s, 1H, H-1), 7.72 (ddd, *J* = 9.0, 6.6, 1.3 Hz, 2H, H-S5), 7.60 (ddd, *J* = 8.4, 6.6, 1.0 Hz, 2H, H-S6), 7.51 (br s, 2H, NH_2), 7.39 (t, *J* = 1.7 Hz, 1H, H-21), 7.29 (d, *J* = 1.7 Hz, 2H, H-19), 7.08 (d, *J* = 8.7 Hz, 2H, H-14), 6.95 – 6.75 (m, 8H, H-M3 and H-M2), 6.72 (s, 2H, H-S1), 6.52 (d, *J* = 8.7 Hz, 2H, H-13), 4.72 (ps t, *J* = 6.7 Hz, 2H, H-17), 4.50 (ps t, *J* = 6.7 Hz, 2H, H-16), 4.14 (s, 3H, H-T1), 4.12 – 4.07 (m, 8H, H-M4), 3.85 – 3.68 (m, 10H, H-M5 and H-11), 3.65 – 3.42 (m, 8H, H-M6), 2.70 (t, *J* = 8.3 Hz, 2H, H-3), 1.69 (q, *J* = 6.7 Hz, 2H, H-10), 1.61 (q, *J* = 8.1 Hz, 2H, H-4), 1.44 – 1.16 (m, 28H, H-5 to H-9 and H-23).

^{13}C{^{1}H}-NMR (151 MHz, CD_2Cl_2, 298 K): δ = 160.1 (C-12), 152.1 (C-20), 147.9 (C-M1), 145.6 (C-2), 131.9 – 131.8 (overlapping signals, C-S9 and C-S8), 131.6 (C-S3), 131.1 (C-14), 130.1 (C-S7), 130.0 (C-S5), 127.6 (C-1), 126.1 (C-S6), 124.0 (C-19), 123.8 (C-21), 123.3 (C-15), 122.9 (C-S4), 122.0 (C-M3), 120.5 (C-S2), 114.6 (C-13), 113.0 (C-M2), 71.0 (C-M6), 70.6 (C-M5), 68.4 (C-M4), 68.3 (C-11), 53.2 (C-17), 52.8 (C-16), 50.5 (C-S1), 38.1 (C-T1), 35.2 (C-22), 31.5 (C-23), 30.1 – 29.2 (overlapping signals, C-10 and C-8 to C-5), 27.1 (C-4), 26.2 (C-9), 23.8 (C-3). C-18 could not be assigned.

^{19}F{^{1}H}-NMR (376 MHz, CD_2Cl_2, 298 K): δ = -73.4 (d, *J* = 711.5 Hz, P*F*$_6^-$).

^{31}P{^{1}H}-NMR (162 MHz, CD_2Cl_2, 298 K): δ = -144.6 (sept, *J* = 711.5 Hz, *P*F$_6^-$).

HSQC (600 MHz /151 MHz, CD_2Cl_2, 298 K): δ (^{1}H) / δ (^{13}C) = 8.71/131.9 – 131.8 (H-S9/C-S9), 8.30/122.9 (H-S4/C-S4), 8.15/130.1 (H-S7/C-S7), 7.86/127.6 (H-1/C-1), 7.72/130.0 (H-S5/C-S5), 7.60/126.1 (H-S6/C-S6), 7.39/123.8 (H-21/C-21), 7.29/124.0 (H-19/C-19), 7.08/131.1 (H-14/C-14), 6.95 – 6.75/122.0, 113.0 (H-M3/C-M3 and H-M2/C-M2), 6.72/50.5 (H-S1/C-S1), 6.52/114.6 (H-13/C-13), 4.72/53.2 (H-17/C-17), 4.50/52.8 (H-16/C-16), 4.14/38.1 (H-T1/C-T1), 4.12 – 4.07/68.4 (H-M4/C-M4), 3.85 – 3.68/70.6, 68.3 (H-M5/C-M5 and H-11/C-11),

3.65 – 3.42/71.0 (H-M6/C-M6), 2.70/23.8 (H-3/C-3), 1.69/30.1 – 29.2 (H-10/C-10), 1.61/27.1 (H-4/C-4), 1.44 – 1.16/31.5, 30.1 – 29.2, 26.2 (H-23/C-23 and H-5 to H-9/C-5 to C-8).

HMBC (600 MHz /151 MHz, CD_2Cl_2, 298 K): δ (^{1}H) / δ (^{13}C) = 8.71/131.9 – 131.8, 130.1 (C-S9/C-S8, C-S7), 8.30/131.9 – 131.8, 126.1, 120.5 (H-S4/C-S8, C-S6, C-S2), 8.15/131.9 – 131.8, 130.0 (H-S7/C-S9, C-S5), 7.86/145.6 (H-1/C-2), 7.72/131.6, 130.1 (H-S5/C-S3, C-S7), 7.60/131.9 – 131.8, 122.9 (H-S6/C-S8, C-S4), 7.39/124.0, 35.2 (H-21/ C-19, C-22), 7.29/123.8, 53.2, 35.2 (H-19/C-21, C-17, C-22), 7.08/160.1, 131.1, 52.8 (H-14/C-12, C-14, C-16), 6.95 – 6.75/147.9, 122.0, 113.0 (H-M3 and H-M2/C-M1, C-M3, C-M2), 6.72/131.6, 127.6, 120.5 (C-S1/C-S3, C-1, C-S2), 6.52/123.3, 114.6 (H-13/C-15, C-13), 4.50/131.1 (H-16/C-14), 4.14/145.6 (H-T1/C-2), 3.85 – 3.68/30.1 – 29.2, 26.2 (H-11/H-10, H-9), 2.70/145.6, 127.6, 27.1 (H-3/C-2, C-1, C-4), 1.44 – 1.16/152.1, 35.2 (H-23/C-20, C-22).

COSY (600 MHz /600 MHz, CD_2Cl_2, 298 K): δ (^{1}H) / δ (^{1}H) = 8.30/7.72 (H-S4/H-S5), 8.15/7.60 (H-S7/H-S6), 7.72/8.30, 7.60 (H-S5/H-S4, H-S6), 7.60/8.15,7.72 (H-S6/H-S7, H-S5), 7.51/4.72, 4.50 (NH_2/H-17, H-16), 7.39/7.29 (H-21/H-19), 7.29/7.39 (H-19/H-21), 7.08/6.52 (H-14/H-13), 6.52/7.08 (H-13/H-14), 4.72/7.51 (H-17/NH_2), 4.50/7.51 (H-16/NH_2), 4.12 – 4.07/3.85 – 3.68 (H-M4/H-M5), 3.85 – 3.68/4.12 – 4.07, 1.69 (H-M5/H-M4 and H-11/H-10), 2.70/1.61 (H-3/H-4), 1.69/3.85 – 3.68, 1.44 – 1.16 (H-10/H-11, H-9), 1.61/2.70, 1.44 – 1.16 (H-4/H-3, H-5), 1.44 – 1.16/1.69, 1.61 (H-9/H-10 and H-5/H-4).

IR (ATR-FT): $\tilde{\nu}$ = 2953 (m), 2922 (m), 2871 (w), 1593 (w), 1505 (w), 1457 (w), 1251 (m), 1214 (w), 1123 (w), 1108 (w), 1057 (w), 954 (w), 840 (s), 742 (w), 558 (m) cm^{-1}.

MS (ESI-pos, MeOH): m/z = 586.3578 ([M-(2PF_6)]$^{2+}$, calcd. 586.3583 for [$C_{73}H_{96}N_4O_9$]$^{2+}$).

Chromatography (before methylation)

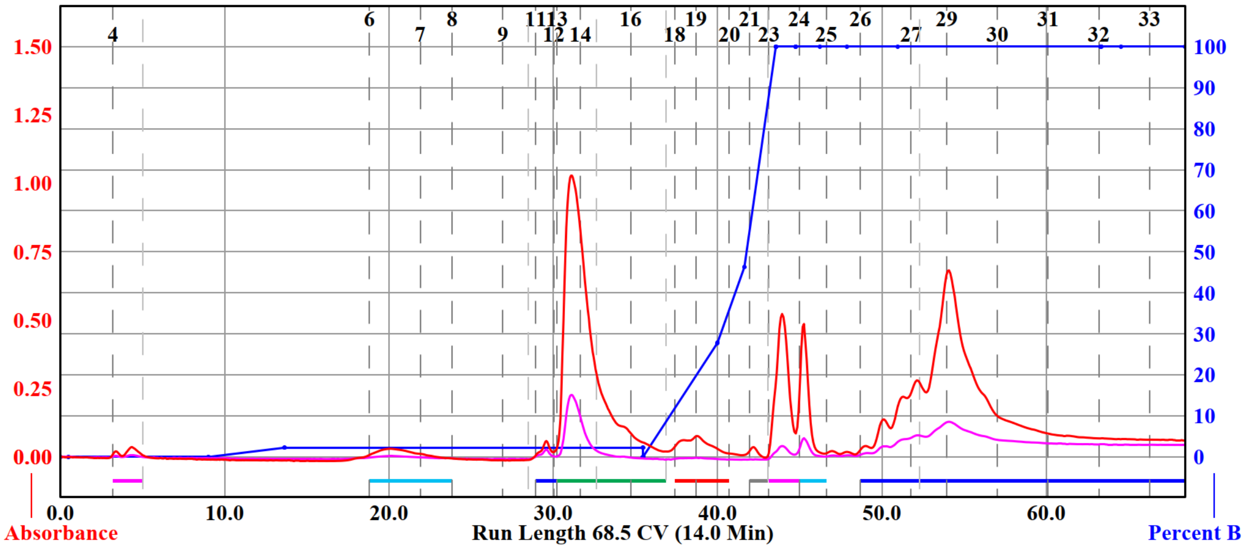

Figure S11: Chromatogram for purification of the rotaxane before methylation using a gradient of (A) DCM/(B) methanol with a flowrate of 30 mL/min; fractions 13 to 18 were combined.

1.2.2.10 General procedure B: Deprotonation of switchable rotaxanes

The protonated rotaxane-bishexafluorophosphate was dissolved in acetone (2 mL/10 mg) and 50 mM aqueous sodium hydroxide (2 eq.) was added. Volatiles were removed *in vacuo* and the residue was taken up in dichloromethane and filtered over a polyamide syringe filter. The filtrate was evaporated to yield the deprotonated molecule.

1.2.2.11 Deprotonated ZnTPP-BHQ-2 rotaxane **1a-PF6**

Rotaxane **1a-H(PF$_6$)$_2$** (26.4 mg, 10.8 µmol) was deprotonated according to general procedure B. Deprotonated rotaxane **1a-PF$_6$** was obtained as a purple solid (24.8 mg, 10.8 µmol, quantitative yield).

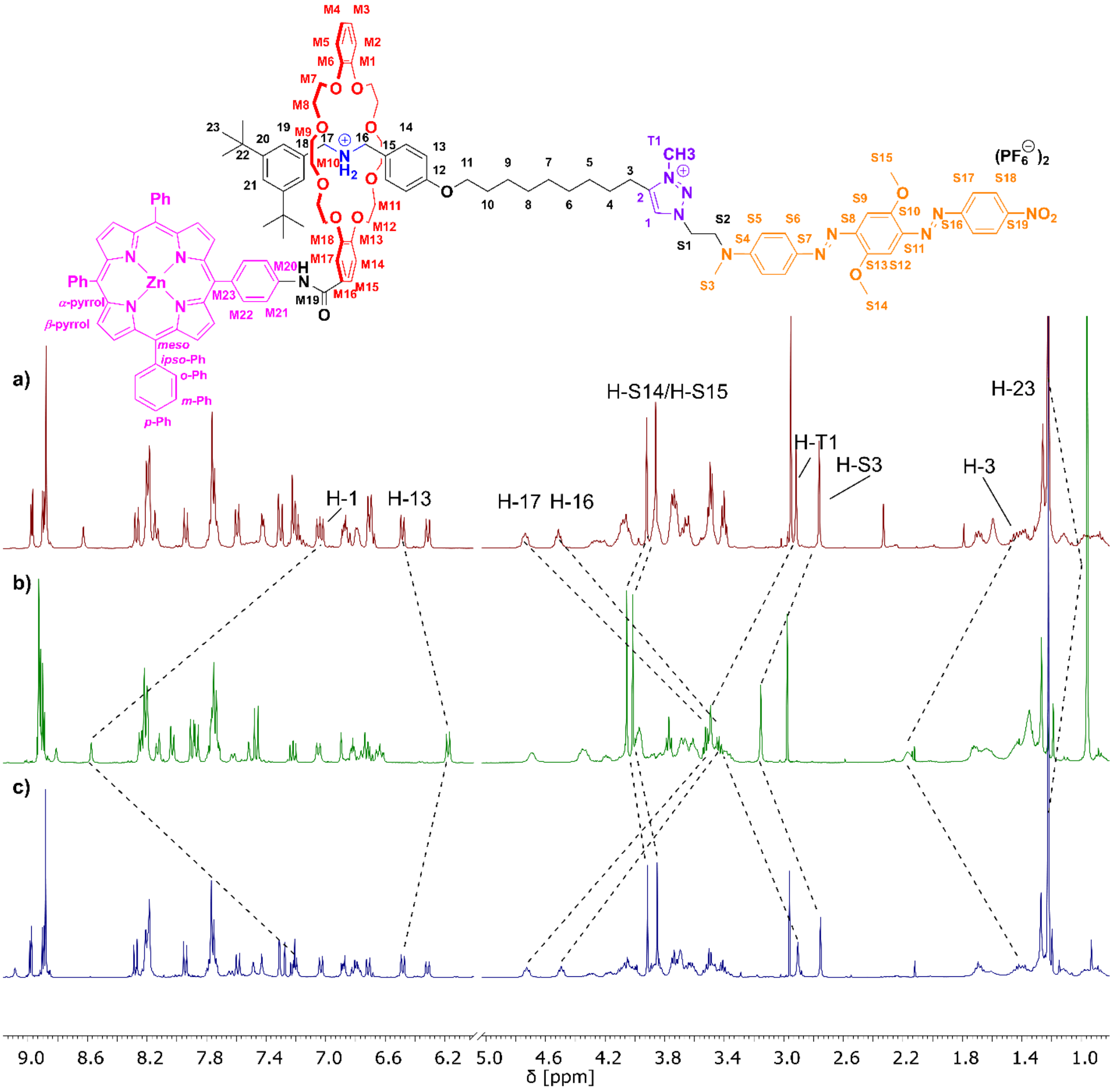

Figure S12: Partial ^{1}H-NMR spectra of a) protonated rotaxane **1a-H(PF$_6$)$_2$**; b) deprotonated rotaxane **1a-PF$_6$**; c) the sample of deprotonated rotaxane after addition of 1 equivalent CF_3COOH. All 400 MHz, CD_2Cl_2, 298 K. Numbering scheme is different from the one in Fig. 2 in the main article and instead is the same as used in this SI.

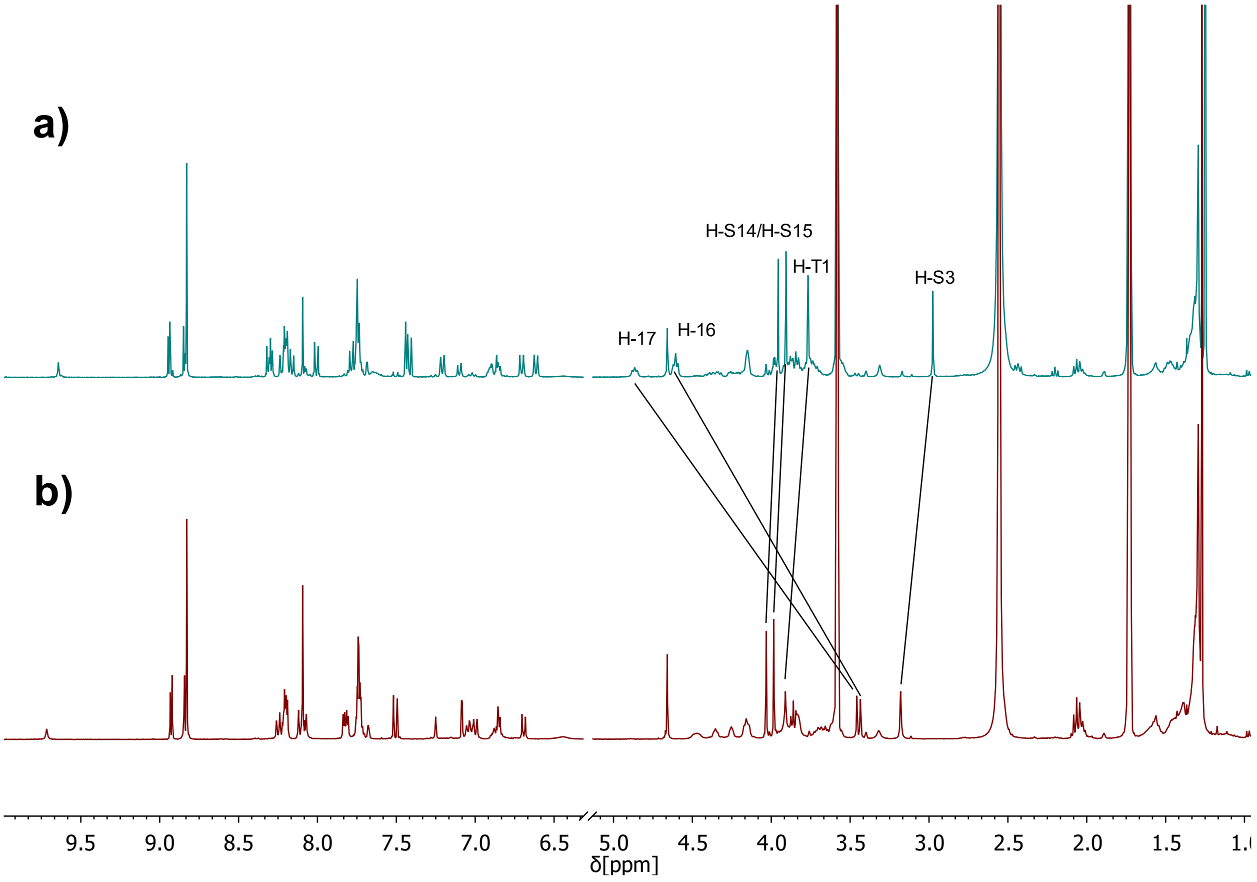

Figure S13: Stacked partial ^{1}H-NMR spectra of a) protonated rotaxane **1a-H(PF$_6$)$_2$**; b) deprotonated rotaxane **1a-PF$_6$**; All 600 MHz, THF-d$_8$, 298 K.

1.2.2.12 Deprotonated short ZnTPP-BHQ-2-rotaxane **1b-PF₆**

Rotaxane **1b-H(PF$_6$)$_2$** (6.5 mg, 2.76 µmol) was deprotonated according to general procedure B. The deprotonated rotaxane **1b-PF6** was obtained as a purple solid (4.4 mg, 1.99 µmol, 72.2% yield).

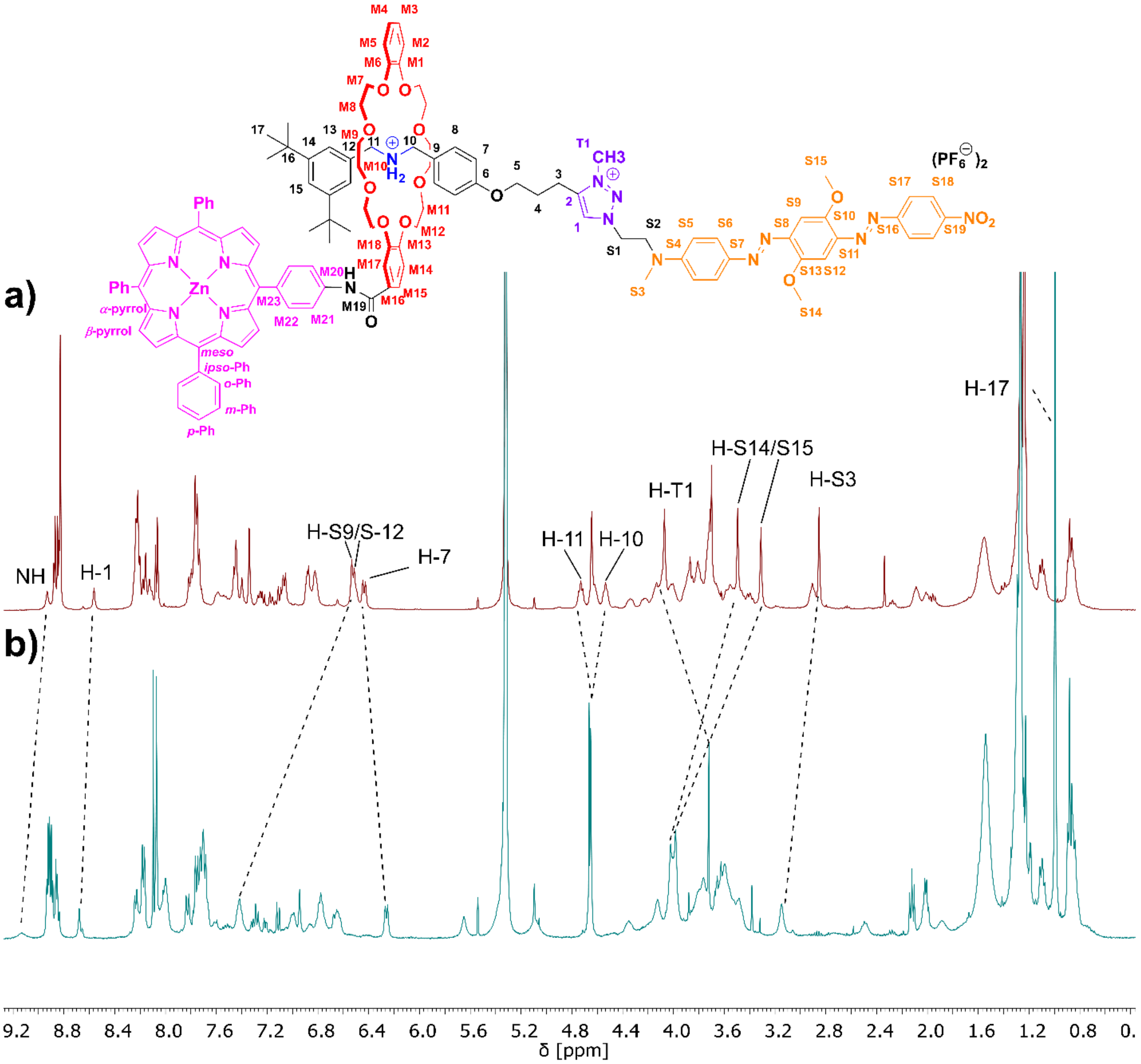

Figure S14: Stacked ^{1}H-NMR spectra of a) protonated rotaxane **1b-H(PF$_6$)$_2$**; b) **1b-PF$_6$** after deprotonation. All 400 MHz, CD_2Cl_2, 298 K.

1.2.2.13 Deprotonated Dummy-Rotaxane **2-PF6**

Rotaxane **2-H(PF6)2** (7.6 mg, 3.5 µmol) was deprotonated according to general procedure B. The deprotonated rotaxane **2-PF6** was obtained as a purple solid (7.1 mg, 3.5 µmol, quantitative yield).

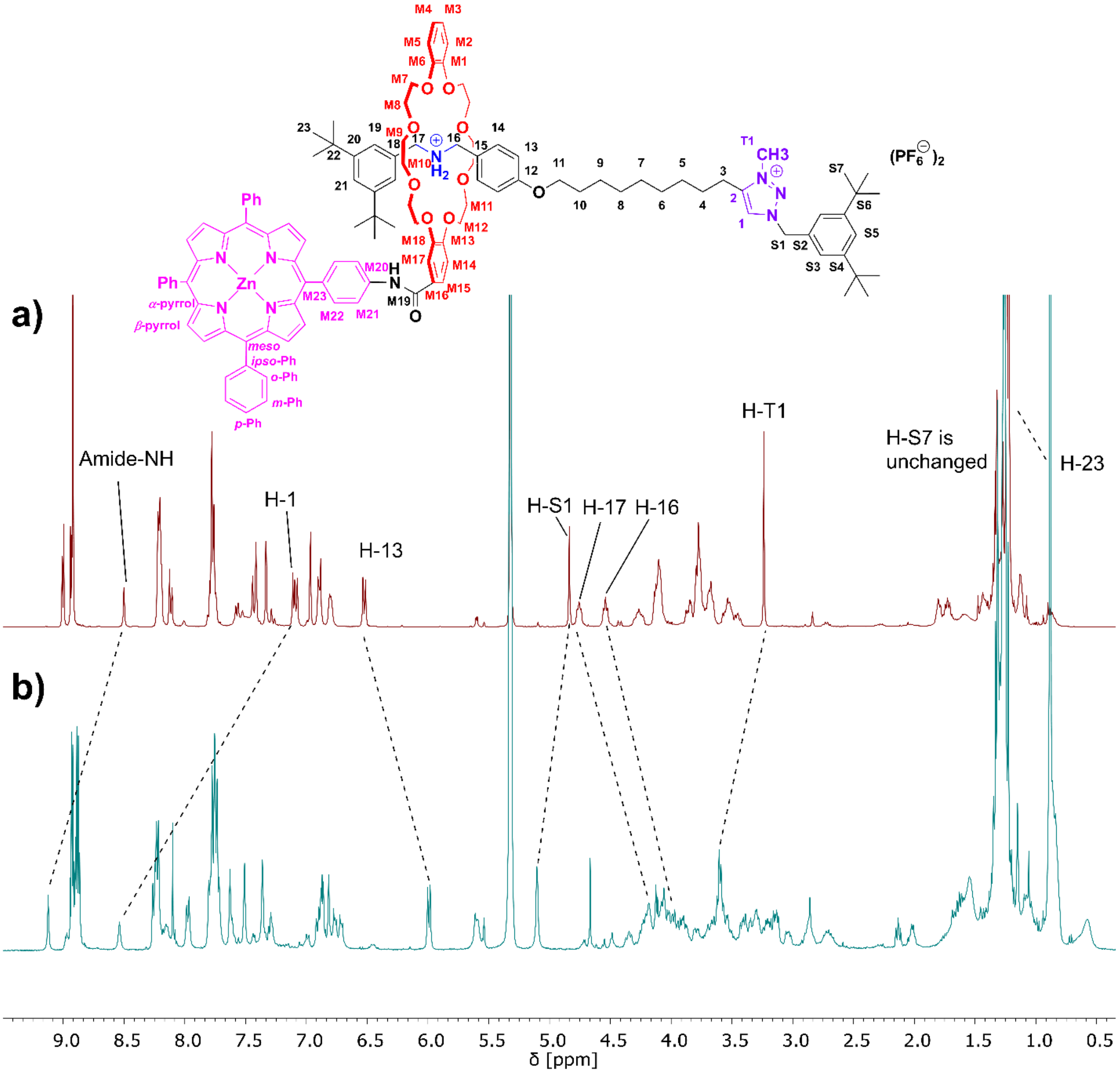

Figure S15: Stacked ^{1}H-NMR spectra of a) protonated rotaxane **2-H(PF6)2**; b) **2-PF6** after deprotonation. All 400 MHz, CD_2Cl_2, 298 K.

1.2.2.14 Deprotonated ZnTPP-Anthracene-rotaxane **3-PF6**

Rotaxane **3-H(PF6)2** (2.3 mg, 1.1 µmol) was deprotonated according to general procedure B. The deprotonated rotaxane **3-PF6** was obtained as a purple solid (2.1 mg, 1.1 µmol, quantitative yield).

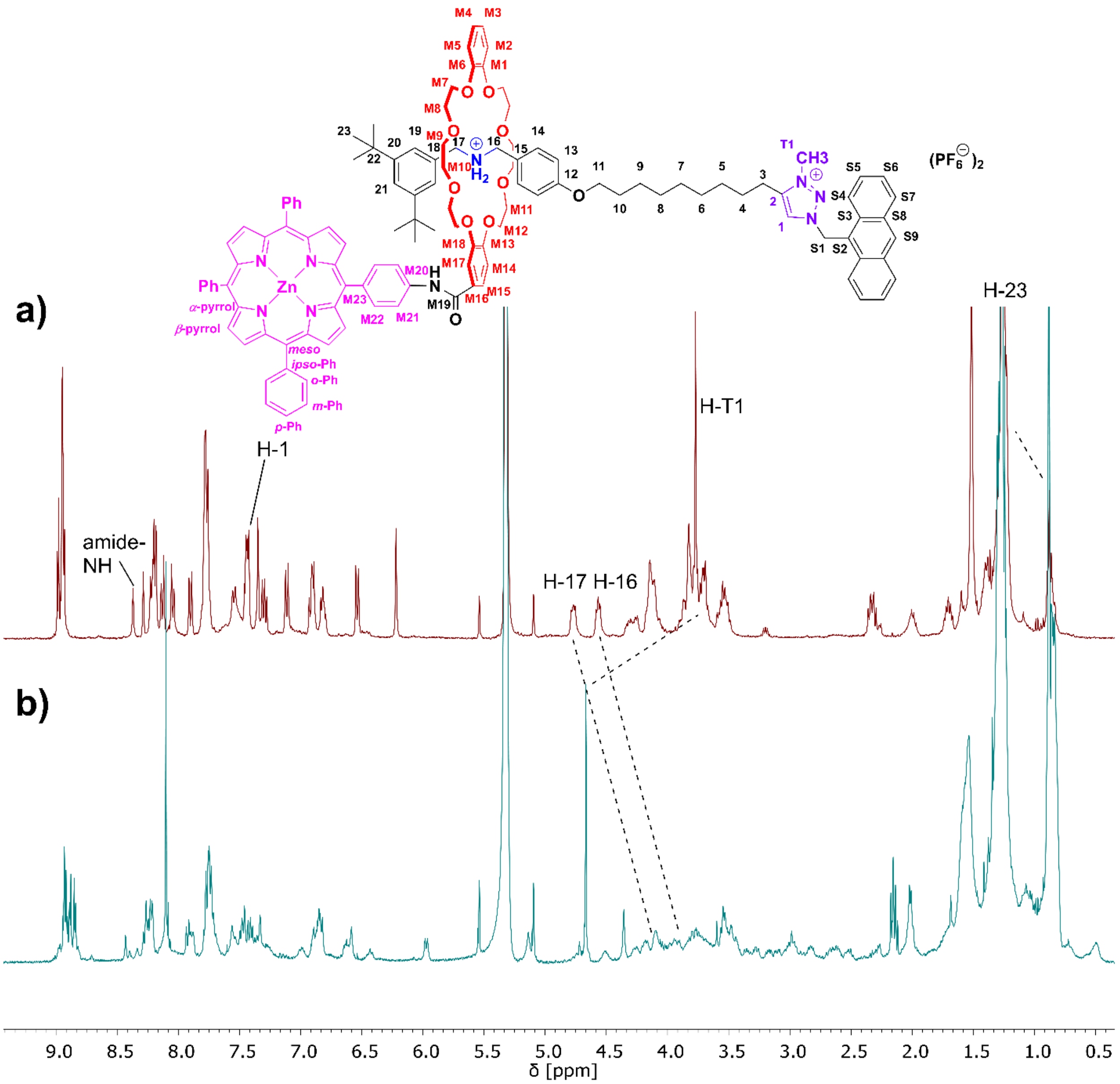

Figure S16: Stacked ^{1}H-NMR spectra of a) protonated rotaxane **3-H(PF6)2**; b) **3-PF6** after deprotonation. All 400 MHz, CD_2Cl_2, 298 K.

1.2.2.15 Deprotonated Anthracene-Dummy rotaxane **S20-PF$_6$**

Rotaxane anthracene-dummy **S20-H(PF$_6$)$_2$** (17.0 mg, 11.6 µmol) was deprotonated according to general procedure B. The deprotonated rotaxane (15.3 mg, 11.6 µmol, quantitative yield) was obtained as a pale yellow waxy solid.

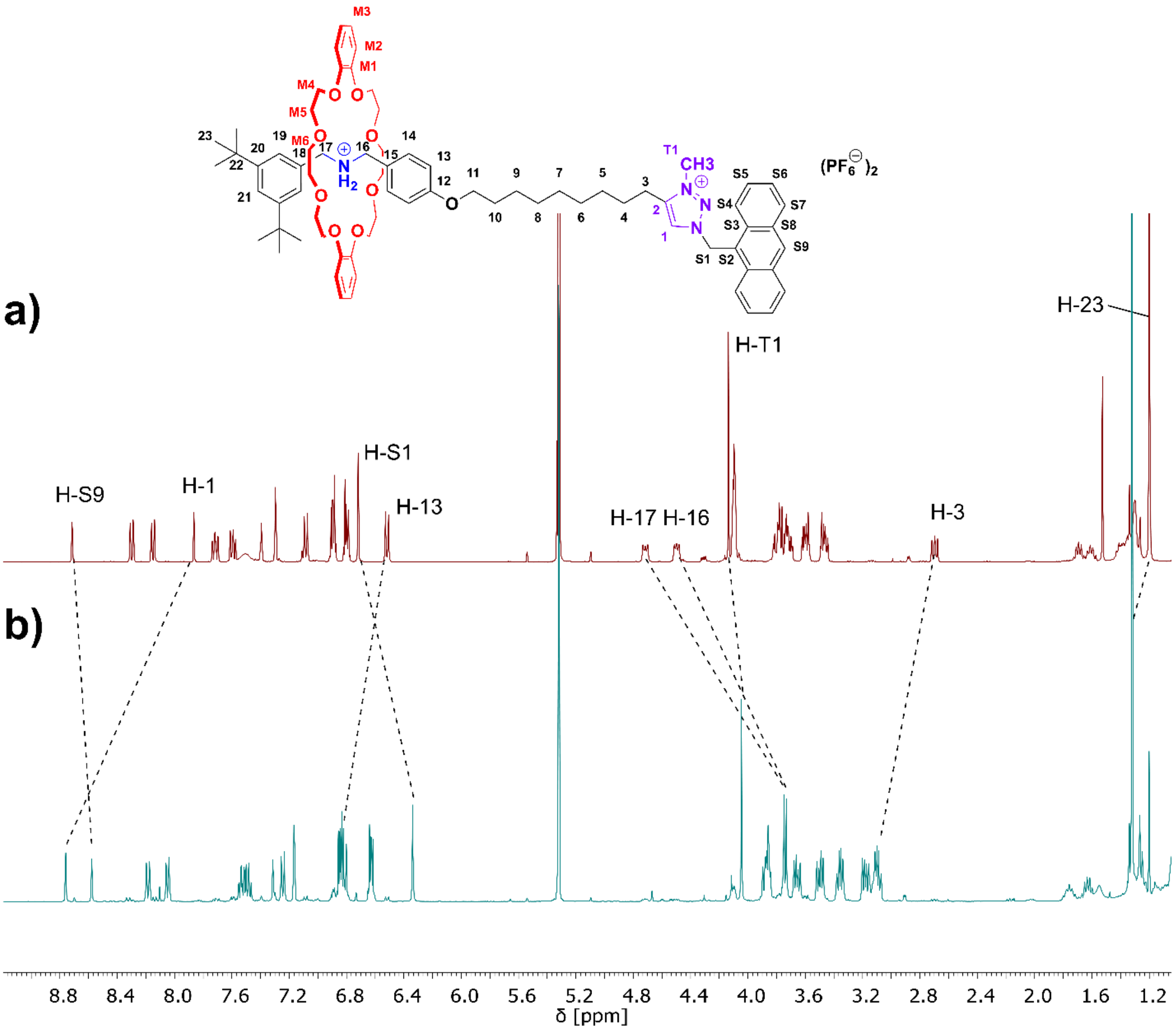

Figure S17: Stacked ^{1}H-NMR spectra of a) protonated rotaxane **S20-H(PF$_6$)$_2$**; b) **S20-PF$_6$** after deprotonation. All 400 MHz, CD_2Cl_2, 298 K.

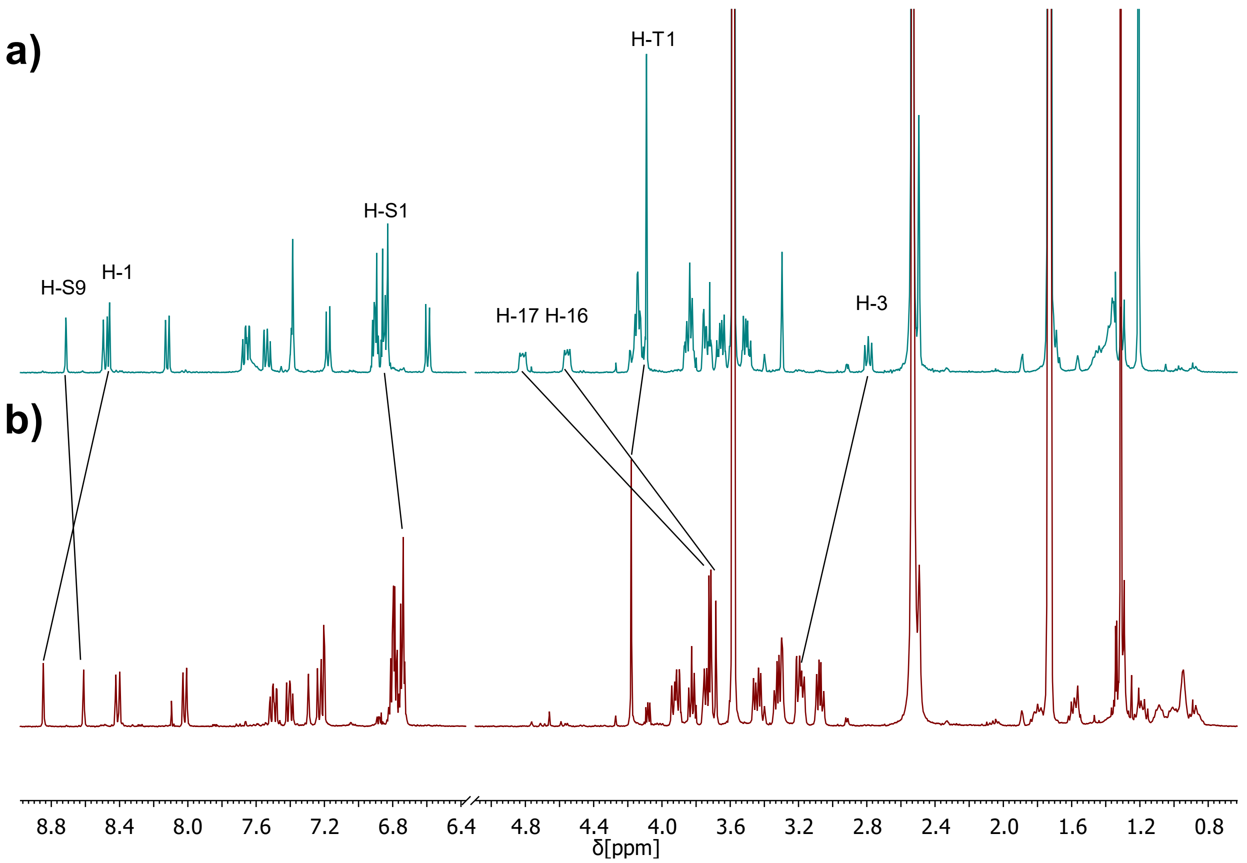

Figure S18: Stacked partial ^{1}H-NMR spectra of a) protonated rotaxane **S20-H(PF$_6$)$_2$**; b) **S20-PF$_6$** after deprotonation. All 400 MHz, THF-d$_8$, 298 K.

1.3 Pseudorotaxanes between ammonium half-threads and crown-ether

When half-thread **4a-HPF$_6$** is combined with crown-ether **5** in $CDCl_3$, the formation of the pseudorotaxane host-guest complex can be directly observed and the association constant K_a can be calculated, since the equilibrium is in the slow exchange regime, thus showing distinct signals for all three components in the mixture, which can be integrated to find the relative amounts of the species (e.g. I_{HG}, I_H and I_G) (see Figure S19).

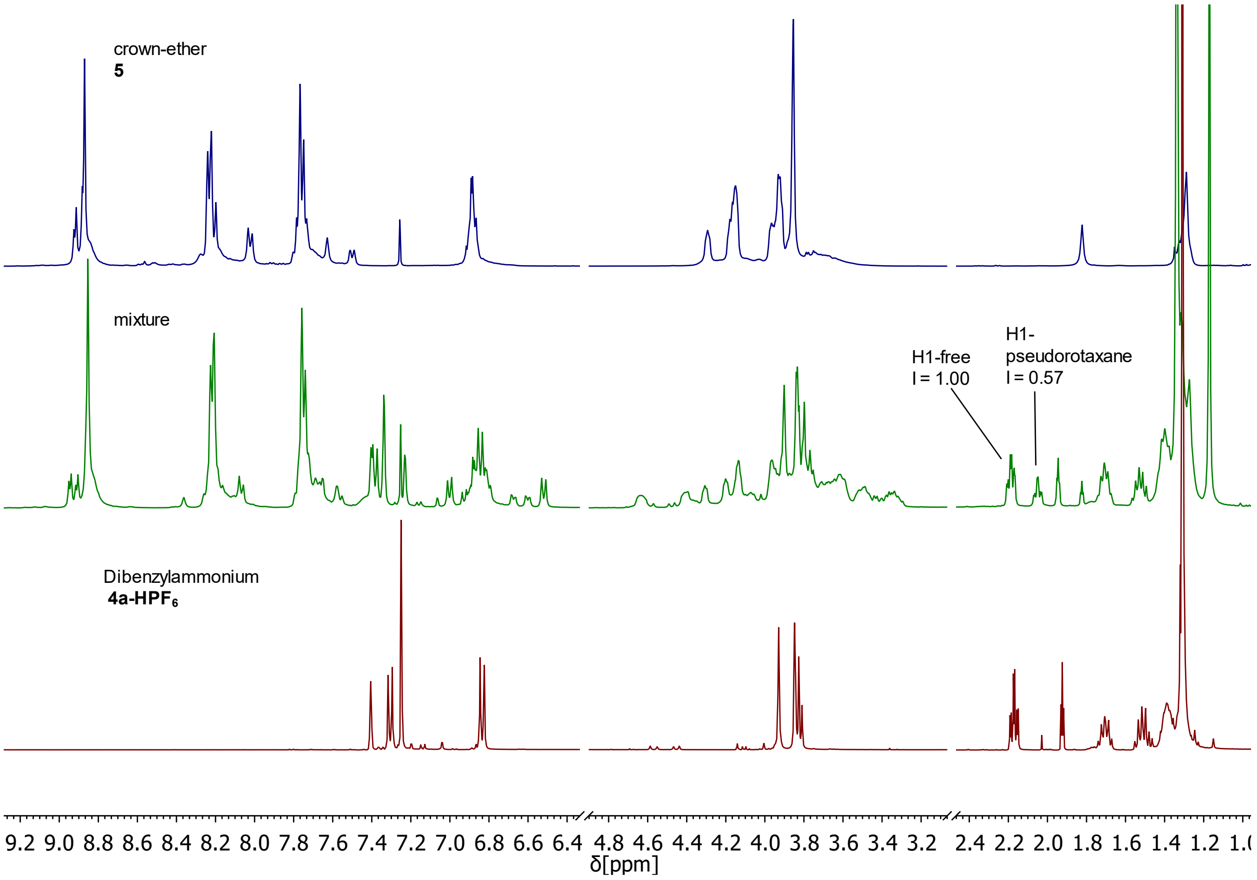

Figure S19: Stacked partial ^{1}H-NMR spectra of (top) crown-ether **5**, (bottom) dibenzylammonium half-thread **4a-HPF$_6$**, and (middle) a mixture of “host” **5** and “guest” **4a-HPF$_6$** at initial concentrations of 24 mM and 20 mM, respectively (all 400 MHz, $CDCl_3$, 298 K).

Very similar behavior is observed for a mixture of half-thread **4b-HPF$_6$** and the same macrocycle (see Figure S20). In this case almost all other signals for the pseudorotaxane and free components overlap, so the signals corresponding to the *tert*-butyl groups on the thread were used for integration, since their chemical shifts differ significantly and they are well separated.

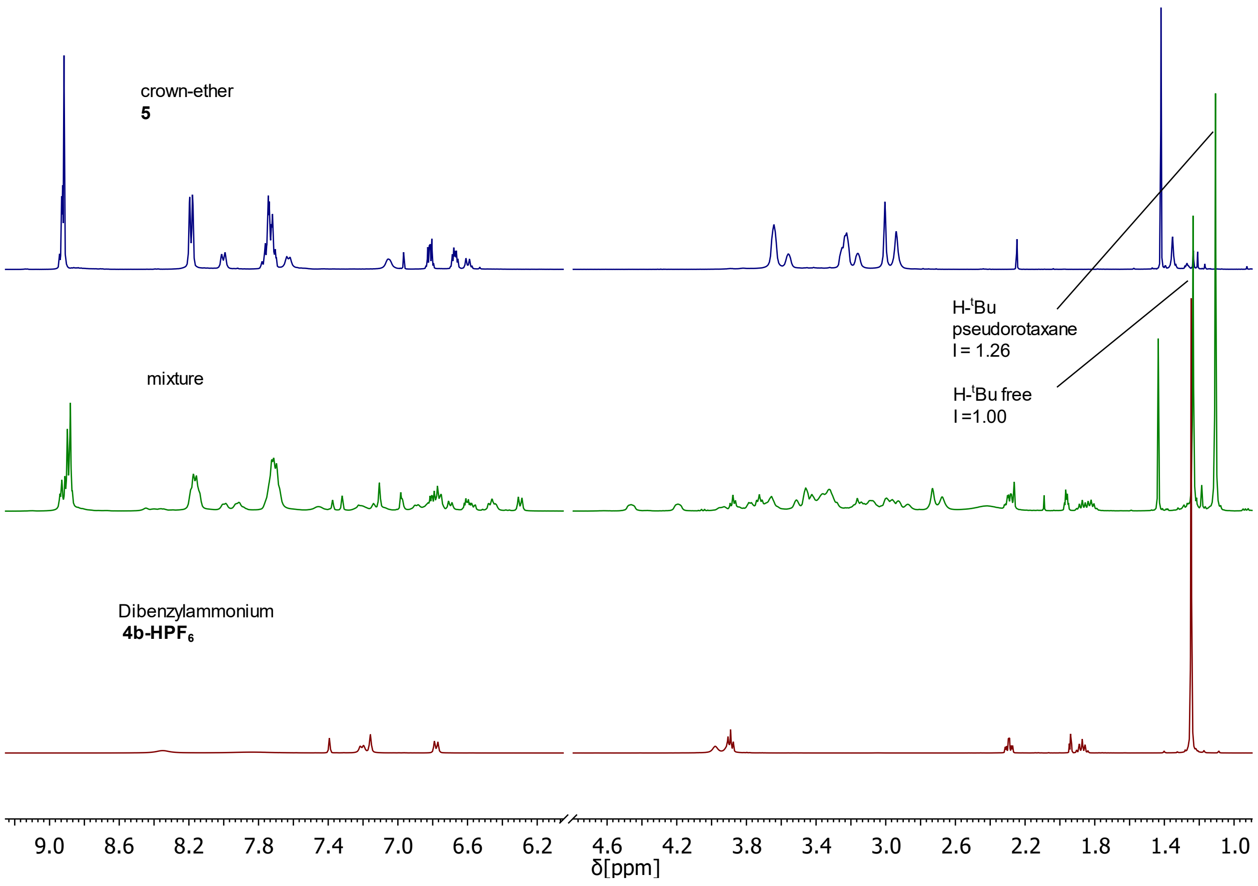

Figure S20: Stacked partial ^{1}H-NMR spectra of (top) crown-ether **5**, (bottom) dibenzylammonium half-thread **4b-HPF₆**, and (middle) a mixture of "host" **5** and "guest" **4b-HPF₆** at initial concentrations of 41.3 mM and 33.8 mM, respectively (all 400 MHz, CD_2Cl_2, 298 K).

The association constant can be calculated from the following equations:

$$X_{HG} = \frac{I_{HG}}{I_{HG} + I_H} \tag{S1}$$

$$R = \frac{[G_0]}{[H_0]} \tag{S2}$$

$$K_a = \frac{X_{HG}/[H_0]}{(1 - X_{HG})(R - X_{HG})} \tag{S3}$$

H = **5**, G = **4a/b-HPF₆**, HG = pseudorotaxane

The calculated value for the pseudorotaxane with half-thread **4a-HPF₆** is K_a = 61 L mol^{-1} ($CDCl_3$) and for half-thread **4b-HPF₆** K_a = 143 L mol^{-1} (CD_2Cl_2).

1.4 Photophysical characterization and measurements of singlet oxygen production

1.4.1 Measurements of singlet oxygen production with DPBF

A solution of diphenylisobenzofuran (DPBF) in THF was prepared (concentration 860 µM) in an amber glass volumetric flask. The stock solution was stored in the freezer, protected from light, and absorbance was checked before use. Portions were diluted as needed.

Approximately 10 µM solutions of free porphyrin or the rotaxanes were prepared in THF under ambient conditions and the absorbance of the most intense band (*Soret*-band around 424 nm) was adjusted to 0.8. Then 70 µL DPBF-solution (860 µM) was added in the dark. Then, the cuvette was irradiated at intervals of 10 or 30 seconds and fluorescence emission spectra (λ_{Exc} = 420 nm) were recorded to monitor the decrease in fluorescence intensity (*I*) of DPBF at 457 nm over time.

For irradiation, a halogen cold light source "VisiLight CL-150" (VWR) equipped with a 150 W "64620 EFR 5" (OSRAM) bulb and Shimadzu cut-on filters were used. The light irradiation intensity was measured with a power meter "Solarmeter 9.6" (Solartech) and adjusted to 2.0 mW/cm^2 using a Shimadzu R-60 600 nm filter. The distance to the lamp was kept constant and the filter was exchanged for a L-42 420 nm filter for irridiation.

Calculation of singlet oxygen quantum yields was performed by the following equation:[19]

$$\Phi_{\Delta}(s) = \Phi_{\Delta}(ref)\,\frac{r_s \cdot (1 - 10^{-Abs(ref)})}{r_{ref} \cdot (1 - 10^{-Abs(s)})} \qquad \text{(S4)}$$

Here *r* is the slope of a linear fit of the semilogarithmic plot of ln(I_0/I) versus time after substraction of the slope of the background reaction, obtained by irradiating a sample containing no photosensitizer, plotted in the same way (see Figure S22). H_2TPP (freebase tetraphenylporphyrin) (Φ_{Δ} = 0.62) was used as a first reference to determine the quantum yield of ZnTPP (zinc(II) tetrpahenylporphyrin), which was verified using the direct measurement of singlet oxygen phosphorescence (see chapter 1.4.3). Since the measured singlet oxygen quantum yields were in good agreement and also in line with literature values, ZnTPP was then used as secondary reference for the rotaxane systems. For these calculations Φ_{Δ}(ref) = 0.72 for ZnTPP was used. The rotaxanes were measured as triplicates and the reported values are the mean with the errors representing the standard deviation of the three measurements (see Figure S21).

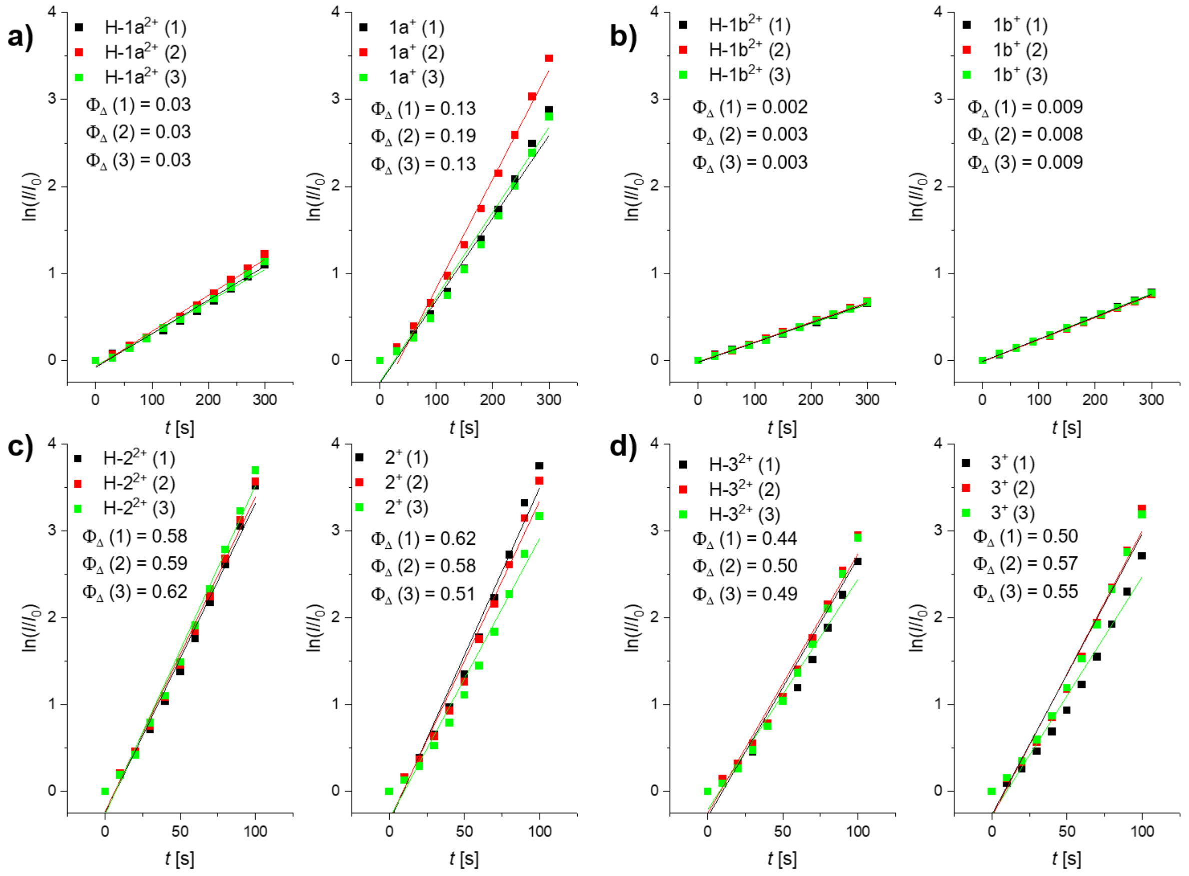

Figure S21: Triplicates for semilogarithmic plot of ln(I_0/I) of DPBF emission versus irradiation time for a) rotaxane **H-1a**$^{2+}$ and **1a**$^{+}$, b) rotaxane **H-1b**$^{2+}$ and **1b**$^{+}$, c) rotaxane **H-2**$^{2+}$ and **2**$^{+}$, d) rotaxane **H-3**$^{2+}$ and **3**$^{+}$. Solid lines are linear least square fits. Singlet oxygen quantum yields are calculated for each measurement according to equation S4.

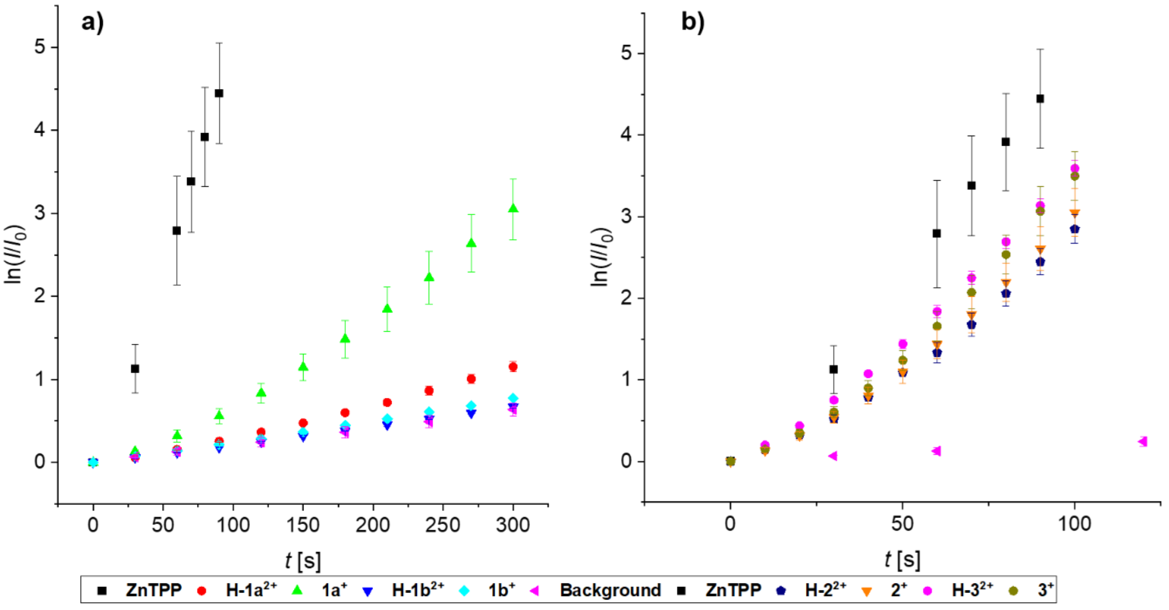

Figure S22: Mean of triplicates with uncertainty bars (standard deviation) without substraction of the background for a) rotaxanes **1a/b** in both protonation states and **ZnTPP** and b) control rotaxanes **2** and **3** in both protonation states and **ZnTPP**. Uncertainty bars for some measurments are too small to observe.

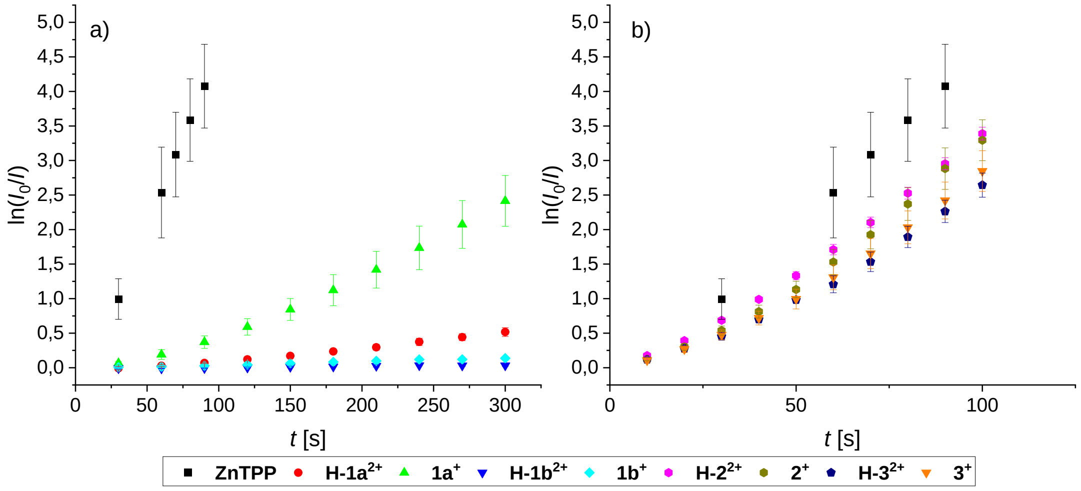

Figure S23: Mean of triplicates with uncertainty bars (standard deviation) after substraction of the background for a) rotaxanes **1a/b** in both protonation states and **ZnTPP** and b) control rotaxanes **2** and **3** in both protonation states and **ZnTPP**. Uncertainty bars for some measurments are too small to observe.

1.4.2 Förster radius of the ZnTPP/BHQ-2-pair

The Förster radius R_0 of a donor-acceptor pair can be calculated according to the following equation:[20]

$$R_0 = 0.02108\,(\kappa^2\,\Phi_\mathrm{F}\,n^{-4}\,J)^{1/6} \tag{S5}$$

In absence of a better value, the orientation factor κ^2 is approximated as 0.67; n is the refractive index of THF (n = 1.4073) and Φ_F is the fluorescence quantum yield of the donor **ZnTPP** (Φ_F = 0.03). The spectral overlap integral J was determined by the following equation from the molar absorptivity spectrum of BHQ-2-azide (**6**) and the luminescence emission spectrum of **ZnTPP** in THF:

$$J = \int \varepsilon_A(\lambda)\, I_D(\lambda)\, \lambda^4\, d\lambda \tag{S6}$$

Here ε_A is the molar absorption coefficient of the acceptor (**6**) and I_D is the donor (**ZnTPP**) emission normalized to unity. The utilized spectra are shown in Figure S24.

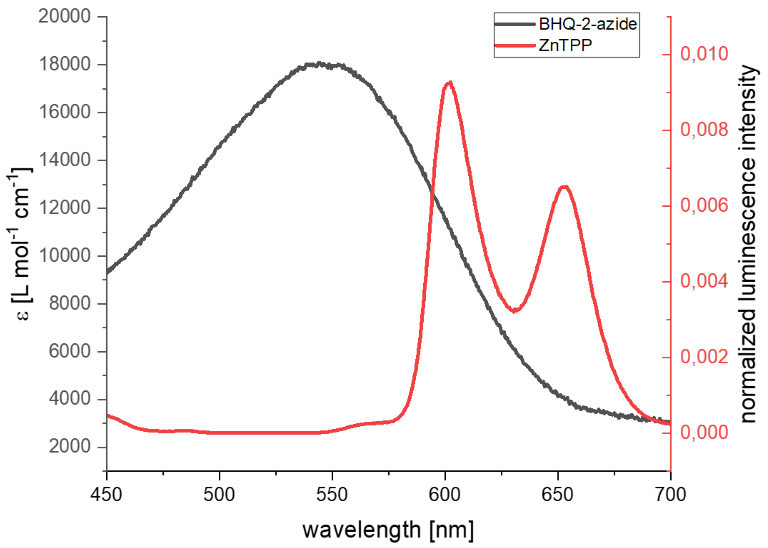

.

Figure S24: Overlap of the absorption spectrum of **6** (molar absorption coefficients, black) and the area-normalized emission spectrum of **ZnTPP** (red) in THF.

The value found was J = 1.05·10^{15} nm^4·mol^{-1}·cm^{-1}. Combining all the obtained values in equation S5 gives a Förster-radius of R_0 = 2.8 nm. This value however is only an estimate, since we have found significant interaction between the ZnTPP and the BHQ-2-moiety (*vide infra*), leading to a somewhat more rigid system, which has an influence on the value of κ^2. Earlier research has shown that κ^2=0.67 can still serve as a useful estimate resulting in errors for R_0 of ± 20%, which is still sufficient for our purpose.[21],[22] For example, assuming κ^2 = 0.5 results in R_0 = 2.7 nm and for κ^2 = 1 we find R_0 = 3.0 nm, both within ± 20% of the original estimate.

1.4.3 Photophysical characterization and singlet oxygen phosphorescence

Steady-state excitation and emission spectra were recorded on a FluoTime 300 spectrometer from PicoQuant equipped with: a 300 W ozone-free Xe lamp (250-900 nm), a 10 W Xe flash-lamp (250-900 nm, pulse width *ca*. 1 µs) with repetition rates of 0.1 – 300 Hz, a double-grating excitation monochromator (Czerny-Turner type, grating with 1200 lines/mm, blaze wavelength: 300 nm), diode lasers (pulse width < 80 ps) operated by a computer-controlled laser driver PDL-828 “Sepia II” (repetition rate up to 80 MHz, burst mode for slow and weak decays), two double-grating emission monochromators (Czerny-Turner, selectable gratings blazed at 500 nm with 2.7 nm/mm dispersion and 1200 lines/mm, or blazed at 1200 nm with 5.4 nm/mm dispersion and 600 lines/mm) with adjustable slit width between 25 µm and 7 mm, Glan-Thompson polarizers for excitation (after the Xe-lamps) and emission (after the sample). Different sample holders (Peltier-cooled mounting unit ranging from -15 to 110 °C or an adjustable front-face sample holder), along with two detectors (namely a PMA Hybrid-07 from PicoQuant with

transit time spread FWHM < 50 ps, 200 – 850 nm, or a H10330C-45-C3 NIR detector with transit time spread FWHM 0.4 ns, 950-1700 nm from Hamamatsu) were used. Steady-state spectra and photoluminescence lifetimes were recorded in TCSPC mode by a PicoHarp 300 (minimum base resolution 4 ps) or in MCS mode by a TimeHarp 260 (where up to several ms can be traced). Emission and excitation spectra were corrected for source intensity (lamp and grating) by standard correction curves. For samples with lifetimes in the ns order, an instrument response function calibration (IRF) was performed using a diluted Ludox® dispersion. Lifetime analysis was performed using the commercial EasyTau 2 software (PicoQuant). The quality of the fit was assessed by minimizing the reduced chi squared function (χ^2) and visual inspection of the weighted residuals and their autocorrelation.

Absorbance measurements for singlet oxygen quantification were measured with a Shimadzu UV-3600 I plus UV-VIS-NIR spectrophotometer. Additionally, the photogeneration of $^1O_2$ was determined by steady-state infrared emission spectroscopy. Photosensitized singlet oxygen phosphorescence intensity was monitored as a function of the fraction of light absorbed ($1–10^{-A}$) and Φ_Δ values were obtained with equation S7:

$$\Phi_\Delta^{\mathrm{S}} = \Phi_\Delta^{\mathrm{R}} \frac{m^{\mathrm{S}}}{m^{\mathrm{R}}} \tag{S7}$$

Here *S* stand for the compound of interest, *R* stands for a reference photosensitizer with a known Φ_Δ value, and *m* is the slope of the integrated emission versus the fraction of light absorbed. H_2TPP was used as the reference in deuterated tetrahydrofuran (THF-d_8) (Φ_Δ = 0.62)[23] and the intensities were corrected for the intensity of the excitation beams at each wavelength. For all measurements, matched quartz (Hellma®) cuvettes were used.

All solvents used were of spectrometric grade (Uvasol, Merck).

For the laser flash photolysis measurements, a LP980-K spectrometer (Edinburgh Instruments) equipped with a tunable wavelength nanosecond laser (EKSPLA Nd:YAG laser, model NT-342), a TGM325-A monochromator (325 mm focal length monochromator in Czerny Turner configuration), a 150 W CW Xenon lamp and a L-D07 PMT-980 (LP) Photomultiplier Assembly were used. The sensitivity of the LP980-K (minimum detectable optical density) is ΔOD = 0.002, as specified by the manufacturer. The laser pulse-width is 3-5 ns and the instrument response function (IRF) width is 5 ns, as specified by the manufacturers. Data were detected and subsequently converted using a Digital Storage Oscilloscope (Tektronix MDO3022 Mixed Domain Oscilloscope) for transient recording (sampling speed 2.5 GS/s, analogue bandwidth: 200 MHz, 10M record length, 2 channels). In addition, two different internal energy meters (L-U04F special internal energy meters) were placed in the equipment before and after the sample. In this way, is possible to measure the energy of each excitation pulse (with the power meter before the sample) and the energy transmitted by the sample (with the power meter after the sample). Data were collected and subsequently analyzed with the L900 spectrometer software (Edinburgh Instruments) and MatLab R2016 (MathWorks).

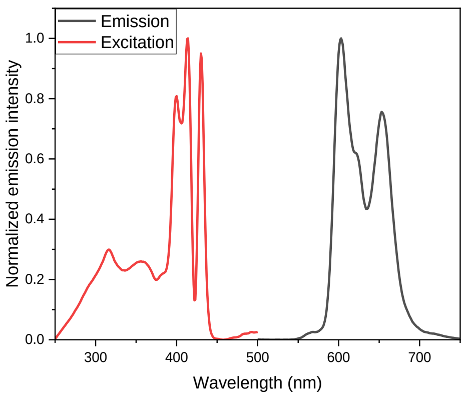

Figure S25: **ZnTPP** emission and excitation spectra (λ_{exc} = 420 nm, λ_{em} = 605 nm) in fluid THF solution ($c \approx 10^{-5}$M) at 20 °C.

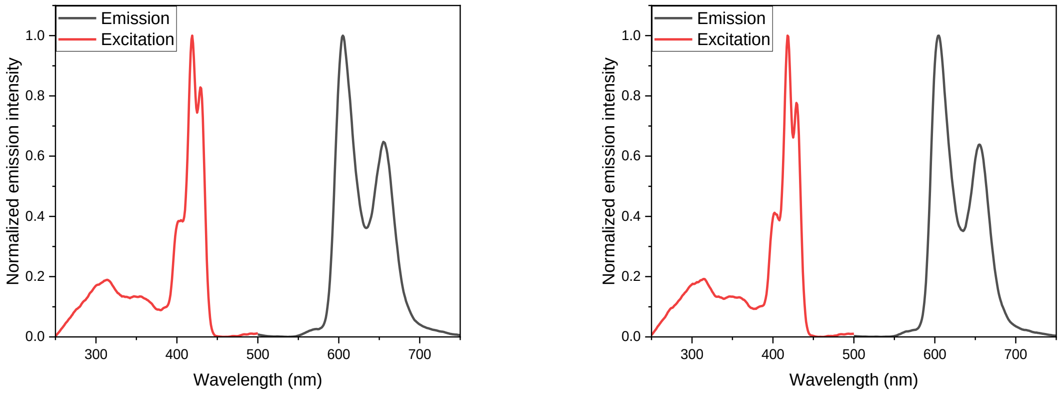

Figure S26: Emission and excitation spectra (λ_{exc} = 420 nm, λ_{em} = 605 nm) in fluid THF solution of rotaxanes **1a-H(PF$_6$)$_2$** (left) and **1a-PF$_6$** (left) ($c \approx 10^{-5}$M) at 20 °C.

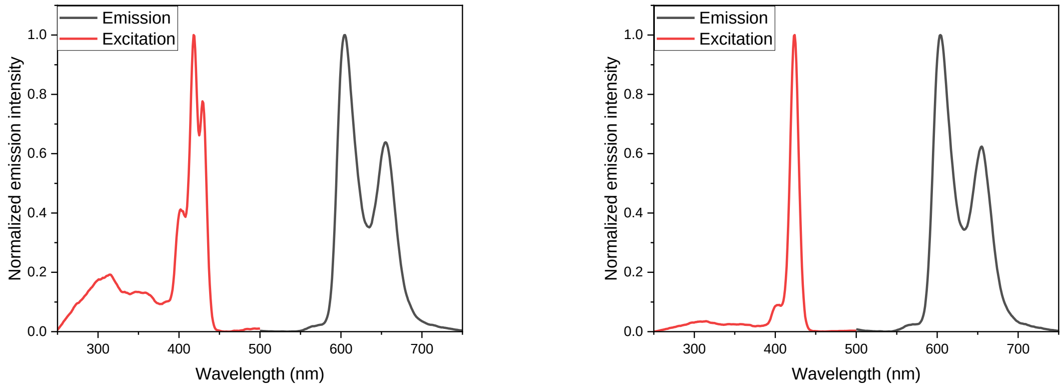

Figure S27: Emission and excitation spectra (λ_{exc} = 420 nm, λ_{em} = 605 nm) in fluid THF solution of rotaxanes **1b-H(PF$_6$)$_2$** (left) and **1b-PF$_6$** (left) ($c \approx 10^{-5}$M) at 20 °C.

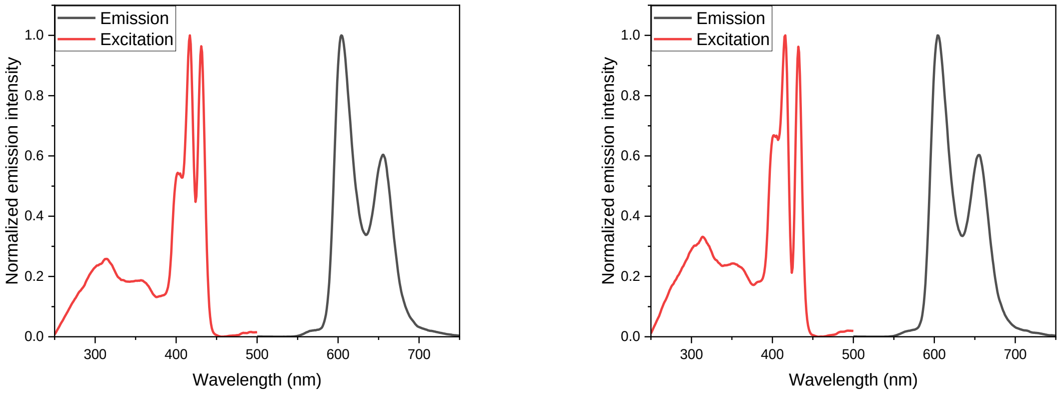

Figure S28: Emission and excitation spectra (λ_{exc} = 420 nm, λ_{em} = 605 nm) in fluid THF solution of rotaxanes **2-H(PF$_6$)$_2$** (left) and **2-PF$_6$** (left) ($c \approx 10^{-5}$M) at 20 °C.

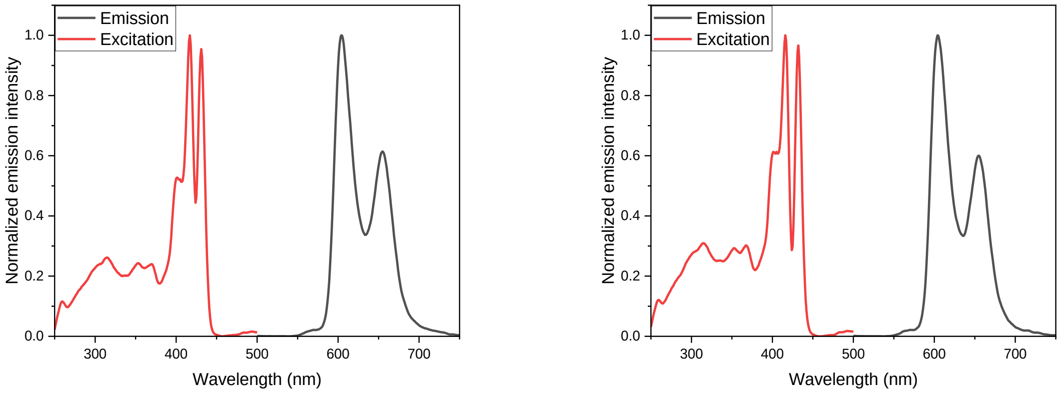

Figure S29: Emission and excitation spectra (λ_{exc} = 420 nm, λ_{em} = 605 nm) in fluid THF solution of rotaxanes **3-H(PF$_6$)$_2$** (left) and **3-PF$_6$** (left) ($c \approx 10^{-5}$M) at 20 °C.

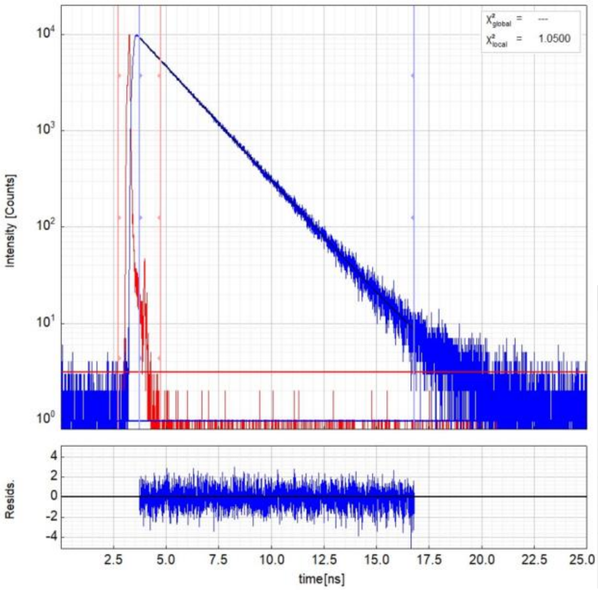

Parameter	Value	Error
A_1[kCnts/Chnl]	12.302	± 0.077
τ_1[ns]	1.8381	± 0.0038
I_1[kCnts]	5653	± 30
$A_{Rel\,1}$[%]	100.0000	± 0.0000
$I_{Rel\,1}$[%]	100.0000	± 0.0000
$Bkgr_{Dec}$[kCnts]	0.0010	± 0.0005
$Bkgr_{IRF}$[Cnts/Chnl]	3.1	± 4.5
$Shift_{IRF}$[ps]	-230.0	± 6.1
$\tau_{Av\,Int}$[ns]	1.8381	± 0.0038
$\tau_{Av\,Amp}$[ns]	1.8381	± 0.0038

Figure S30: Left: Raw time-resolved photoluminescence decay of **ZnTPP** in fluid THF solution ($c \approx 10^{-5}$ M) at 20 °C, including the residuals (λ_{exc} = 407.2 nm, λ_{em} = 605 nm). The instrument response function is shown in red. Right: Fitting parameters including pre-exponential factors and confidence limits.

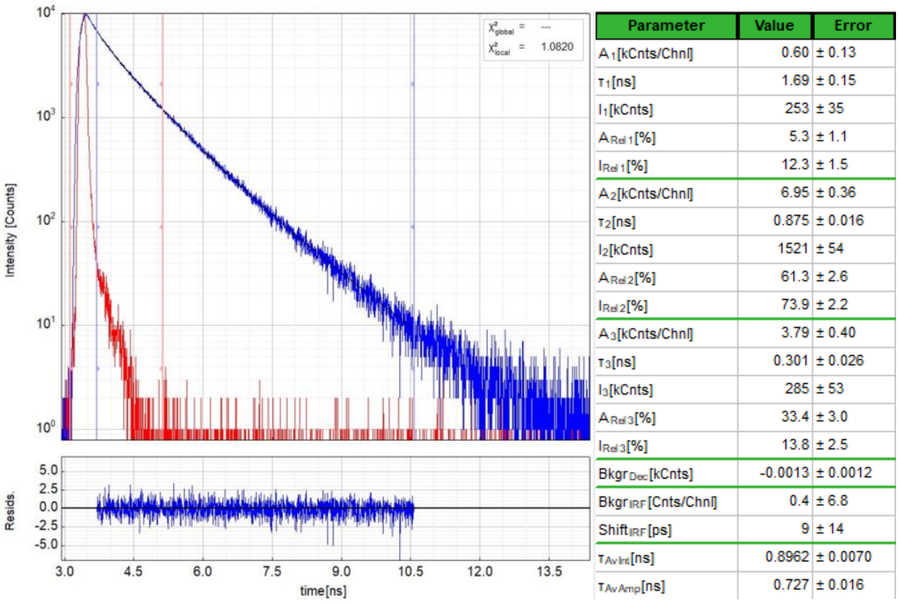

Parameter	Value	Error
A_1[kCnts/Chnl]	0.60	± 0.13
τ_1[ns]	1.69	± 0.15
I_1[kCnts]	253	± 35
$A_{Rel\,1}$[%]	5.3	± 1.1
$I_{Rel\,1}$[%]	12.3	± 1.5
A_2[kCnts/Chnl]	6.95	± 0.36
τ_2[ns]	0.875	± 0.016
I_2[kCnts]	1521	± 54
$A_{Rel\,2}$[%]	61.3	± 2.6
$I_{Rel\,2}$[%]	73.9	± 2.2
A_3[kCnts/Chnl]	3.79	± 0.40
τ_3[ns]	0.301	± 0.026
I_3[kCnts]	285	± 53
$A_{Rel\,3}$[%]	33.4	± 3.0
$I_{Rel\,3}$[%]	13.8	± 2.5
$Bkgr_{Dec}$[kCnts]	-0.0013	± 0.0012
$Bkgr_{IRF}$[Cnts/Chnl]	0.4	± 6.8
$Shift_{IRF}$[ps]	9	± 14
$\tau_{Av\,Int}$[ns]	0.8962	± 0.0070
$\tau_{Av\,Amp}$[ns]	0.727	± 0.016

Figure S31: Left: Raw time-resolved photoluminescence decay of **H-1a(PF$_6$)$_2$** in fluid THF solution ($c \approx 10^{-5}$ M) at 20 °C, including the residuals (λ_{exc} = 407.2 nm, λ_{em} = 605 nm). The instrument response function is shown in red. Right: Fitting parameters including pre-exponential factors and confidence limits.

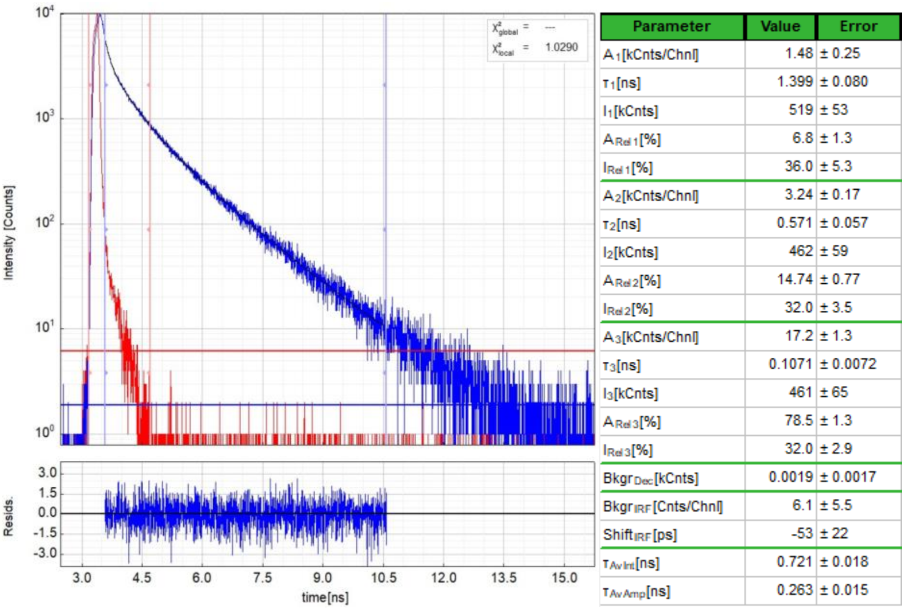

Parameter	Value	Error
A_1[kCnts/Chnl]	1.48	± 0.25
τ_1[ns]	1.399	± 0.080
I_1[kCnts]	519	± 53
$A_{Rel\,1}$[%]	6.8	± 1.3
$I_{Rel\,1}$[%]	36.0	± 5.3
A_2[kCnts/Chnl]	3.24	± 0.17
τ_2[ns]	0.571	± 0.057
I_2[kCnts]	462	± 59
$A_{Rel\,2}$[%]	14.74	± 0.77
$I_{Rel\,2}$[%]	32.0	± 3.5
A_3[kCnts/Chnl]	17.2	± 1.3
τ_3[ns]	0.1071	± 0.0072
I_3[kCnts]	461	± 65
$A_{Rel\,3}$[%]	78.5	± 1.3
$I_{Rel\,3}$[%]	32.0	± 2.9
$Bkgr_{Dec}$[kCnts]	0.0019	± 0.0017
$Bkgr_{IRF}$[Cnts/Chnl]	6.1	± 5.5
$Shift_{IRF}$[ps]	-53	± 22
$\tau_{Av\,Int}$[ns]	0.721	± 0.018
$\tau_{Av\,Amp}$[ns]	0.263	± 0.015

Figure S32: Left: Raw time-resolved photoluminescence decay of **1a-PF$_6$** in fluid THF solution ($c \approx 10^{-5}$ M) at 20 °C, including the residuals (λ_{exc} = 407.2 nm, λ_{em} = 605 nm). The instrument response function is shown in red. Right: Fitting parameters including pre-exponential factors and confidence limits.

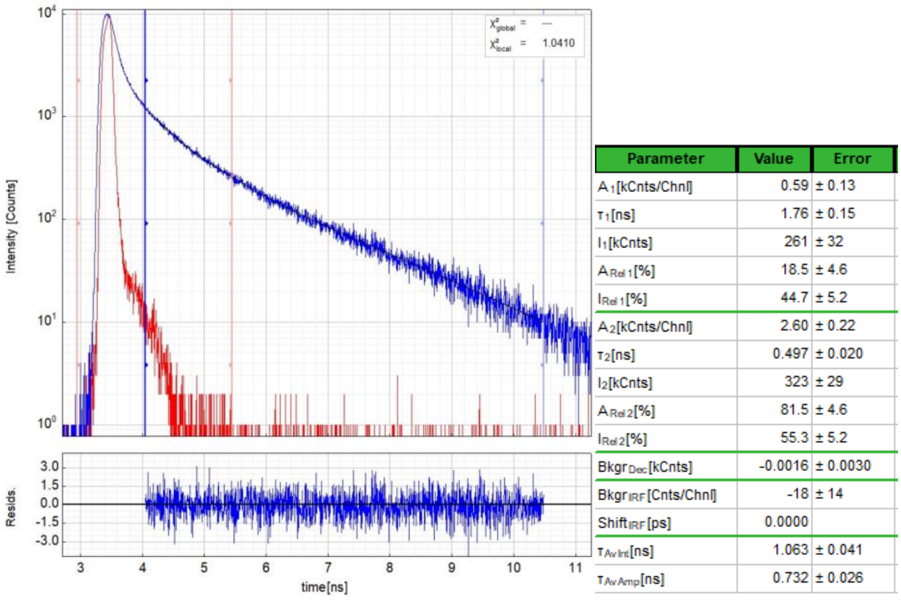

Parameter	Value	Error
A_1[kCnts/Chnl]	0.59	± 0.13
τ_1[ns]	1.76	± 0.15
I_1[kCnts]	261	± 32
$A_{Rel\,1}$[%]	18.5	± 4.6
$I_{Rel\,1}$[%]	44.7	± 5.2
A_2[kCnts/Chnl]	2.60	± 0.22
τ_2[ns]	0.497	± 0.020
I_2[kCnts]	323	± 29
$A_{Rel\,2}$[%]	81.5	± 4.6
$I_{Rel\,2}$[%]	55.3	± 5.2
$Bkgr_{Dec}$[kCnts]	-0.0016	± 0.0030
$Bkgr_{IRF}$[Cnts/Chnl]	-18	± 14
$Shift_{IRF}$[ps]	0.0000	
$\tau_{Av\,Int}$[ns]	1.063	± 0.041
$\tau_{Av\,Amp}$[ns]	0.732	± 0.026

Figure S33: Left: Raw time-resolved photoluminescence decay of **H-1b(PF₆)₂** in fluid THF solution ($c \approx 10^{-5}$ M) at 20 °C, including the residuals (λ_{exc} = 407.2 nm, λ_{em} = 605 nm). The instrument response function is shown in red. Right: Fitting parameters including pre-exponential factors and confidence limits.

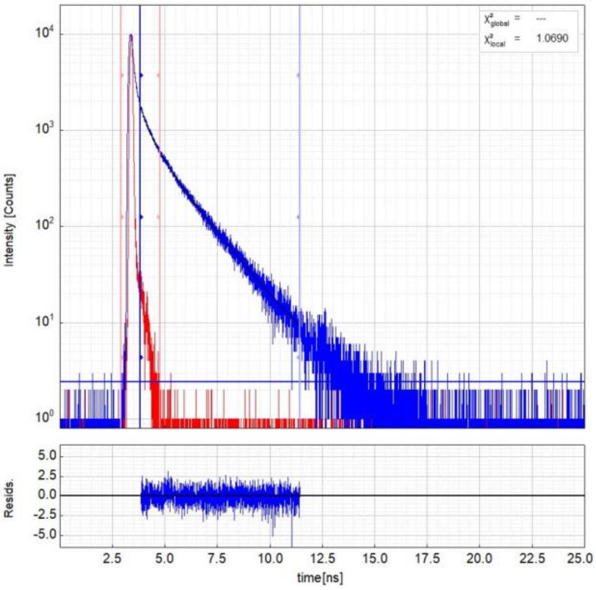

Parameter	Value	Error
A_1[kCnts/Chnl]	1.126	± 0.076
τ_1[ns]	1.567	± 0.027
I_1[kCnts]	441	± 24
$A_{Rel\,1}$[%]	29.3	± 3.0
$I_{Rel\,1}$[%]	62.3	± 1.9
A_2[kCnts/Chnl]	2.72	± 0.28
τ_2[ns]	0.393	± 0.030
I_2[kCnts]	267	± 11
$A_{Rel\,2}$[%]	70.7	± 3.0
$I_{Rel\,2}$[%]	37.7	± 1.9
$Bkgr_{Dec}$[kCnts]	0.0024	± 0.0008
$Bkgr_{IRF}$[Cnts/Chnl]	-23	± 19
$Shift_{IRF}$[ps]	0.0000	
$\tau_{Av\,Int}$[ns]	1.124	± 0.032
$\tau_{Av\,Amp}$[ns]	0.737	± 0.053

Figure S34: Left: Raw time-resolved photoluminescence decay of **1b-PF₆** in fluid THF solution ($c \approx 10^{-5}$ M) at 20 °C, including the residuals (λ_{exc} = 407.2 nm, λ_{em} = 605 nm). The instrument response function is shown in red. Right: Fitting parameters including pre-exponential factors and confidence limits.

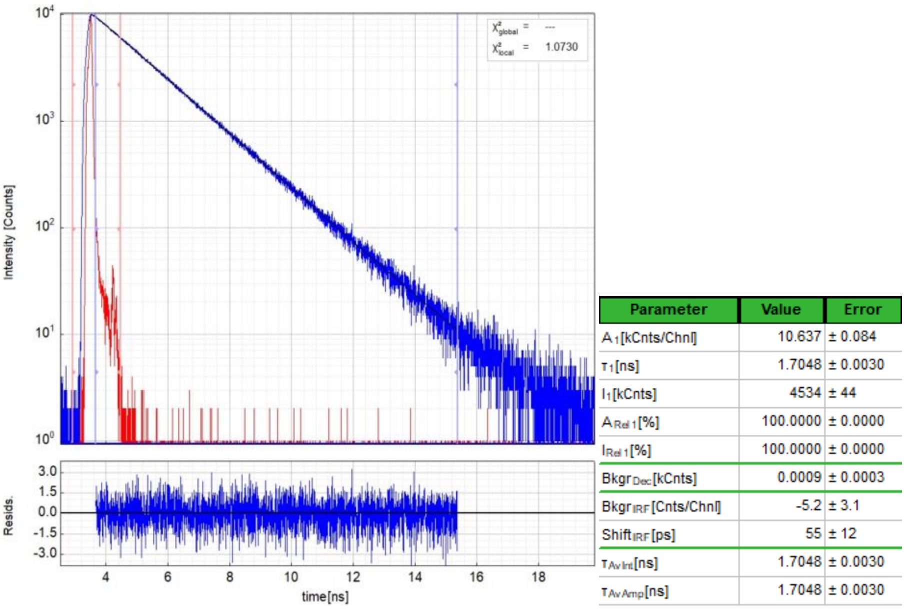

Parameter	Value	Error
A_1[kCnts/Chnl]	10.637	± 0.084
τ_1[ns]	1.7048	± 0.0030
I_1[kCnts]	4534	± 44
$A_{Rel\ 1}$[%]	100.0000	± 0.0000
$I_{Rel\ 1}$[%]	100.0000	± 0.0000
$Bkgr_{Dec}$[kCnts]	0.0009	± 0.0003
$Bkgr_{IRF}$[Cnts/Chnl]	-5.2	± 3.1
$Shift_{IRF}$[ps]	55	± 12
$\tau_{Av\ Int}$[ns]	1.7048	± 0.0030
$\tau_{Av\ Amp}$[ns]	1.7048	± 0.0030

Figure S35: Left: Raw time-resolved photoluminescence decay of **H-2(PF$_6$)$_2$** in fluid THF solution ($c \approx 10^{-5}$ M) at 20 °C, including the residuals (λ_{exc} = 407.2 nm, λ_{em} = 605 nm). The instrument response function is shown in red. Right: Fitting parameters including pre-exponential factors and confidence limits.

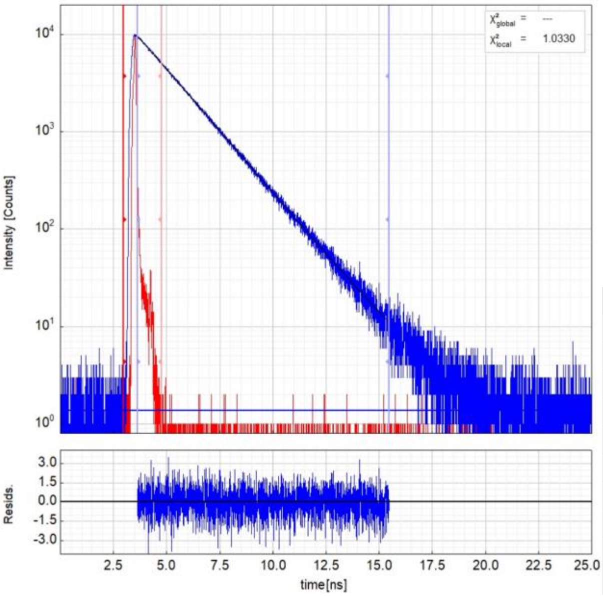

Parameter	Value	Error
A_1[kCnts/Chnl]	10.44	± 0.38
τ_1[ns]	1.7118	± 0.0027
I_1[kCnts]	4468	± 162
$A_{Rel\ 1}$[%]	100.0000	± 0.0000
$I_{Rel\ 1}$[%]	100.0000	± 0.0000
$Bkgr_{Dec}$[kCnts]	0.0014	± 0.0003
$Bkgr_{IRF}$[Cnts/Chnl]	-3.4	± 1.8
$Shift_{IRF}$[ps]	88	± 68
$\tau_{Av\ Int}$[ns]	1.7118	± 0.0027
$\tau_{Av\ Amp}$[ns]	1.7118	± 0.0027

Figure S36: Left: Raw time-resolved photoluminescence decay of **2-PF$_6$** in fluid THF solution ($c \approx 10^{-5}$ M) at 20 °C, including the residuals (λ_{exc} = 407.2 nm, λ_{em} = 605 nm). The instrument response function is shown in red. Right: Fitting parameters including pre-exponential factors and confidence limits.

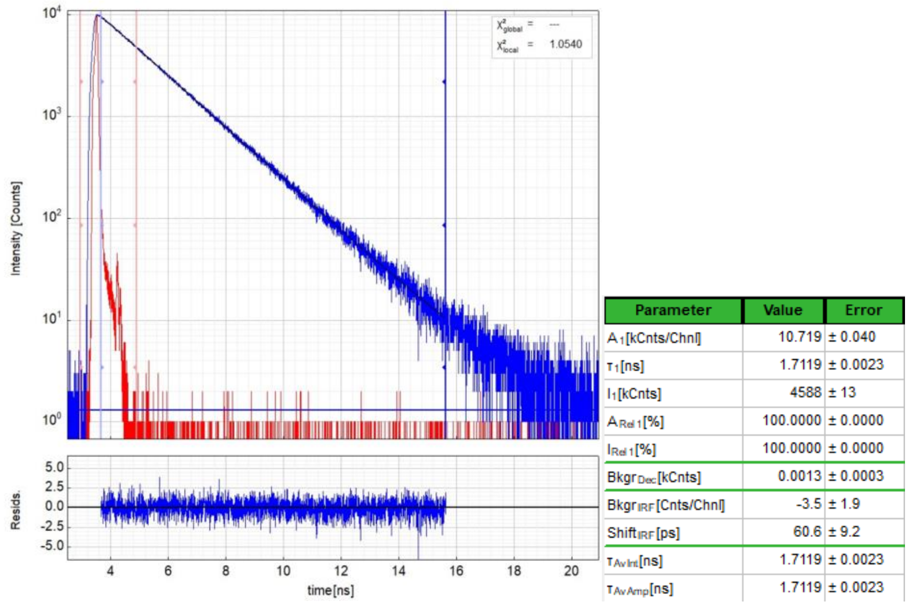

Parameter	Value	Error
A_1[kCnts/Chnl]	10.719	± 0.040
τ_1[ns]	1.7119	± 0.0023
I_1[kCnts]	4588	± 13
$A_{Rel\,1}$[%]	100.0000	± 0.0000
$I_{Rel\,1}$[%]	100.0000	± 0.0000
$Bkgr_{Dec}$[kCnts]	0.0013	± 0.0003
$Bkgr_{IRF}$[Cnts/Chnl]	-3.5	± 1.9
$Shift_{IRF}$[ps]	60.6	± 9.2
$\tau_{Av\,Int}$[ns]	1.7119	± 0.0023
$\tau_{Av\,Amp}$[ns]	1.7119	± 0.0023

Figure S37: Left: Raw time-resolved photoluminescence decay of **H-3(PF$_6$)$_2$** in fluid THF solution ($c \approx 10^{-5}$ M) at 20 °C, including the residuals (λ_{exc} = 407.2 nm, λ_{em} = 605 nm). The instrument response function is shown in red. Right: Fitting parameters including pre-exponential factors and confidence limits.

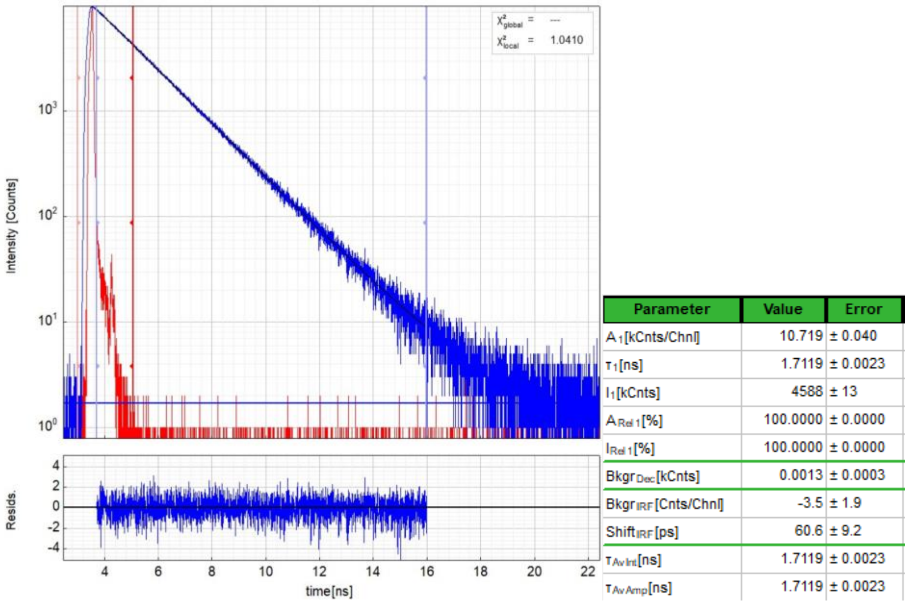

Parameter	Value	Error
A_1[kCnts/Chnl]	10.719	± 0.040
τ_1[ns]	1.7119	± 0.0023
I_1[kCnts]	4588	± 13
$A_{Rel\,1}$[%]	100.0000	± 0.0000
$I_{Rel\,1}$[%]	100.0000	± 0.0000
$Bkgr_{Dec}$[kCnts]	0.0013	± 0.0003
$Bkgr_{IRF}$[Cnts/Chnl]	-3.5	± 1.9
$Shift_{IRF}$[ps]	60.6	± 9.2
$\tau_{Av\,Int}$[ns]	1.7119	± 0.0023
$\tau_{Av\,Amp}$[ns]	1.7119	± 0.0023

Figure S38: Left: Raw time-resolved photoluminescence decay of **3-PF$_6$** in fluid THF solution ($c \approx 10^{-5}$ M) at 20 °C, including the residuals (λ_{exc} = 407.2 nm, λ_{em} = 605 nm). The instrument response function is shown in red. Right: Fitting parameters including pre-exponential factors and confidence limits.

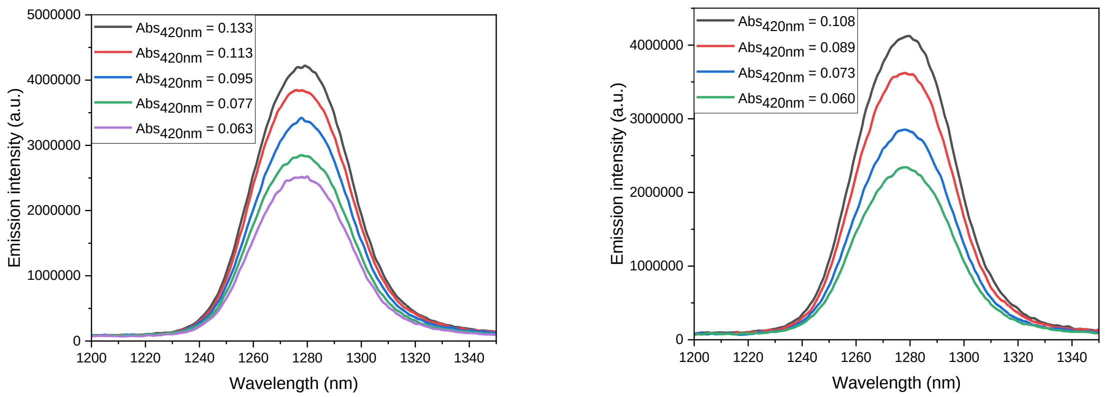

Figure S39: $^1O_2$ phosphorescence spectra (λ_{exc} = 420 nm) for **H_2TPP** (left) and **ZnTPP** (right) in THF-d_8 at 20 °C.

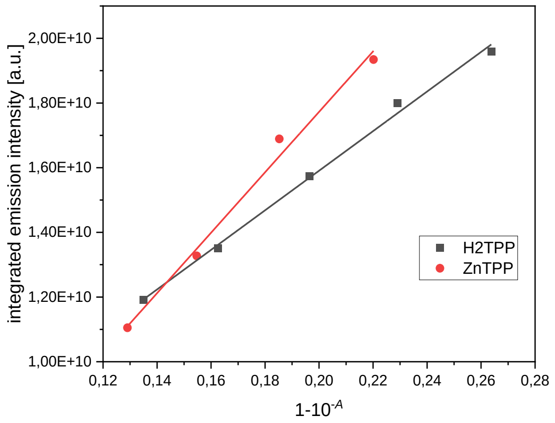

Figure S40: Integrated emission intensity of $^1O_2$ phosphorescence as function of the fraction of absorbed light in THF-d_8 at 20 °C.

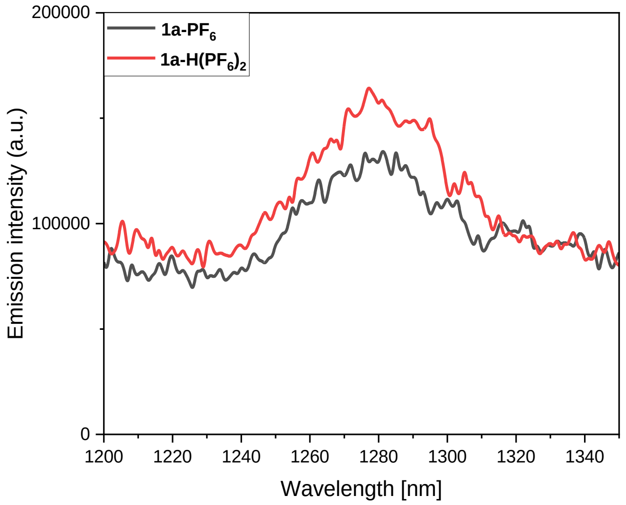

Figure S41: $^1O_2$ phosphorescence spectra for **1a-H(PF$_6$)$_2$**and **1a-PF$_6$** in THF-*d*$_8$ at 20 °C (not corrected for absorbance).

To further characterize the triplet states of the different compounds, nanosecond transient absorption spectra were measured. Figure S42 presents the different transient spectra obtained for **ZnTPP**, **2-PF$_6$**, **2-H(PF$_6$)$_2$**, **3-PF$_6$** and **3-H(PF$_6$)$_2$**. Through comparison, it is evident that the spectra obtained for the different compounds can be distinctly attributed to the influence of ZnTPP. No signals were obtained for **1a-H(PF$_6$)$_2$** and **1a-PF$_6$.**

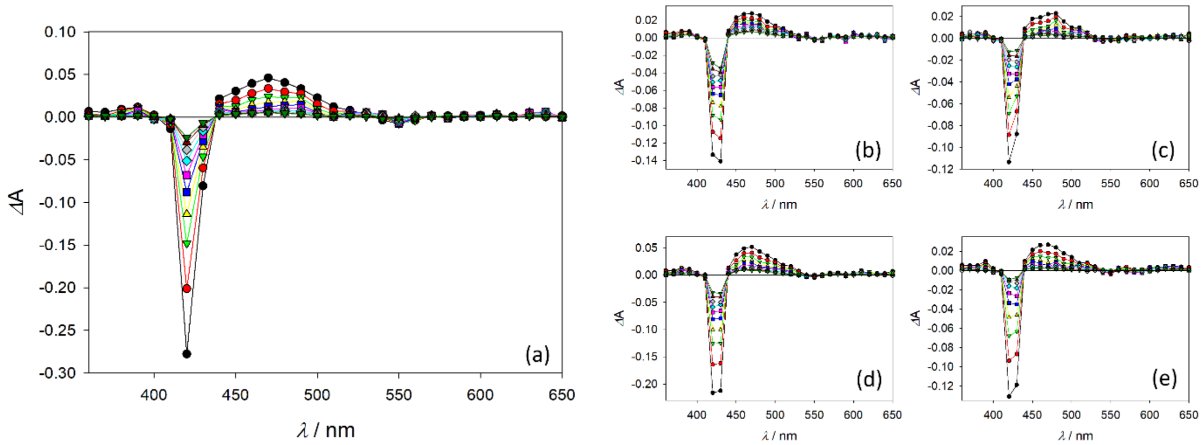

Figure S42: Nanosecond transient absorption spectra of: (a) **ZnTPP**, (b) **2-PF$_6$**, (c) **2-H(PF$_6$)$_2$**, (d) **3-PF$_6$** and (e) **3-H(PF$_6$)$_2$** obtained in toluene after excitation at 420 nm under Ar atmosphere.

In order to assess the influence of different rotaxanes on the triplet state lifetime of ZnTPP, triplet decays at 470 nm were analysed. However, achieving reproducibility in the acquired lifetimes of the compounds posed a substantial challenge, primarily because of the significant photobleaching observed in TPP upon irradiation. Different strategies (such as measurements at low temperatures or laser attenuation) were used to avoid photobleaching, but the same reproducibility problem was obtained. Three different measurements for each compound are presented as examples (see Table S1).

Table S1: Results of triplicate experiments with average triplet lifetimes and standard deviation .

	τ_1 / μs	τ_2 / μs	τ_3 / μs	$\tau_{average}$ / μs	STD
**ZnTPP**	174 ± 1	268 ± 2	192 ± 2	211	50
**2-PF_6**	378 ± 3	305 ± 3	264 ± 2	316	58
**2-$H(PF_6)_2$**	249 ± 3	262 ± 2	198 ± 1	236	34
**3-PF_6**	260 ± 1	129 ± 3	322 ± 3	237	99
**3-$H(PF_6)_2$**	78 ± 1	103 ± 1	172 ± 1	118	49

2 Supplementary methods: Theoretical calculations

2.1 Computational details

2.1.1 Generation of structures

To unveil the counterintuitive behavior of **H-1a^{2+}** and **1a^{+}**, their conformational spaces were investigated using quantum chemical calculations. To a lesser extent, we examined the truncated versions **H-1b^{2+}** and **1b^{+}** as well. Conformers were generated with the meta-dynamics (MTD)-based[24] *C*onform-*R*otamer *E*nsemble *S*ampling *T*ool *CREST*[25] (v. 2.12), employing the semi-empirical quantum mechanical (SQM) methods GFN*n*-xTB[26, 27, 28] (n = 1, 2) as well as the force field GFN-FF[29]. If not stated otherwise, the solvent THF is accounted for using the implicit solvation model ALPB[30] in all calculations. Different technical settings (see Table S2) as well as several starting points (*e.g.*, the structures as depicted in Figure S43) were used, leading to a multitude of conformer-rotamer ensembles (CREs) covering a broad range of the potential energy surface (PES). In general, *CREST* is intended for this purpose, but it reaches its limitations for the system at hand due to high system flexibility, electronic complexity, and considerable size.

Table S2: Settings for some of the conducted conformer searches for **1a^{+}**, performed with *CREST*.[23, 27, 28, 29] For better readability the "-xTB" suffix is omitted in the "level of theory" column. Similar settings were used for **H-1a^{2+}**, **1b^{+}**, and **H-1b^{2+}**, although to a lesser extent for the truncated **1b**-variants. fc = force constant.

run	level of theory	special settings	MTD simulation length [ps]
**1**	GFN2	*mrest3, ewin 50*<br>*bond constrain^{a}, fc=0.01*	20
**2**	GFN2	-	50
**3**	GFN2	-	50
**4**	GFN2	*quick*<br>*bond constrain^{a}, fc = 0.02*	250
**5**	GFN2	*bond constrain^{a}, fc = 0.02*	20
**6**	GFN2	-	10
**7**	GFN1	-	50
**8**	GFN-FF	-	500
**9**	GFN-FF	-	250
**10**	GFN-FF	*bond constrain^{a}, fc = 0.02*	250
**11**	GFN-FF	*bond constrain^{a}, fc = 0.02*	250
**12**	GFN-FF	*bond constrain^{a}, fc = 0.125*	250

a: Distances of nitrogen (of the NMe group of the triazolium-station) to the two closest oxygen atoms of the crown ether were constrained to 3.3 Å.

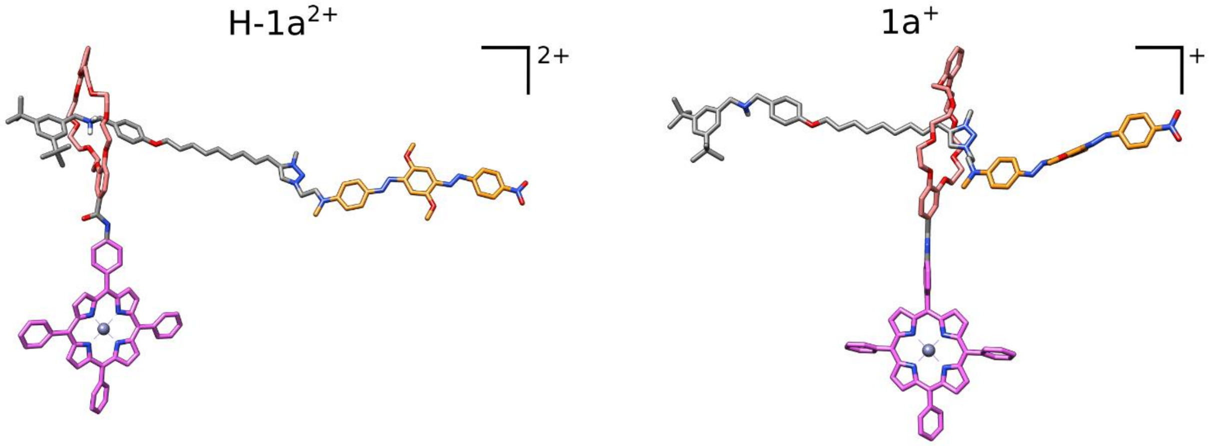

Figure S43: Start structures for many of the performed conformation searches used for **H-1a^{2+}** and **1a^{+}**. The coloring of the carbon atoms is equivalent to Fig.2 in the main article. For better clarity the triazolium-station is not colored and all H-atoms except for NH_2 are excluded. Structures were depicted with the UCSF Chimera software package.[31]

2.1.2 Computation of Gibbs free energies

To investigate potential shortcomings of the SQM methods and the low-level implicit solvation model, sets of relevant (low-lying) and representative conformers were created for both protonation states and investigated further at a higher level of theory. Low-lying conformations of the different CREST simulations were added to these sets, unless a similar structural motif was already included, or their energies were too high compared to the other conformations in the set. However, due to the large system size, these low-lying conformations in the ensembles created by CREST often included multiple different variations of the same structural motifs, therefore we also investigated molecular dynamic and meta-dynamic simulations performed during the CRE generations and manually picked out additional conformations that seemed of interest. Such conformations especially featured distinct binding motifs of the Zn atom by the quencher. Since the representative structures were evaluated on a DFT level, our goal was to prevent the neglection of a binding motif due to an underestimation of the previously used SQM methods. Additionally, conformations with almost no intramolecular folding were taken randomly out of different meta-dynamic simulation runs performed by CREST as such motifs are expected to be underestimated by the cheap solvation model used in the sampling. Gibbs free energies of the chosen structures were evaluated using a so-called multilevel ansatz. It is based on the validated assumption that the potential energy surfaces of the methods used are parallel,[32] leading to higher-level results.

The chosen conformers are divided into three subsets: *closed*, *half-open*, and *open*. The division is based on the degree of intramolecular folding exhibited by the structures. As a quantification the *S*olvent *A*ccessible *S*urface *A*rea (SASA) is used, which we computed for each conformer.[33] In the *open* conformers no or minimal folding is observed, and the Zn-atom in the ZnTPP-unit is not coordinated in its axial (octahedral) plane (*e.g.*, the conformations shown in Figure S43). The *closed* conformations exhibit a high degree of intramolecular folding, and (mostly) a coordination of the Zn-atom in the aforementioned plane. Please note that **H-1a^{2+}** shows mainly one motif, so we also evaluated *closed* conformers with no or minimal coordination of the Zn-atom but a strong folding to validate the found conformer. *Half-open* conformers show a moderate degree of folding, but in some cases still a coordinating group on the Zn-atom. Due to the varying size of the variants **1a** and **1b**, different SASA-ranges were used to identify each subset (compare Table S3).

Table S3: Ranges of the solvent accessible surface area (*SASA*) chosen for the division into the subsets *closed*, *half-open*, and *open* for **H-1a**$^{2+}$/**1a**$^{+}$ and **H-1b**$^{2+}$/**1b**$^{+}$. The *SASA* was computed using the *numsa* program package.[33]All values are given in Å^2.

subset	H-1a^{2+}/ 1a^{+}	H-1b^{2+}/ 1b^{+}
closed	*SASA* ≤ 2300	*SASA* ≤ 2100
half-open	2300 < *SASA* < 2550	2100 < *SASA* < 2300
open	2550 ≤ *SASA*	2300 ≤ *SASA*

Figure S45 and Figure S46 show some representative conformers of the **1a**-species. The **1b**-species were not of primary interest hence fewer structures were considered for this evaluation. Some representative conformers for both protonation states of these truncated variants are shown in Figure S47 and Figure S48.

The chosen structures were first optimized at the GFN2-xTB [ALPB:THF] level of theory. Subsequently, thermal corrections (G_{thermo}) are computed at the same level by employing the modified rigid-rotor-harmonic-oscillator approximation (mRRHO)[34] with the *xTB* (v. 6.5.1) program.[35] Utilizing the *TURBOMOLE* (v. 7.5.1)[36,37] program package, we then computed solvation contributions (∂G_{solv}) and electronic gas-phase energies (E_{gas}) using the PBEh-3c composite DFT method.[38] The solvation contributions are calculated with the COSMO-RS[39] implicit solvation model, a more sophisticated but considerably more costly approach than the ALPB-model.

Finally, Gibbs free energies (G_{Gibbs}) are calculated based on the following equation:

$$G_{Gibbs} = E_{gas} + G_{thermo} + \partial G_{solv} \, .$$

To facilitate comparison, only relative values ($\Delta X = X - X_{ref}$) are discussed, with the smallest value being the respective reference value X_{ref}. To put it in other words, the smaller the ΔX, the better the value.

2.2 Results and discussion

2.2.1 H-1a^{2+} and 1a^{+}: Conformer searches

Our investigations focus on the binding motifs of the ZnTPP-unit, as this is the most relevant part for the singlet oxygen production. If two conformers differ only in the arrangement in other parts of the rotaxane, *e.g.*, a rotation of a methyl-group, these conformers are considered the same structural motif. In order to compare as many different motifs as possible, we only investigated the most stable structure per motif further. A numbering scheme for the discussion of distances and coordination sites found for the different conformers is shown in Figure S44.

Conformer searches for **H-1a**$^{2+}$ passed without any difficulties. In almost all performed conformer searches, the same *closed* conformational motif was identified (see Figure S45). It shows a high degree of intramolecular folding together with a coordination of the Zn-atom in one of its axial positions by the first benzene of the quencher (Q6). These findings suggest a deep minimum for **H-1a**$^{2+}$ *i.e.* a limited conformational flexibility. This possibly results from the high charge of $+2$ of the system, leading to strong (intramolecular) electrostatic interactions, which stabilize the *closed* conformations.

In contrast, finding the most relevant low-lying conformer(s) of **1a**$^{+}$ proved to be a challenging task. A variety of distinct conformations were observed, with many of them featuring a different segment of the rotaxane coordinating to the Zn-atom. Moreover, several less folded (*half-open*) conformers with comparable energies to *closed* conformers were found, a phenomenon not observed for **H-1a**$^{2+}$. These results imply a greater degree of flexibility for **1a**$^{+}$, indicating a flatter Potential Energy Surface (PES). Some of the relevant conformations found are depicted in Figure S46.

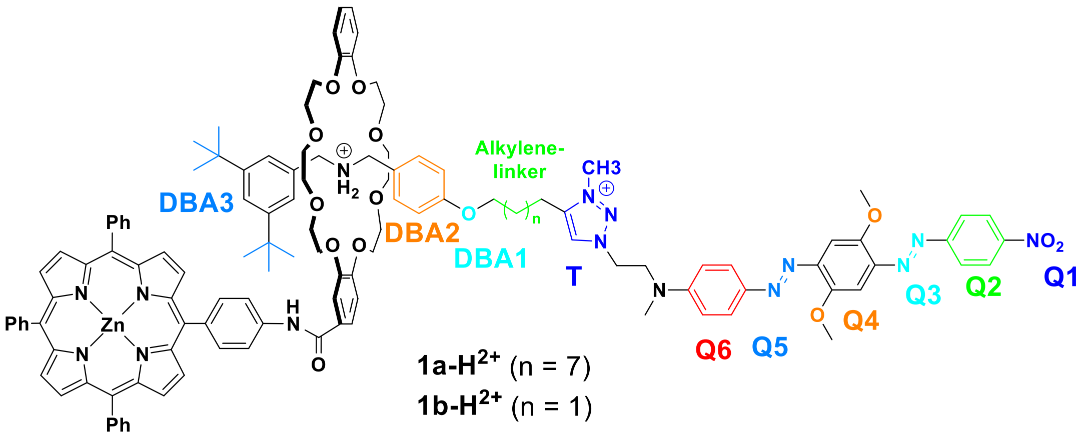

Figure S44: Structure of rotaxanes **H-1a/b**$^{2+}$ with numbering scheme for discussion of relative distances of subunits found during conformer searches.

2.2.2 H-1a^{2+} and 1a^{+}: Gibbs free energies

Inclusion of explicit solvent molecules in the MTD simulations is computationally too demanding, however, interactions between THF, Zn and other groups can be expected. These interactions should decrease with increasing intramolecular folding. The ALPB solvation model used so far was chosen due to its low computational cost but may underestimate the approximated solvent-solute effects. An indication for this is that most low-lying conformations found were in a *closed* form with strong folding. Therefore, we computed Gibbs energies at a higher level of theory, ruling out possible deficiencies of the SQM-based solvation model. Consequently, we created three different subsets: *open*, *half-open*, and *closed* (see computational details for further explanations). Table S4 and Table S5 show the computed energy contributions and resulting relative Gibbs free energies for all investigated structures of **H-1a**$^{2+}$ and **1a**$^{+}$, respectively.

We begin our evaluation with the protonated form: **H-1a**$^{2+}$ (see Table S4). Comparing the ΔE_{gas} contributions for the different subsets shows that a maximization of intramolecular interactions in the structure is more desirable, hence closed structures are generally more favored in the gas phase. However, the solvation contributions ($\Delta\partial G_{solv}$) show an inverse trend since the solvent accessible surface area (SASA) is maximized in open structures. Consequently, the interaction between the system and the implicitly modeled solvent is increased. A similar trend can be observed for the thermal contributions ΔG_{thermo}. The conformational searches performed with *CREST* do not include thermal contributions and crudely approximate $\Delta\partial G_{solv}$. Thereafter, it became evident why this approach shows a bias towards strongly folded conformations. Nevertheless, the calculated higher-level Gibbs energies suggest that *closed* **H-1a**$^{2+}$ conformations are thermodynamically preferred, despite unfavorable solvation and thermal contributions.

Table S4: Chosen structures of **H-1a**$^{2+}$ divided in the three categories *closed*, *half-open*, and *open*, based on the respective solvent accessible surface area (SASA [Å²]). For each conformer, relative gas phase (ΔE_{gas}), solvation ($\Delta\partial G_{solv}$), and thermal (ΔG_{thermo}) energy contributions together with relative Gibbs energies (ΔG_{Gibbs}) are given. Energies are given relative to the respective lowest value found [kcal/mol]. For a better overview the values in each respective column are highlighted in color from green (low) to red (high).

subset	conformer	SASA [Å²]	Zn coord. group[d]	ΔE_{gas}	$\Delta\partial G_{solv}$	ΔG_{thermo}	ΔG_{Gibbs}
closed	1[a]	1916	Q6	1.32	48.06	9.33	2.63
	2[b]	1931	Q6	0.00	48.25	7.82	0.00
	3	1951	Q6	13.98	49.17	9.58	16.64
	4	1982	Alkylene linker	3.35	52.93	8.79	8.99
	5	2061	-	14.50	42.21	8.31	8.95
	6	2163	T	27.34	45.53	7.43	24.23
	7	2195	-	45.48	33.69	7.98	31.08
	8	2225	Q1	68.14	27.92	6.54	46.53
	9	2240	T	28.35	37.87	6.03	16.17
	10	2293	Q6	37.91	27.69	3.97	13.50
half-open	11	2392	-	68.14	15.12	3.42	30.61
	12	2444	-	72.64	13.34	6.45	36.35
	13	2449	-	56.75	19.81	6.85	27.34
	14	2451	-	55.10	19.63	5.19	23.84
	15	2456	-	73.20	7.99	4.11	29.22
	16	2479	-	85.38	6.80	3.54	39.64
	17	2532	-	79.66	10.77	3.12	37.47
	18	2544	-	63.00	13.50	0.71	21.12
open	19	2555	-	70.51	7.44	4.02	25.89
	20	2569	-	59.16	10.64	3.06	16.79
	21	2574	-	67.54	11.15	3.93	26.53
	22	2582	-	73.06	5.42	4.84	27.25
	23	2592	-	60.98	16.22	2.86	23.98
	24	2612	-	61.89	9.83	2.28	17.91
	25	2638	-	63.27	9.53	2.80	19.53
	26	2648	-	71.69	5.42	4.35	25.38
	27	2653	-	67.09	7.47	0.05	18.53
	28	2662	-	71.01	9.27	2.13	26.33
	29	2668	-	73.85	9.47	2.27	29.51
	30	2679	-	87.51	0.00	2.96	34.39
	31[c]	2733	-	51.87	9.83	0.00	5.62

a: most stable conformer according to CREST(GFN2-xTB); b: most stable conformer according to PBEh-3c c: conformation shown in Figure S43; d: groups as depicted in Figure S44.

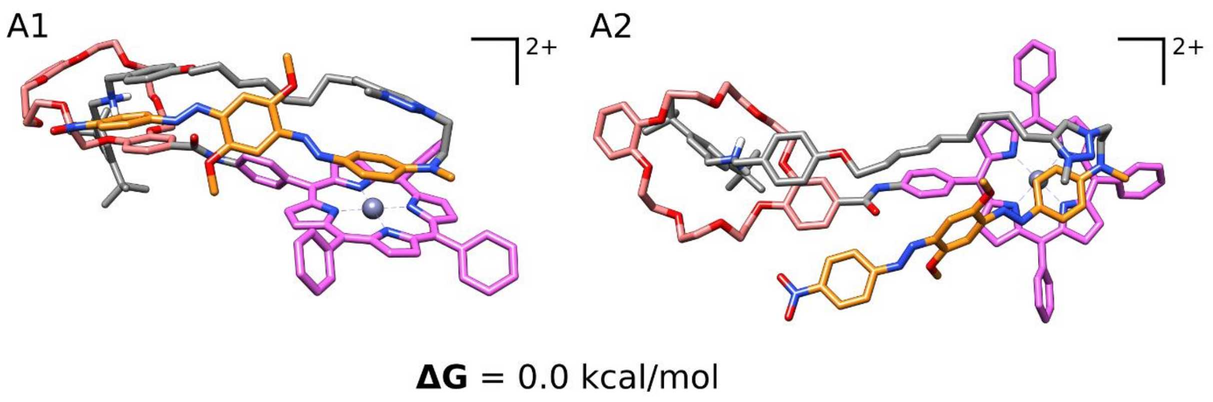

Figure S45: Most stable **H-1a**$^{2+}$ conformation found. Coloring of the carbon atoms is equivalent to Fig.2 in the main article. For better clarity the triazolium-station is not colored and all H-atoms except for NH_2 are excluded. Referring to Table S4, A1/A2: side/top view of conformer 2 (*closed*). Structures were depicted with the *UCSF Chimera* software package.[31]

Next, let us investigate the deprotonated **1a**$^{+}$ state (see Table S5). The previously described trends remain, with $\boldsymbol{\Delta\partial G_{solv}}$ and $\boldsymbol{\Delta G_{thermo}}$ generally favoring *open* structures. Similarly, *closed* conformations are beneficial in terms of the $\boldsymbol{\Delta E_{gas}}$ contribution. However, *half-open* and *open* conformers tend to be more stable than *closed* ones for **1a**$^{+}$, which is clearly different from the previously discussed case of the protonated state **H-1a**$^{2+}$. This confirms a significantly higher flexibility for **1a**$^{+}$, with many different binding motifs for the Zn moiety.

Table S5: Chosen structures of **1a**$^+$ divided in the three categories *closed*, *half-open*, and *open*, based on the respective solvent accessible surface area (SASA [Å^2]). For each conformer, relative gas phase (ΔE_{gas}), solvation ($\Delta\partial G_{solv}$), and thermal (ΔG_{thermo}) energy contributions together with relative Gibbs energies (ΔG_{Gibbs}) are given. Energies are given relative to the respective lowest value found [kcal/mol]. For a better overview the values in each respective column are highlighted in color from green (low) to red (high).

subset	conformer	SASA [Å^2]	Zn coord. group[e]	ΔE_{gas}	$\Delta\partial G_{solv}$	ΔG_{thermo}	ΔG_{Gibbs}
closed	1	1738	Q1, DBA3	1.72	39.29	9.24	18.22
	2[a]	1878	Q1	4.38	31.52	9.33	13.21
	3	1906	DBA3	9.29	33.55	9.10	19.92
	4	1965	Q2, DBA3	26.39	26.95	8.33	29.64
	5	2097	Q1	20.91	27.61	6.66	23.15
	6	2115	Q4	8.65	25.76	7.11	9.50
	7	2127	Q1	27.07	27.61	6.10	28.76
	8	2138	Q1, DBA3	23.96	19.72	6.17	17.82
	9[b,c]	2168	Q2	0.00	24.69	7.34	0.00
	10	2174	DBA1	26.62	22.85	7.20	24.65
	11	2195	-	24.63	19.36	7.02	18.99
	12	2253	-	20.25	21.04	6.52	15.79
half-open	13	2316	Q2	10.12	17.66	5.42	1.17
	14	2358	Q2	14.81	16.47	3.66	2.91
	15	2391	DBA2	37.33	10.15	4.57	20.02
	16[c]	2449	-	21.88	14.21	4.66	8.72
	17	2507	-	32.58	12.17	0.56	13.29
	18	2515	-	61.13	10.01	2.05	41.17
	19	2519	-	32.32	9.38	3.94	13.62
	20	2526	-	46.57	11.70	3.62	29.87
open	21	2591	-	54.87	9.54	3.18	35.56
	22	2639	-	34.34	4.14	2.18	8.63
	23	2685	-	57.07	3.32	0.03	28.40
	24	2686	-	41.58	5.18	1.09	15.83
	25	2695	-	39.83	2.71	0.00	10.51
	26[d]	2764	-	39.88	0.00	0.25	8.11

a: most stable conformer according to CREST(GFN2-xTB); b: most stable conformer according to PBEh-3c c: conformations shown in Figure 6 in the main article; d: conformation shown in Figure S43; e: groups as depicted in Figure S44.

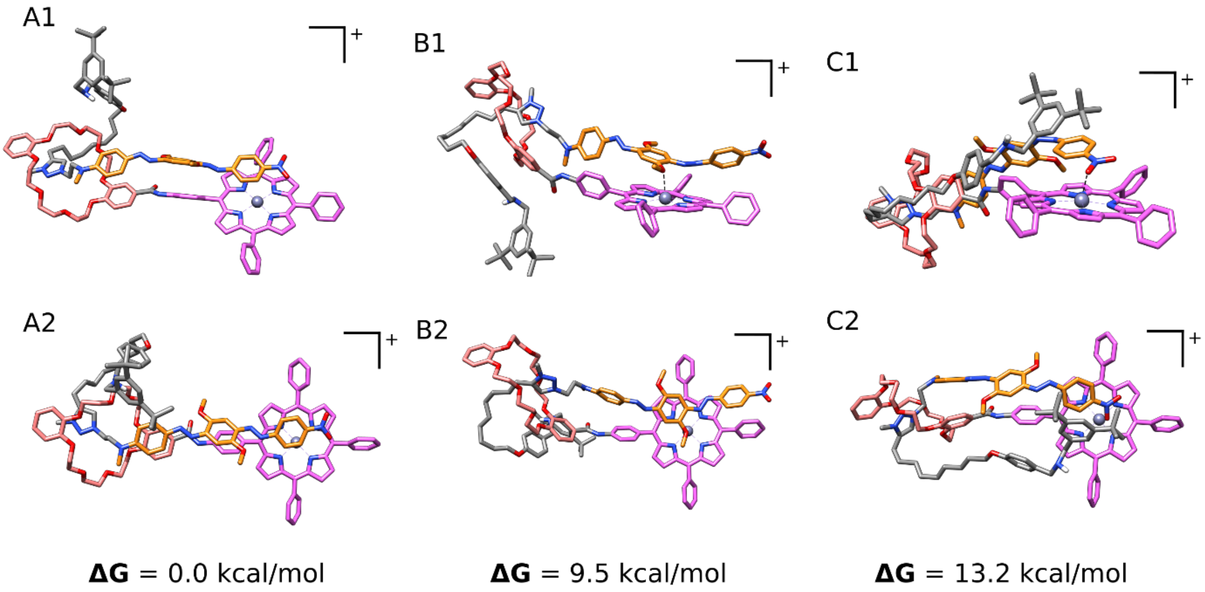

Figure S46: Three relevant **1a**$^{+}$ conformations found with their respective relative Gibbs energies. Coloring of the carbon atoms is equivalent to Fig. 2 in the main article. For better clarity the triazolium-station is not colored and all H-atoms except for NH_2 are excluded. Referring to Table S5, A1/A2: side/top view of conformer 9 (*half-open*); B1/B2: side/top view of conformer 6 (*closed*); C1/C2: side/top view of conformer 2 (*closed*). Structures were depicted with the *UCSF Chimera* software package.[31]

To conclude, **H-1a**$^{2+}$ is mostly found in one dominant strongly folded (*closed*) conformation, with the Zn-atom itself being almost always coordinated by the first Benzene (Q6) in the BHQ unit. In contrast, **1a**$^{+}$ has many comparably stable binding motifs, and proved to be much more flexible. Consequently, the ZnTPP-unit and the quencher are less frequently in a close proximity, rendering the Zn-atom more freely accessible. As the $^{1}O_2$ production is directly correlated to the diffusion of O_2 to the Zn-atom, the low singlet oxygen quantum yield observed in the experiment for **H-1a**$^{2+}$ is likely to be an effect of the shielded Zn-atom. Furthermore, the energy transfer from ZnTPP to the BHQ moiety is facilitated, when they are in a close spatial position as found for **H-1a**$^{2+}$. *Vice versa*, the Zn-atom in the deprotonated **1a**$^{+}$ species is much more likely to be accessible for O_2, in addition, the energy transfer from ZnTPP to BHQ is comparatively hindered due to larger distances, resulting in the significantly higher singlet oxygen production observed in the experiment.

The theoretical data is therefore consistent with the experimental findings where the *on*- and *off*-states are reversed compared to what was initially envisaged in the design of **1a**$^{+}$/ **H-1a**$^{2+}$. Note that the coordination of explicit THF molecules to the Zn-atom is in competition to the O_2 molecules in solution and the intramolecular coordinating groups. The influence on the singlet oxygen production, however, is likely similar for both protonation states, so that this effect cancels out in the discussed comparison. Explicit THF molecules are likely to outcompete weakly coordinating groups (*e.g.*, $^{t}B_u$) in the coordination of the zinc atom. Finding these coordinations in our calculations therefore merely indicates steric hindrances posed by those groups.

2.2.3 **H-1b^{2+}** and **1b^{+}**: Conformer searches

Since no significant singlet oxygen production was measured for **H-1b^{2+}** as well as for **1b^{+}**, they were investigated to a lesser extent. The conducted simulations show a decreased conformational space and a reduced flexibility for both variants, which is likely due to the shorter alkylene spacer. Consequently, conformations with a coordinated Zn-atom have been predominantly found for both **1b**-variants. Contrary to the longer **1a**-variants, the obtained low-lying conformers tend to be structurally similar for both truncated protonation states. As a result, several conformers of **H-1b^{2+}** and **1b^{+}** exhibit the same coordinating groups at the ZnTPP-unit (see Table S6 and Table S7). Two low-lying conformations of **H-1b^{2+}** and **1b^{+}** are shown in Figure S47 and Figure S48 respectively.

2.2.4 **H-1b^{2+}** and **1b^{+}**: Gibbs free energies

Due to the reduced flexibility discussed, fewer conformations per subsets (4 - 8) were chosen for the subsequent Gibbs free energy calculations. The obtained values for **H-1b^{2+}** are shown in Table S6, while those of **1b^{+}** are shown in Table S7.

In general, E_{gas}, G_{thermo}, and ∂G_{solv} follow the same trends as described for their longer variants. In contrast to **H-1a^{2+}/1a^{+}**, the difference in the ΔG_{Gibbs} distributions for **H-1b^{2+}/1b^{+}** is less pronounced, as both **1b**-variants have favorable low-lying *closed* conformers. In the case of **1b^{+}**, the energies of *closed* and *half-open* conformers are generally comparable. However, *half-open* conformers are highly likely to exhibit a coordinating group at the ZnTPP-unit (compare Table S7). Therefore, the Zn-atoms in both protonation states of **1b** are preferentially found in coordination and consequently exhibit closer contact with the BHQ-unit. This result is consistent with experimental findings, where **1b^{+}** and **H-1b^{2+}** showed almost the same vanishing singlet oxygen production, similar to that of **H-1a^{2+}**.

Table S6: Chosen structures of **H-1b^{2+}** divided in the three categories *closed*, *half-open*, and *open*, based on the respective solvent accessible surface area (SASA [Å^2]). For each conformer, relative gas phase (ΔE_{gas}), solvation ($\Delta\partial G_{solv}$), and thermal (ΔG_{thermo}) energy contributions together with relative Gibbs energies (ΔG_{Gibbs}) are given. Energies are given relative to the respective lowest value found [kcal/mol]. For a better overview the values in each respective column are highlighted in color from green (low) to red (high).

subset	conformer	SASA [Å^2]	Zn coord. group[c]	ΔE_{gas}	$\Delta\partial G_{solv}$	ΔG_{thermo}	ΔG_{Gibbs}
closed	1[b]	1903	Q6	0.00	45.99	7.86	13.71
	2[a]	2028	Q4	7.33	26.27	6.54	0.00
	3	2035	Q4	16.57	25.76	5.91	8.10
	4	2044	Q5	12.30	28.01	6.33	6.50
	5	2054	Q5	28.04	24.40	6.72	19.03
	6	2100	Q3	19.09	21.10	4.63	4.69
half-open	7	2105	-	36.55	24.22	4.19	24.81
	8	2192	-	44.63	10.56	3.89	18.95
	9	2297	Q1	46.82	15.20	4.30	26.18
	10	2374	-	35.09	21.09	1.40	17.44
open	11	2435	-	35.04	13.86	3.15	11.91
	12	2441	-	48.01	3.41	1.16	12.43
	13	2452	-	63.92	0.00	1.14	24.91
	14	2462	-	47.99	1.72	1.37	10.95
	15	2466	-	62.91	4.44	0.37	27.58
	16	2553	-	54.16	2.21	0.00	16.24

a: most stable conformer according to CREST(GFN2-xTB); b: most stable conformer according to PBEh-3c
c: groups as depicted in Figure S44.

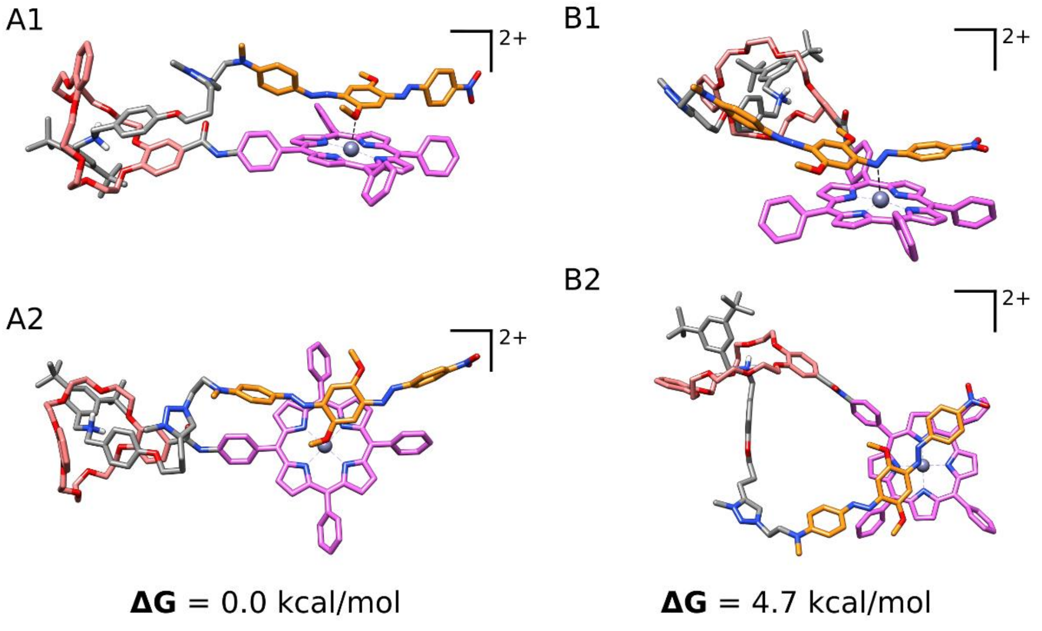

Figure S47: Two relevant **H-1b^{2+}** conformations with their respective relative Gibbs energies. Coloring of the carbon atoms is equivalent to Fig.2 in the main article. For better clarity the triazolium-station is not colored and all H-atoms except for NH_2 are not shown. Referring to Table S6: A1/A2, side/top view of conformer 2 (*closed*); B1/B2: side/top view of conformer 6 (*closed*). Structures were depicted with the *UCSF Chimera* software package.[31]

Table S7: Chosen structures of **1b**$^{+}$ divided in the three categories *closed*, *half-open*, and *open*, based on the respective solvent accessible surface area (SASA [Å^2]). For each conformer, relative gas phase (ΔE_{gas}), solvation ($\Delta\partial G_{solv}$), and thermal (ΔG_{thermo}) energy contributions together with relative Gibbs energies (ΔG_{Gibbs}) are given. Energies are given relative to the respective lowest value found [kcal/mol]. For a better overview the values in each respective column are highlighted in color from green (low) to red (high).

subset	conformer	SASA [Å^2]	Zn coord. group[c]	ΔE_{gas}	$\Delta\partial G_{solv}$	ΔG_{thermo}	ΔG_{Gibbs}
closed	1[a]	1879	Q1	3.89	15.10	6.94	1.20
	2	1976	Q1	20.94	17.31	4.17	17.68
	3	2032	Q1	22.40	17.84	6.21	21.71
	4[b]	2077	Q4	0.00	20.43	5.30	0.99
	5	2082	Q4	0.40	20.20	5.07	0.93
	6	2090	-	8.50	20.27	4.13	8.17
half-open	7	2138	Q1	25.31	4.94	4.02	9.54
	8	2145	Q4	7.77	15.29	3.13	1.44
	9	2208	Q1	34.34	7.53	2.41	19.54
	10	2228	Q1	29.15	8.17	3.37	15.95
	11	2248	Q3	24.33	5.91	1.25	6.74
	12	2256	Q1	47.19	6.08	2.09	30.62
	13	2266	-	14.90	8.49	1.35	0.00
open	14	2424	-	33.71	2.65	1.18	12.80
	15	2444	-	31.74	2.83	0.22	10.05
	16	2451	-	39.15	0.00	1.35	15.76
	17	2495	-	38.57	0.31	0.00	14.14

a: most stable conformer according to CREST(GFN2-xTB); b: most stable conformer according to PBEh-3c
c: groups as depicted in Figure S44.

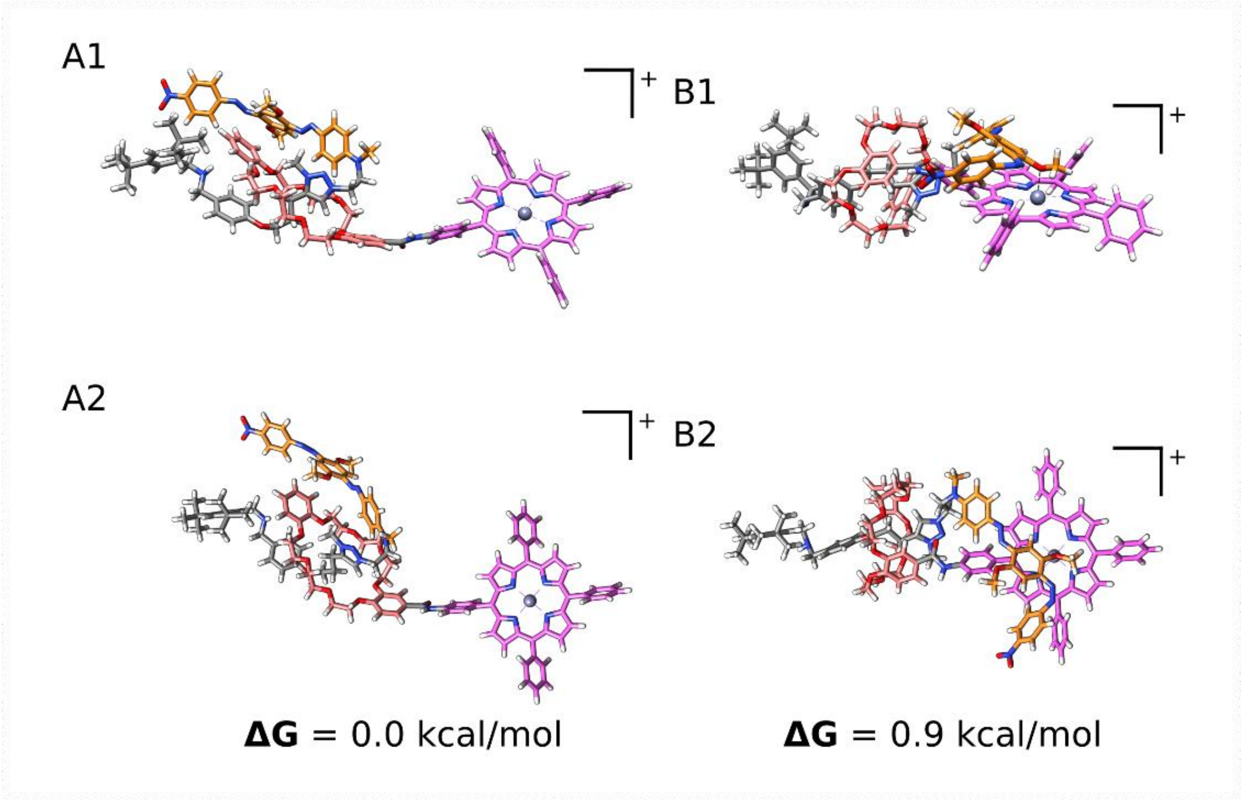

Figure S48: Two relevant **1b**$^+$ conformations with their respective relative Gibbs energies. Coloring of the carbon atoms is equivalent to Fig.2 in the main article. For better clarity the triazolium-station is not colored and all H-atoms except for NH_2 are not shown. Referring to Table S7, A1/A2: side/top view of conformer 13 (*half-open*); B1/B2: side/top view of conformer 5 (*closed*). Structures were depicted with the *UCSF Chimera* software package.[31]

2.3 Förster radius

We briefly investigated whether the theoretical results can be used to explain the measured singlet oxygen productions on a quantitative level. For this purpose, we tried to obtain Förster radii (distance between the BHQ quencher and the ZnTPP-unit) for the different conformers of **H-1a**$^{2+}$ and **1a**$^+$, to compare to the calculated value of 2.8 nm. During evaluation, multiple questions arise.

Due to the large spatial extent of the BHQ quencher, it is difficult to determine a specific point for the distance measurements. Simply taking the middle of the BHQ unit seems unreasonable and does not do justice to the complex problem of energy transfer.

We therefore tried to specify a starting *start*, middle *mid*, and ending *end* point of the unit for which we measured distances to the Zn-atom of the ZnTPP-unit. The *start* position was defined as the nitrogen atom of the NMe group (next to Q6). The *mid* of the quencher was defined as the center of the benzene ring (center of Q4). Lastly, the nitrogen atom of the NO_2 group (Q1) was defined as the *end* position. The obtained data is shown in Table S8 and Table S9.

The distances increase on average going from *closed* to *half-open*, and finally *open* conformations. However, the obtained distances are very dependent on the structural motifs of the conformations, making a direct comparison between distinct motifs difficult. An example would be the comparison of conformers 6 and 9 of **1a**$^+$ which are both

shown in Figure S46. In the first case (conformer 6), the shortest distance is observed between Zn and the center of the BHQ unit (*mid*). In the second case (conformer 9), the Zn atom is closest to the *end* of the quencher. Since many different structural motifs are being compared, no clear trends can be observed for the measured Zn-*start*, Zn-*mid*, and Zn-*end* distances. Averaging over the measured distances (see last rows of Table S8 and Table S9) did not seem to be a viable solution either.

Furthermore, missing explicit solvent molecules may have a significant influence on the measured distances, resulting in a considerable inaccuracy of the data. For these reasons, we have not considered this approach further.

Table S8: Relative Gibbs energies (ΔG_{Gibbs}) and measured distances for the **H-1a**$^{2+}$ conformations discussed in Table S4. The distances were measured between the Zn-atom of the ZnTPP-unit and the *start*, *mid*, and *end* of the BHQ quencher unit. All distances are given in Å. *start*: distance to nitrogen of NMe group (next to Q6); *mid*: distance to center of benzene (Q4); *end*: distance to nitrogen of NO_2 (Q1). Mean distances for each subset are given at the bottom (mean values of given numbers, not Boltzmann averaged). For a better overview the Gibbs energies are highlighted in color from green (low) to red (high).

subset	conformer	ΔG_{Gibbs}	distance Zn-atom to quencher / Å		
			start	mid	end
closed	1	2.63	3.99	7.69	15.44
	2	0.00	3.97	7.74	15.68
	3	16.64	4.29	7.87	15.51
	4	8.99	7.32	8.68	14.78
	5	8.95	9.91	10.45	15.53
	6	24.23	5.61	10.88	15.31
	7	31.08	12.98	14.69	14.98
	8	46.53	8.95	10.16	2.96
	9	16.17	4.83	11.09	19.90
	10	13.50	4.21	8.10	16.47
half-open	11	30.61	24.16	30.11	30.15
	12	36.35	13.85	19.69	24.00
	13	27.34	15.68	22.35	30.98
	14	23.84	16.01	22.24	30.74
	15	29.22	24.49	29.79	28.50
	16	39.64	17.94	18.38	14.18
	17	37.47	21.71	14.27	6.98
	18	21.12	16.49	18.55	25.54
open	19	25.89	24.85	28.36	33.51
	20	16.79	30.28	27.09	25.95
	21	26.53	13.40	19.10	26.64
	22	27.25	21.20	26.33	32.79
	23	23.98	30.11	35.41	42.08
	24	17.91	34.81	43.45	52.21
	25	19.53	11.19	16.72	25.26
	26	25.38	32.76	35.51	39.26
	27	18.53	29.21	28.65	26.65
	28	26.33	15.24	17.81	24.09
	29	29.51	11.77	8.97	11.87
	30	34.39	29.42	37.03	45.71
	31	5.62	23.60	32.11	40.26
closed mean	1 - 10		6.61	9.74	14.66
half-open mean	11 - 18		18.79	21.92	23.88
open mean	19 - 31		23.68	27.43	32.79

Table S9: Relative Gibbs energies (ΔG_{Gibbs}) and measured distances for the **1a**$^{+}$ conformations discussed in Table S5. The distances were measured between the Zn-atom of the ZnTPP-unit and the *start*, *mid*, and *end* of the BHQ quencher unit. All distances are given in Å. *start*: distance to nitrogen of NMe group (next to Q6); *mid*: distance to center of benzene (Q4); *end*: distance to nitrogen of NO_2 (Q1). Mean distances for each subset are given at the bottom (mean values of given numbers, not Boltzmann averaged). For a better overview the Gibbs energies are highlighted in color from green (low) to red (high).

subset	conformer	ΔG_{Gibbs}	distance Zn-atom to quencher / Å		
			start	mid	end
closed	1	18.22	13.88	6.65	4.46
	2	13.21	15.83	8.93	2.97
	3	19.92	15.09	7.56	6.47
	4	29.64	16.83	9.02	6.49
	5	23.15	15.18	9.86	2.89
	6	9.50	11.25	4.23	8.84
	7	28.76	15.05	9.82	2.89
	8	17.82	17.12	9.83	2.96
	9	0.00	16.31	8.07	3.92
	10	24.65	16.76	17.81	25.95
	11	18.99	16.62	8.63	7.24
	12	15.79	18.44	12.84	10.39
half-open	13	1.17	16.28	8.06	3.94
	14	2.91	16.24	7.98	3.79
	15	20.02	19.48	25.41	33.65
	16	8.72	16.54	23.63	31.33
	17	13.29	9.62	10.47	16.81
	18	41.17	20.48	27.26	34.44
	19	13.62	17.79	13.34	17.76
	20	29.87	14.74	16.21	19.04
open	21	35.56	14.87	14.62	8.68
	22	8.63	16.99	11.10	11.41
	23	28.40	11.07	13.57	18.91
	24	15.83	15.50	20.06	24.47
	25	10.51	18.28	26.83	35.70
	26	8.11	16.20	21.87	29.53
closed mean	1 – 12		15.70	9.44	7.12
half-open mean	13 - 20		16.40	16.55	20.10
open mean	21 - 26		15.49	18.01	21.45

3 NMR-spectra of new compounds

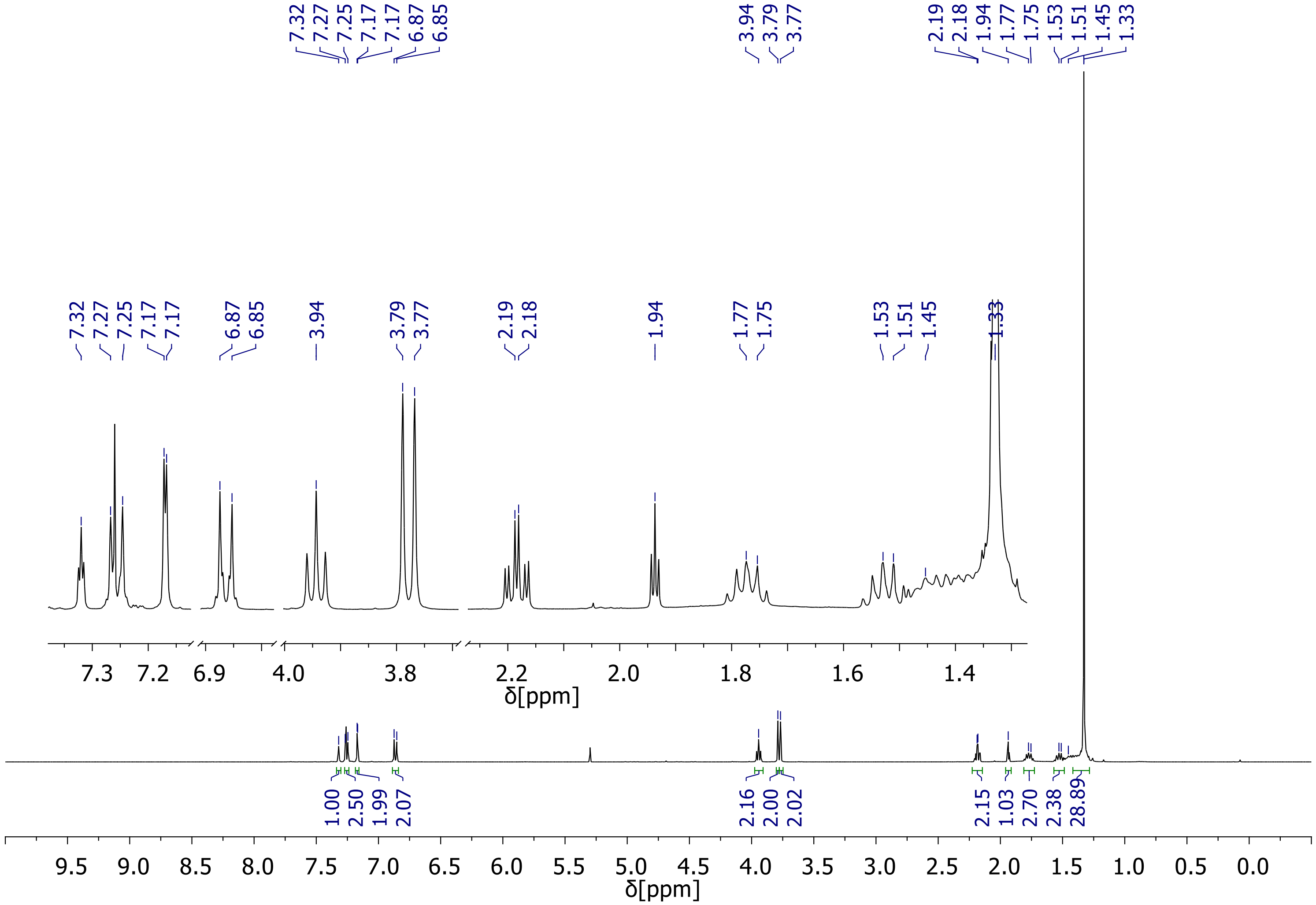

Figure S49: ^{1}H-NMR spectrum of dibenzylamine **4a** (400 MHz, $CDCl_3$, 298K).

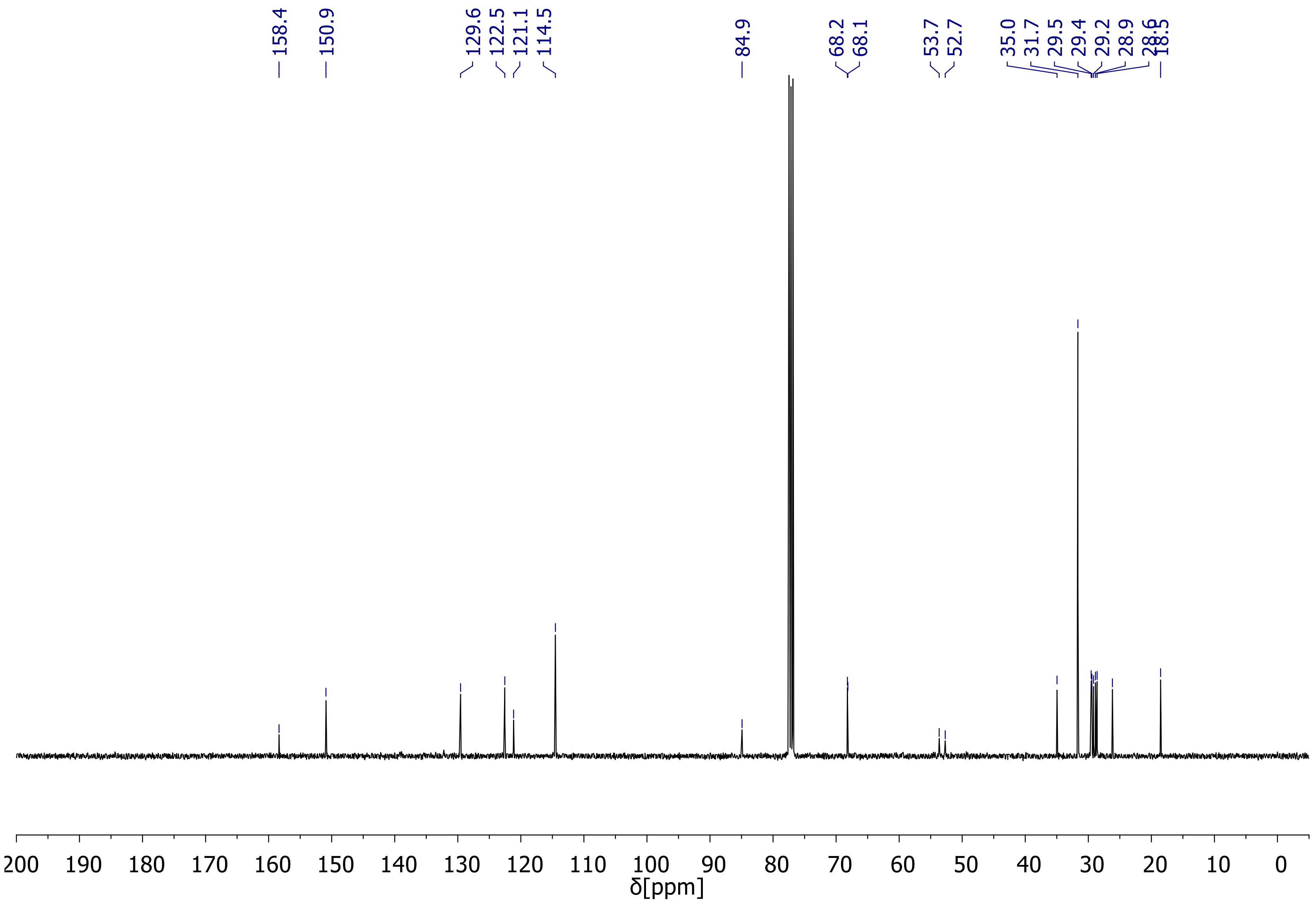

Figure S50: ^{13}C-NMR spectrum of dibenzylamine **4a** (101 MHz, 298 K, $CDCl_3$).

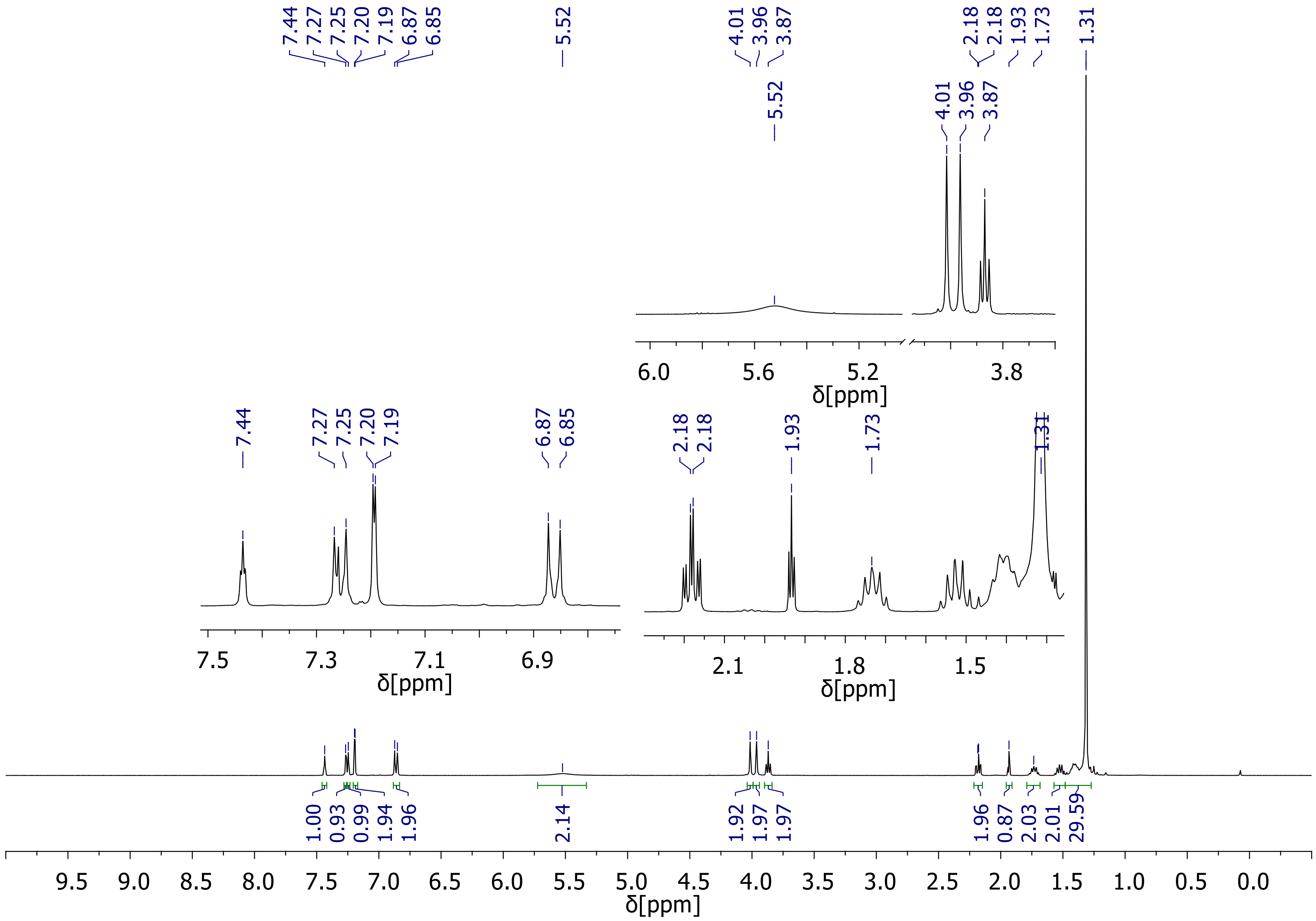

Figure S51: ^{1}H-NMR spectrum of dibenzylammonium **4a-HPF₆** (400 MHz, $CDCl_3$, 298K).

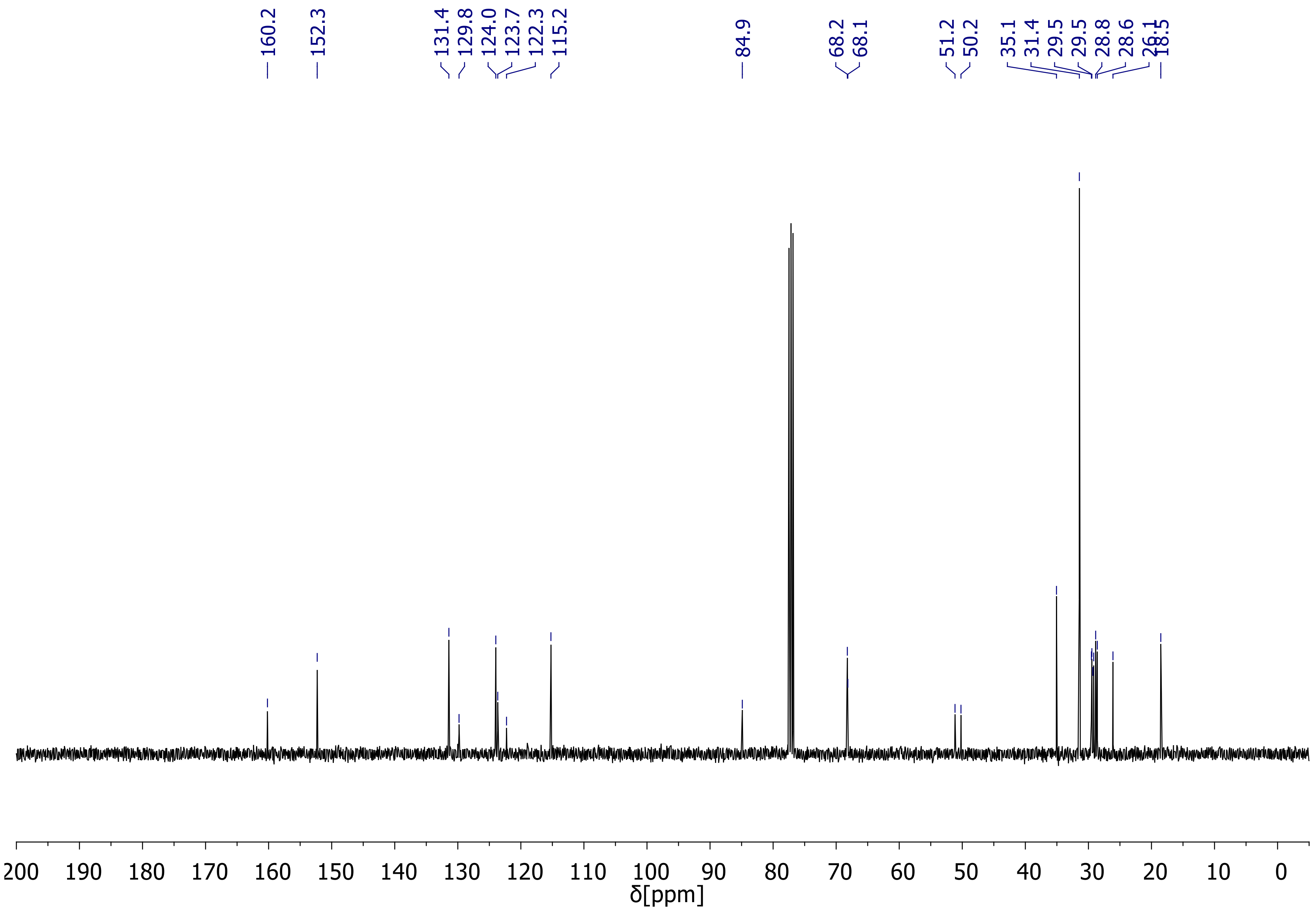

Figure S 52: ^{13}C-NMR spectrum (101 MHz, 298 K, $CDCl_3$) of dibenzylammonium **4a-HPF₆**.

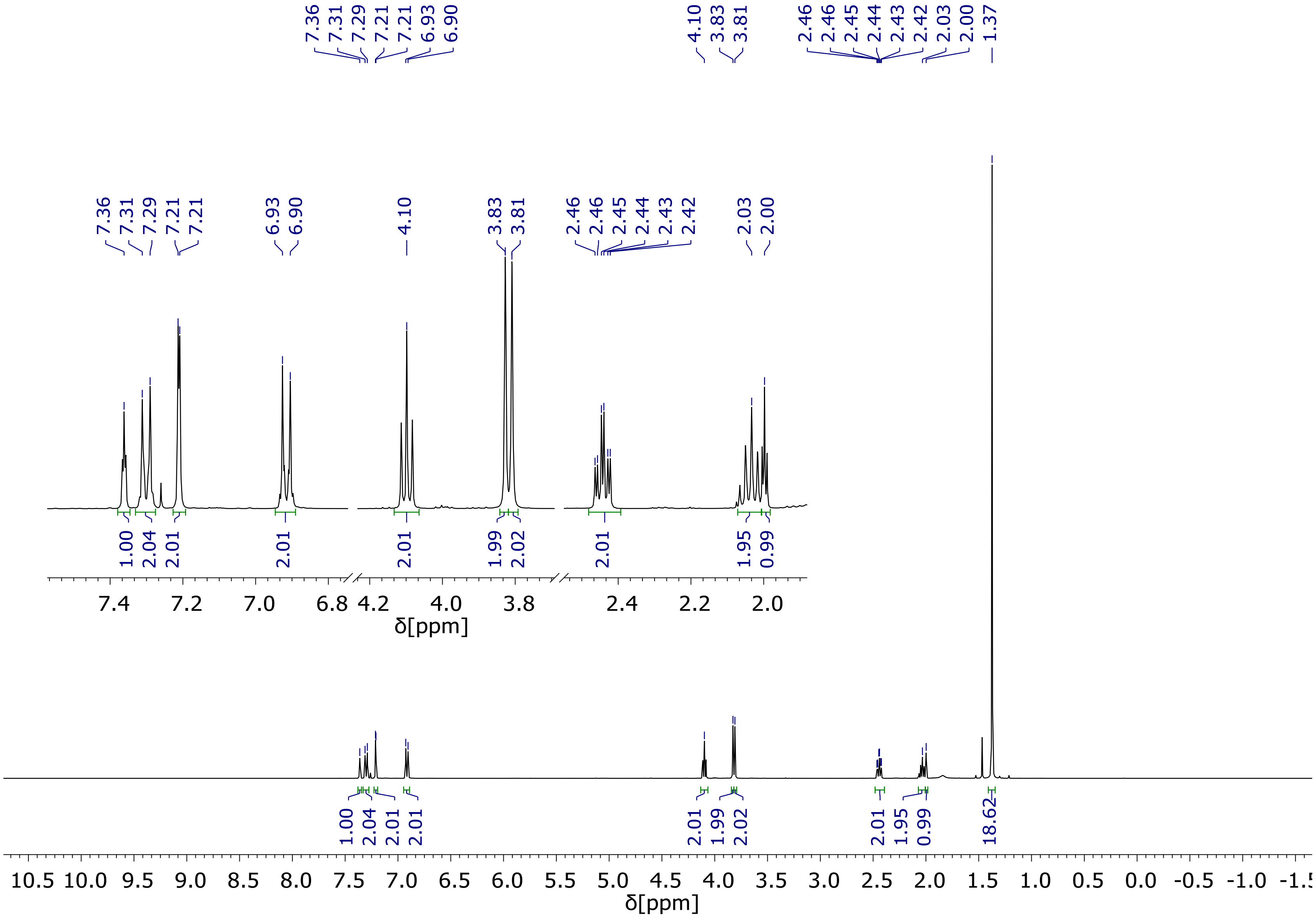

Figure S53: ^{1}H-NMR spectrum of dibenzylamine **4b** (400 MHz, $CDCl_3$, 298K).

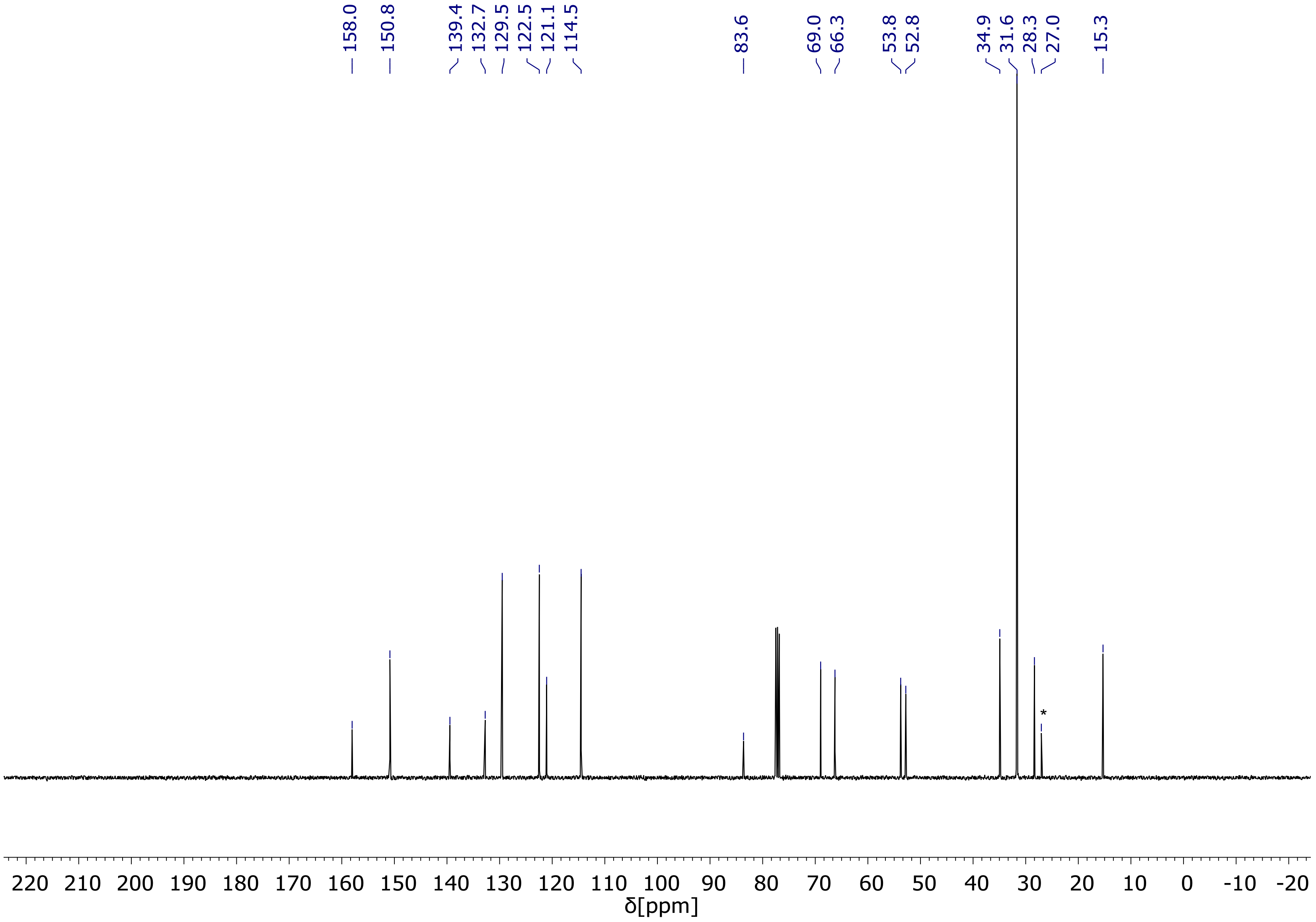

Figure S54: : ^{13}C-NMR spectrum (101 MHz, 298 K, $CDCl_3$) of dibenzylamine **4b**; * = residual cyclohexane.

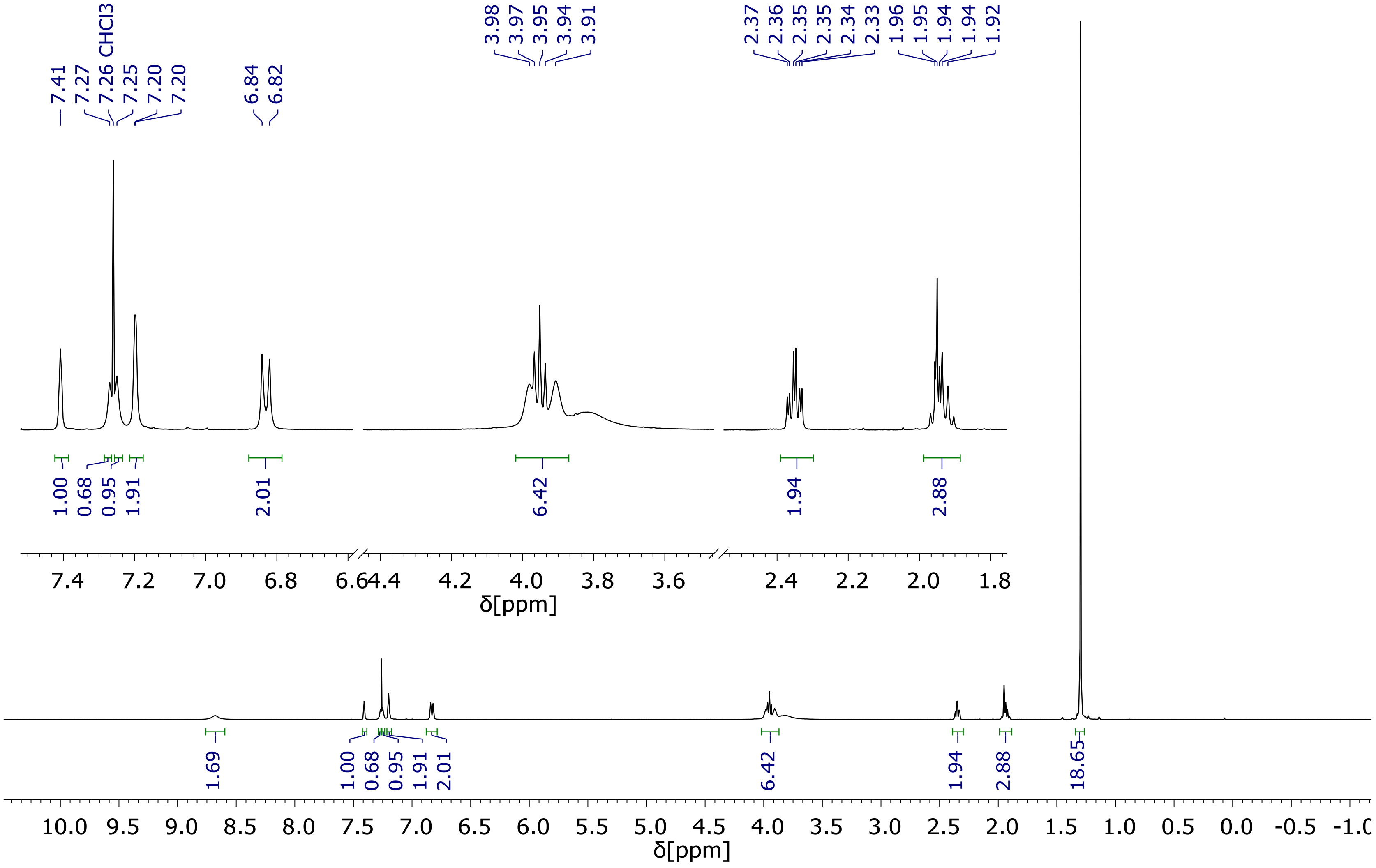

Figure S55: ^{1}H-NMR spectrum of dibenzylammonium **4b-HPF$_6$** (400 MHz, $CDCl_3$, 298K).

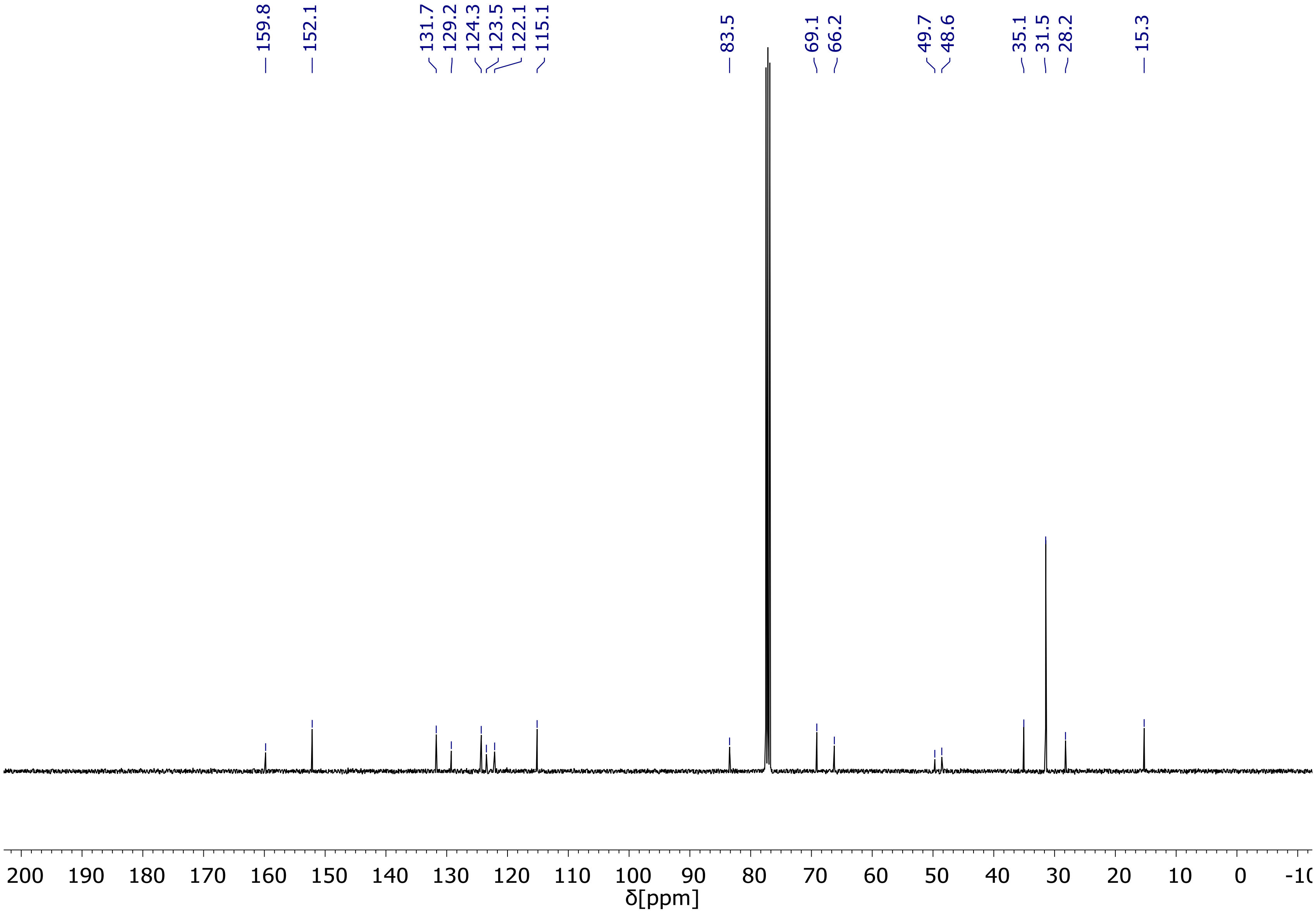

Figure S56: ^{13}C-NMR spectrum (101 MHz, 298 K, $CDCl_3$) of dibenzylammonium **4b-HPF₆**.

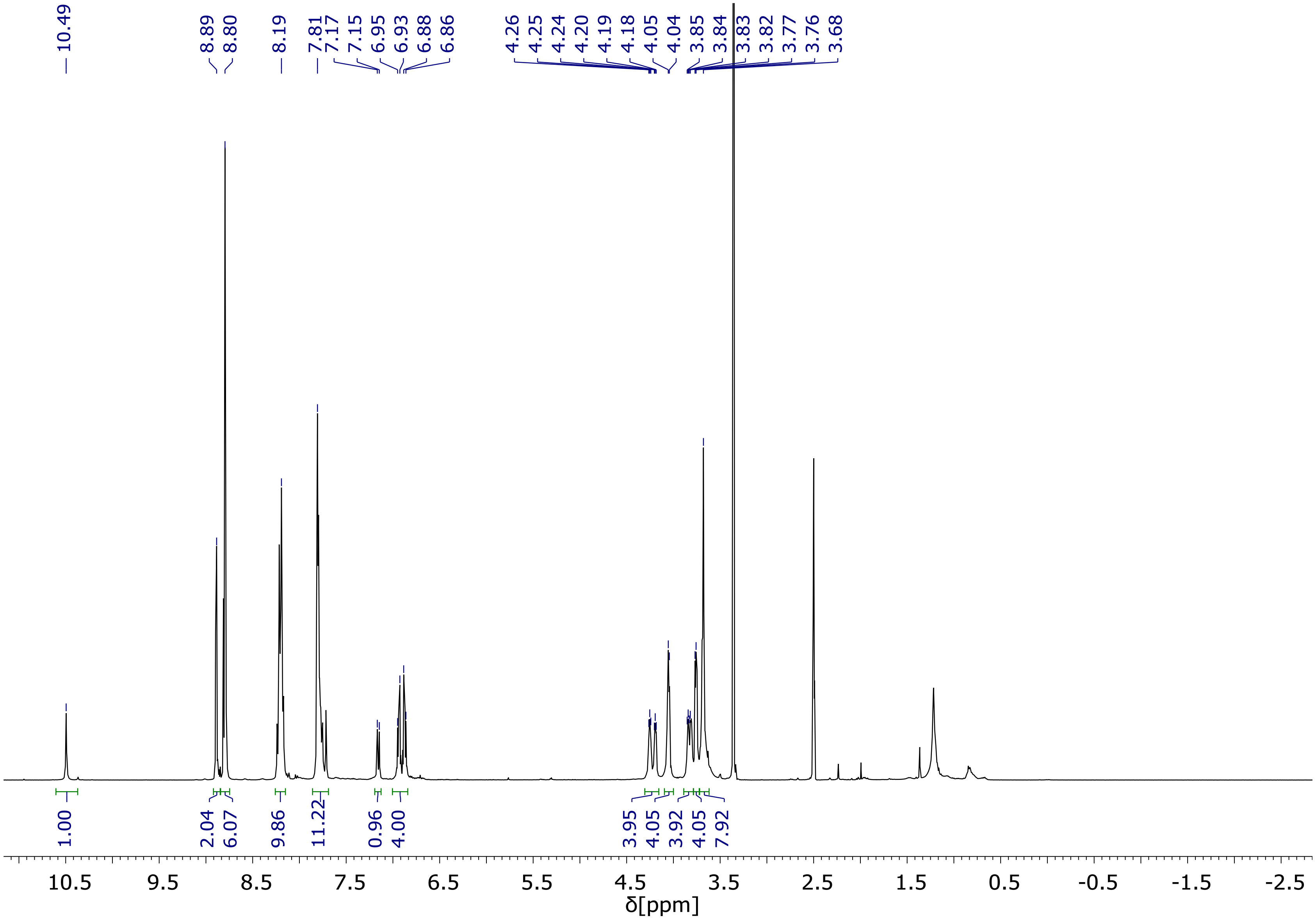

Figure S57: ^{1}H-NMR (400 MHz, DMSO-d_6, 298 K) of porphyrin decorated crown-ether **5**.

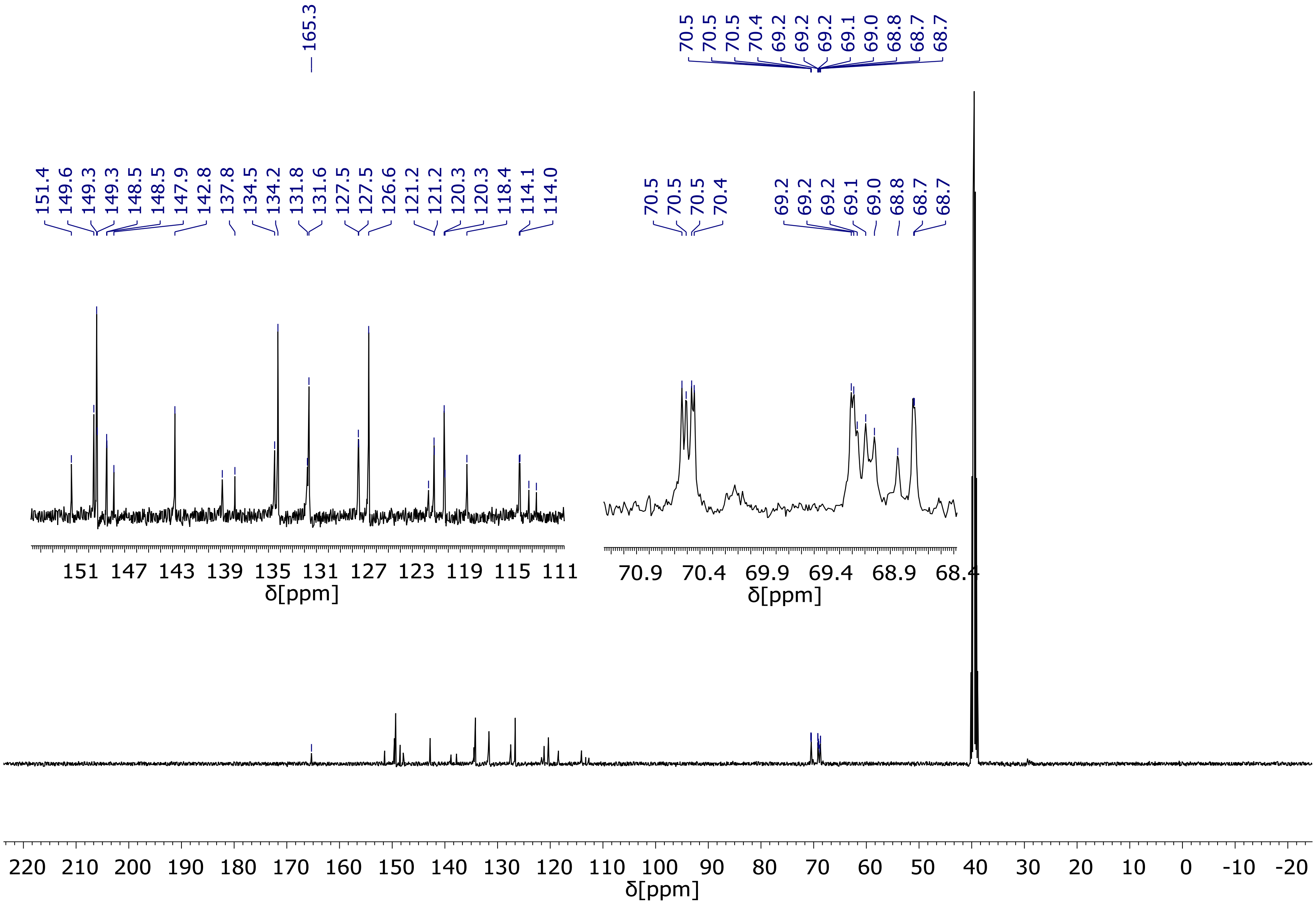

Figure S58: ^{13}C-NMR (101 MHz, DMSO-d_6, 298 K) of porphyrin decorated crown-ether **5**.

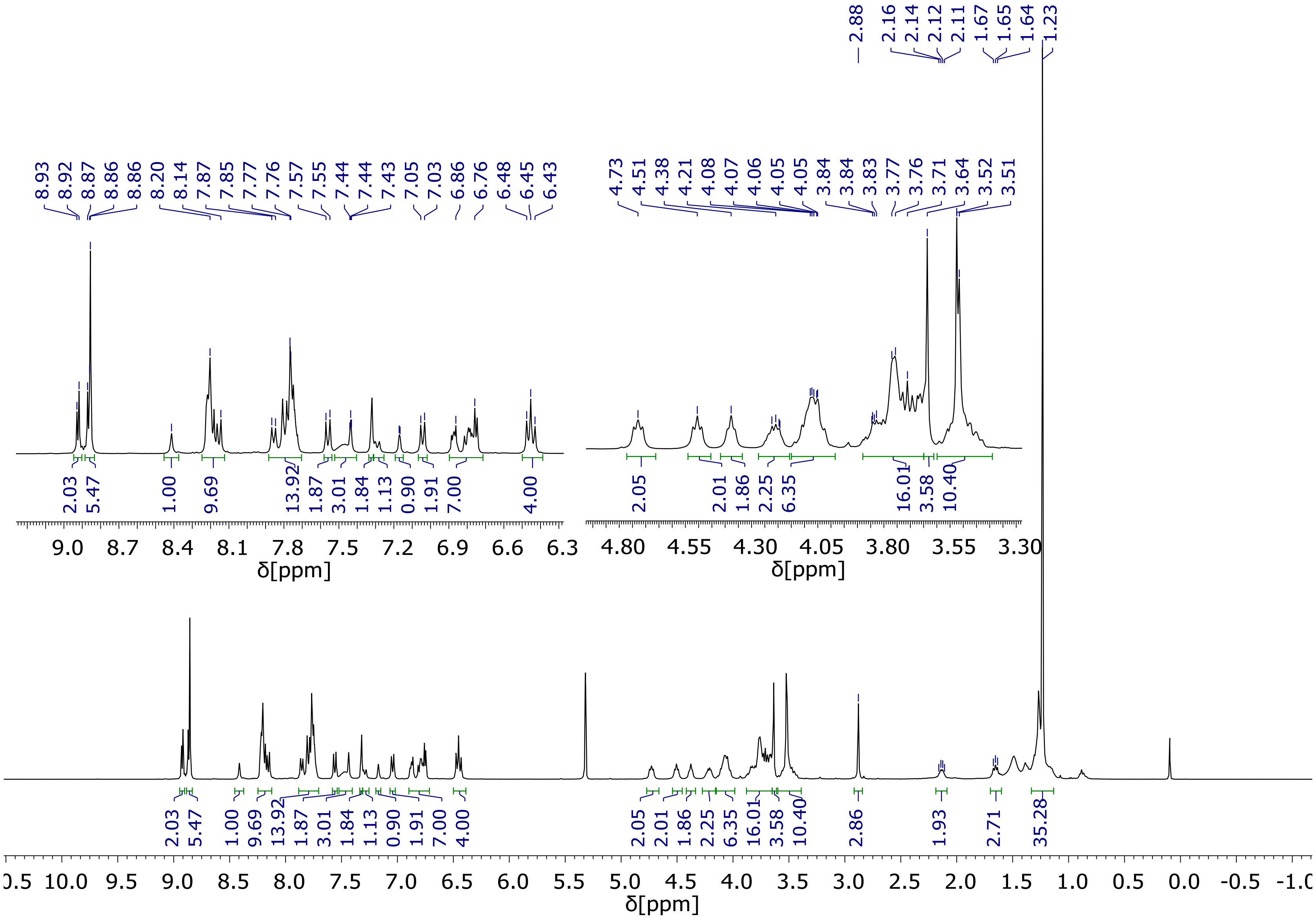

Figure S59: ^{1}H-NMR (400 MHz, CD_2Cl_2, 298 K) of rotaxane **1a-H(PF$_6$)$_2$**).

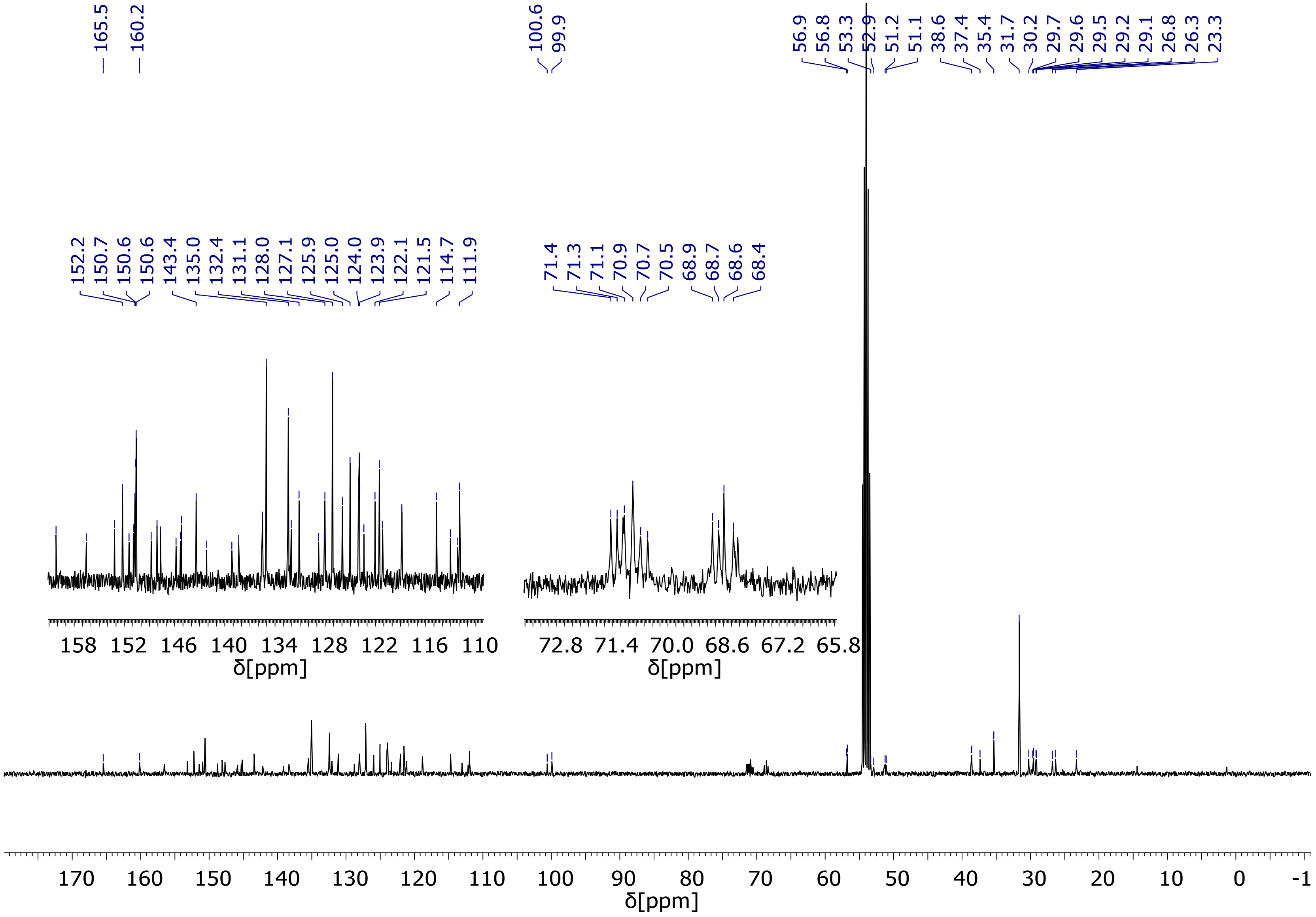

Figure S60: ^{13}C-NMR (101 MHz, 298 K, CD_2Cl_2) of rotaxane **1a-H(PF$_6$)$_2$**).

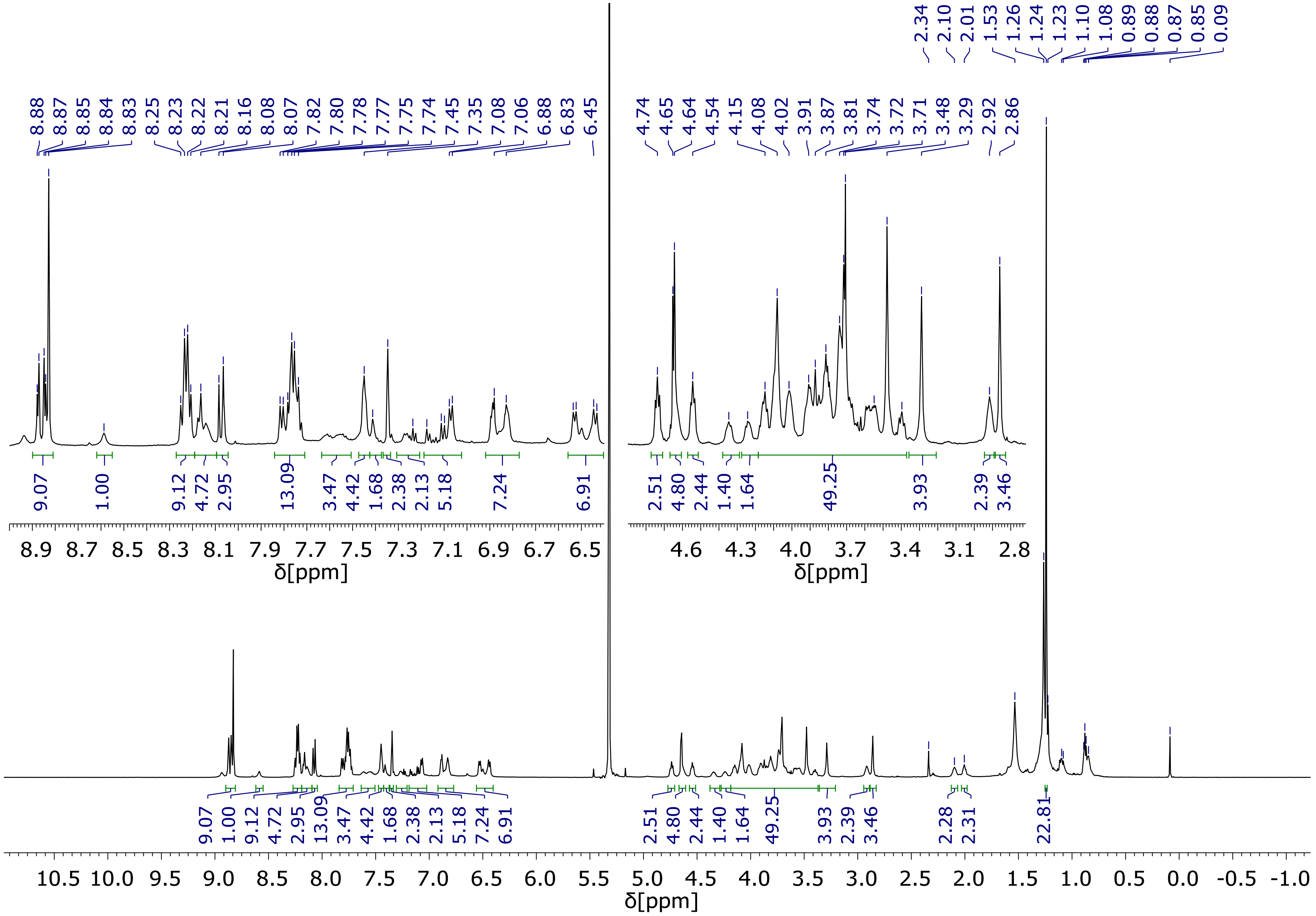

Figure S61: ^{1}H-NMR (600 MHz, CD_2Cl_2, 298 K) of shorter rotaxane **1b-H(PF$_6$)$_2$**).

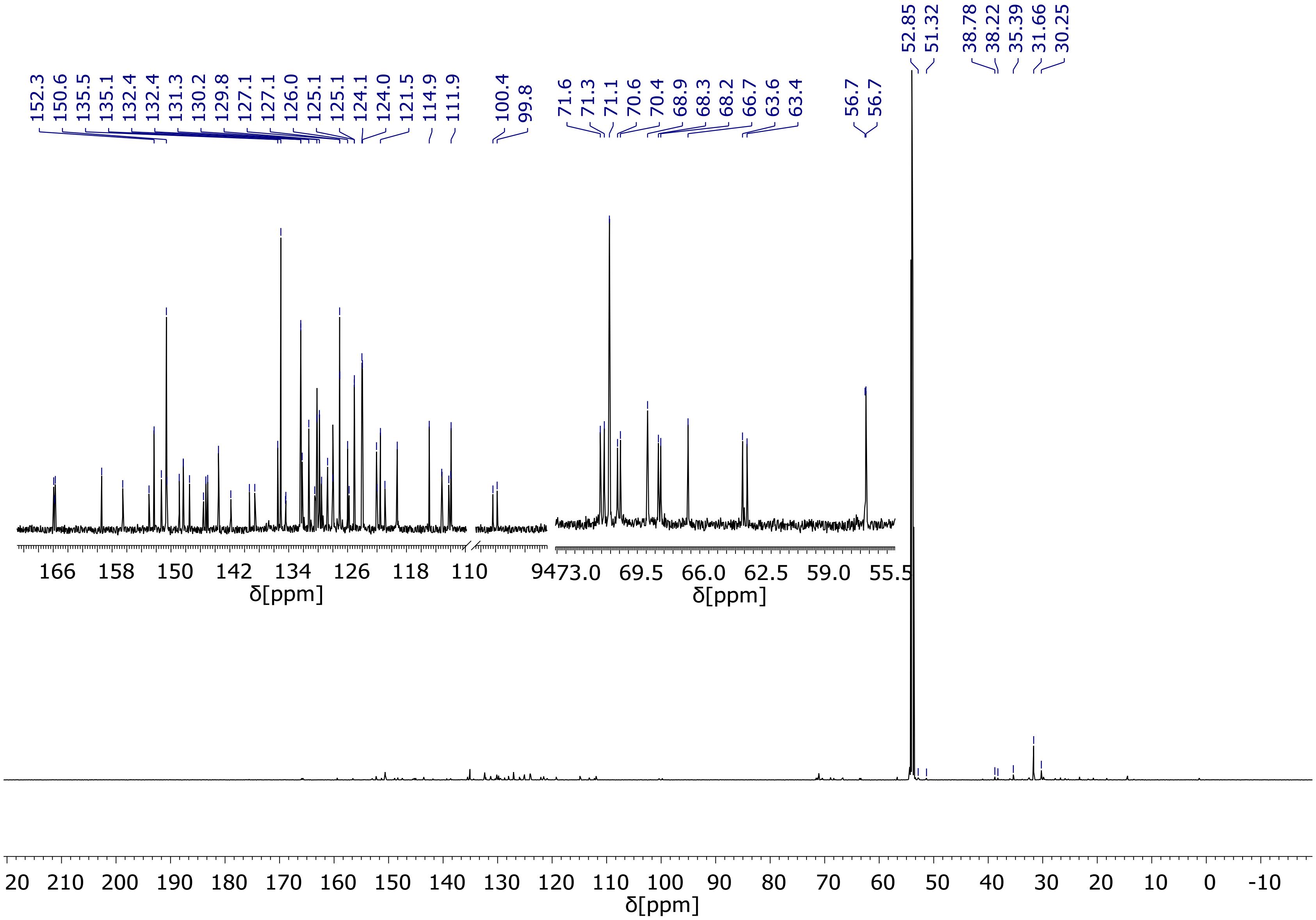

Figure S62: ^{13}C-NMR (151 MHz, 298 K, CD_2Cl_2) of shorter rotaxane **1b-H(PF$_6$)$_2$)**.

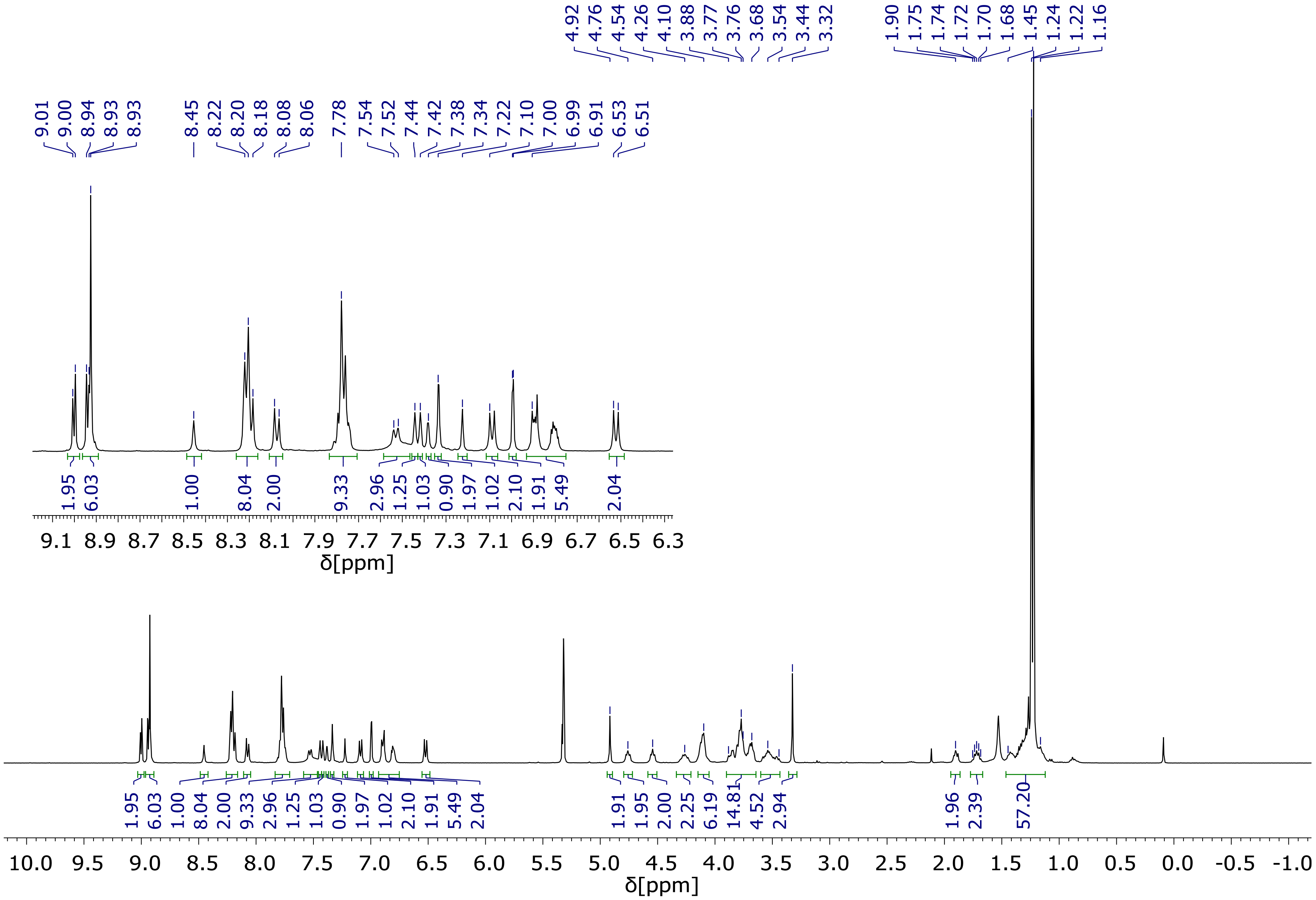

Figure S63: ^{1}H-NMR (400 MHz, CD_2Cl_2, 298 K) of dummy rotaxane **2-H(PF$_6$)$_2$**.

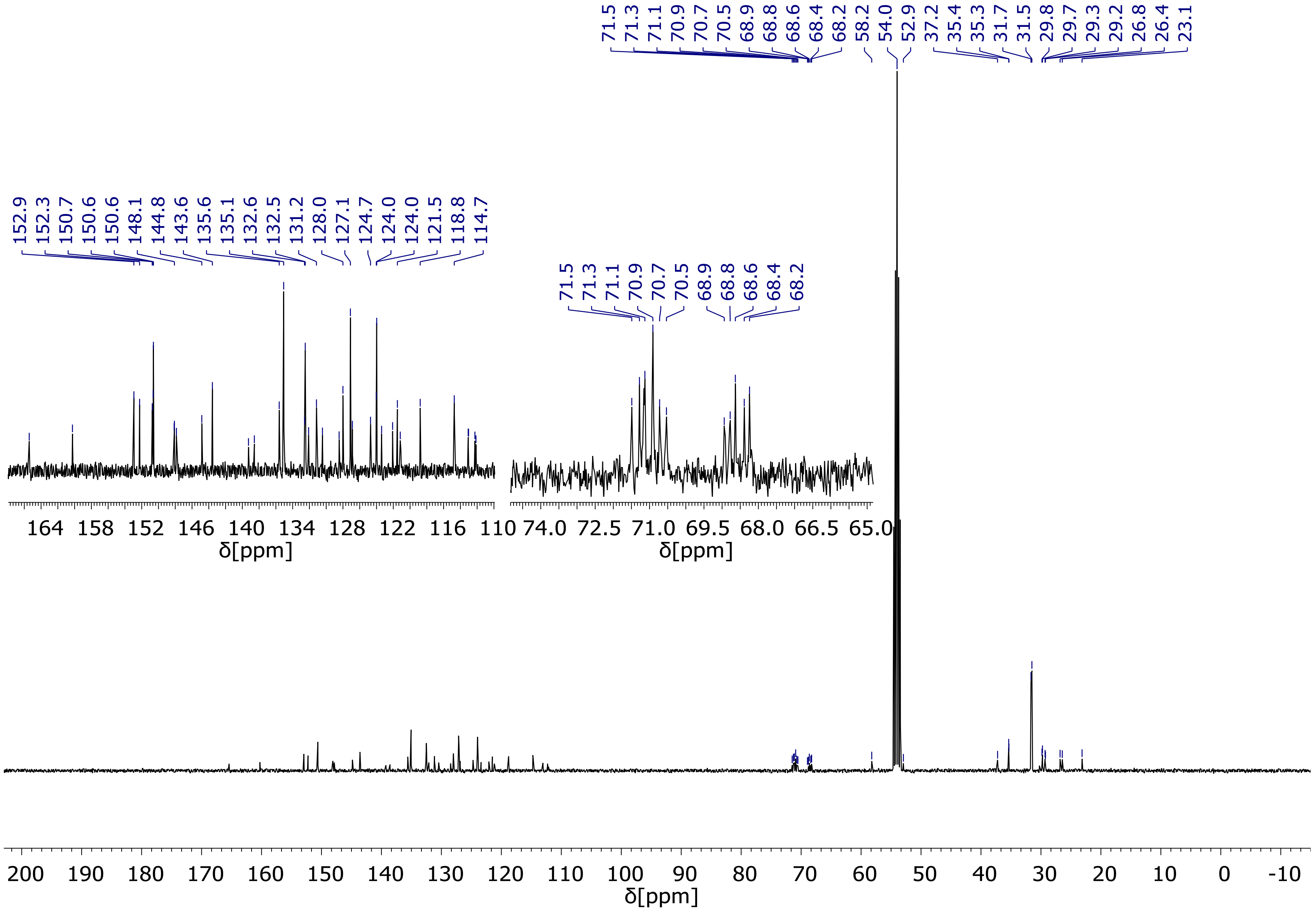

Figure S64: ^{13}C-NMR (101MHz, CD_2Cl_2, 298 K) of dummy rotaxane **2-H(PF$_6$)$_2$**.

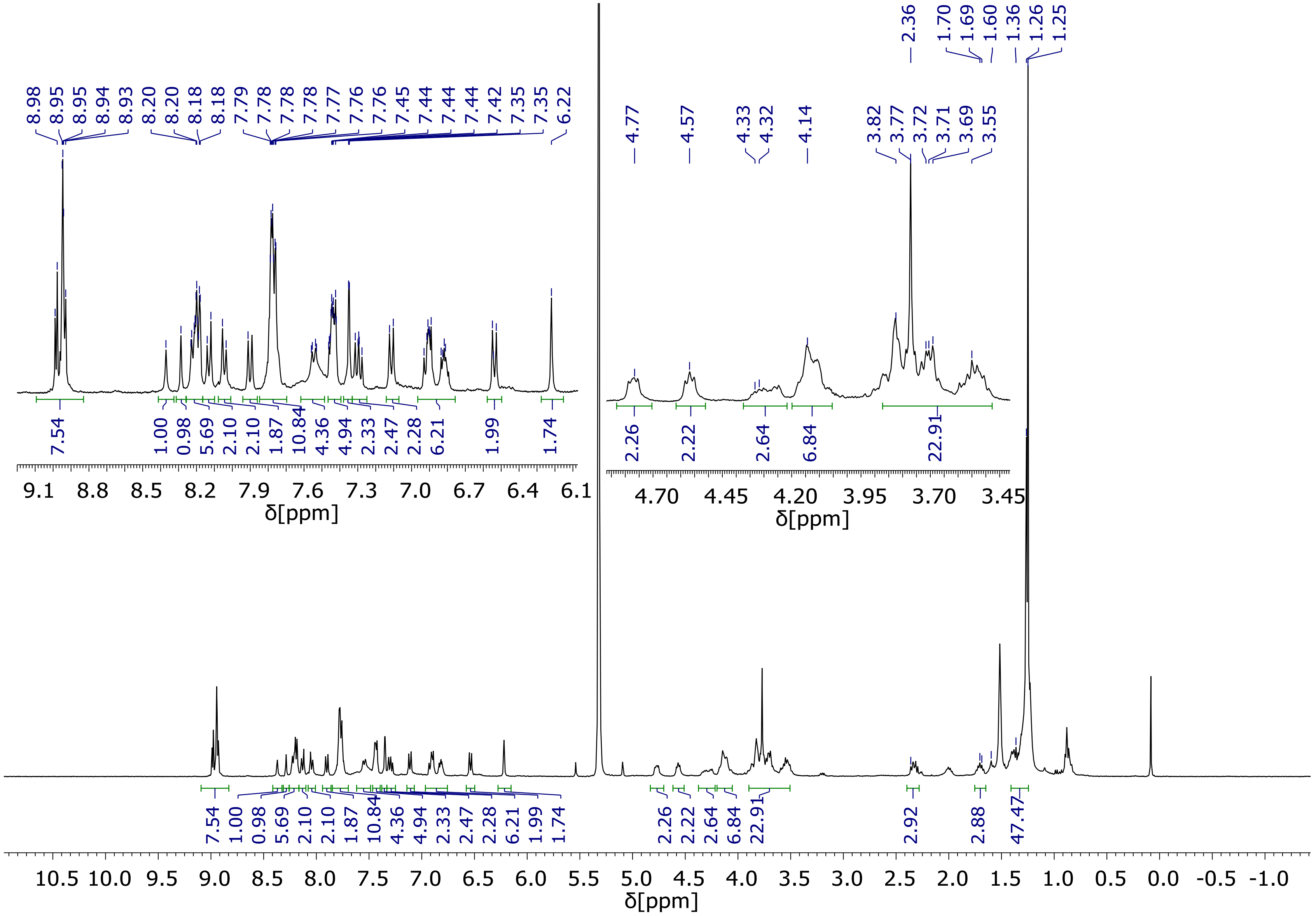

Figure S65: ^{1}H-NMR (400 MHz, CD_2Cl_2, 298 K) of anthracene rotaxane **3-H(PF$_6$)$_2$**.

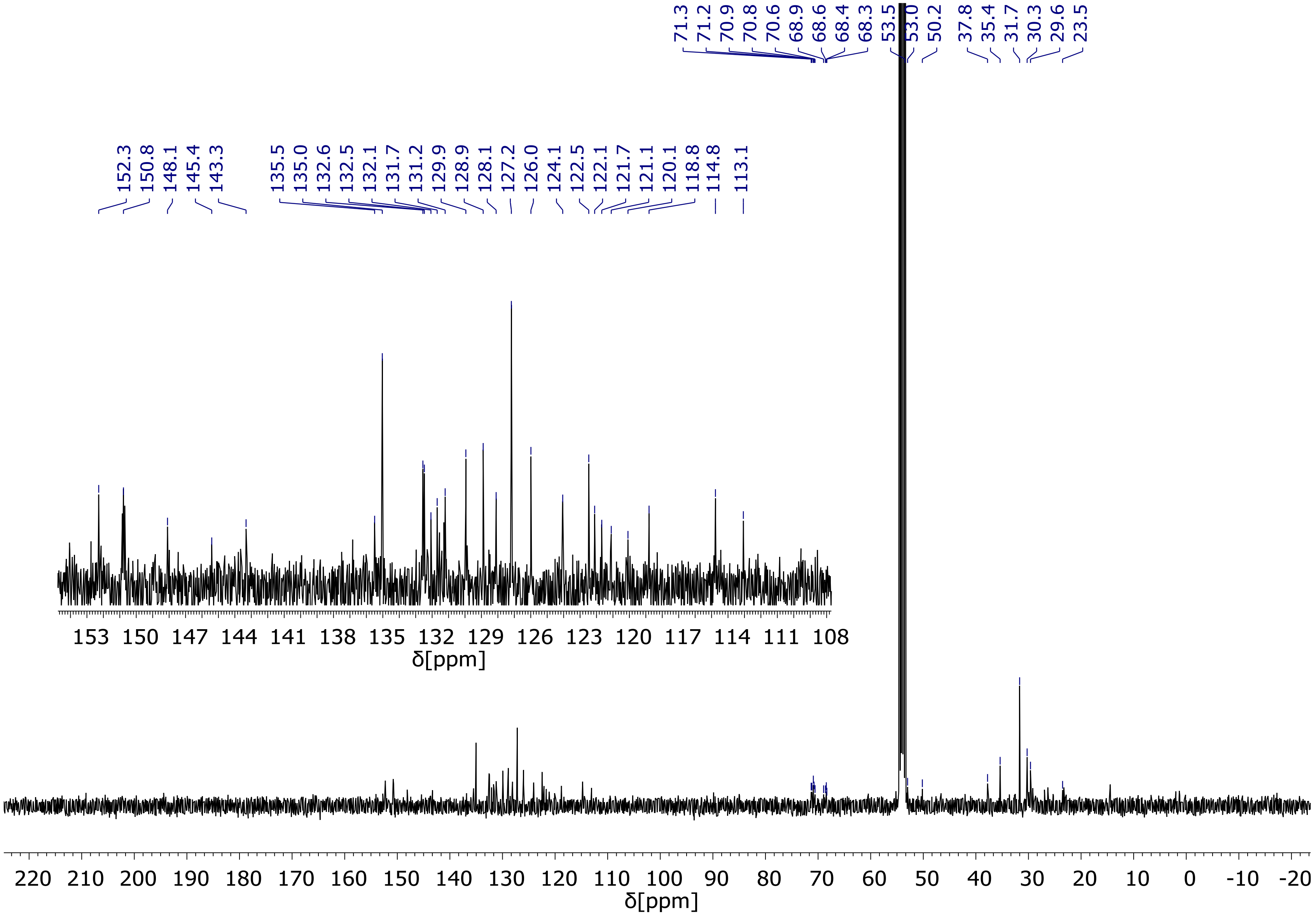

Figure S66: ^{13}C-NMR (101MHz, CD_2Cl_2, 298 K) of anthracene rotaxane **3-H(PF$_6$)$_2$**.

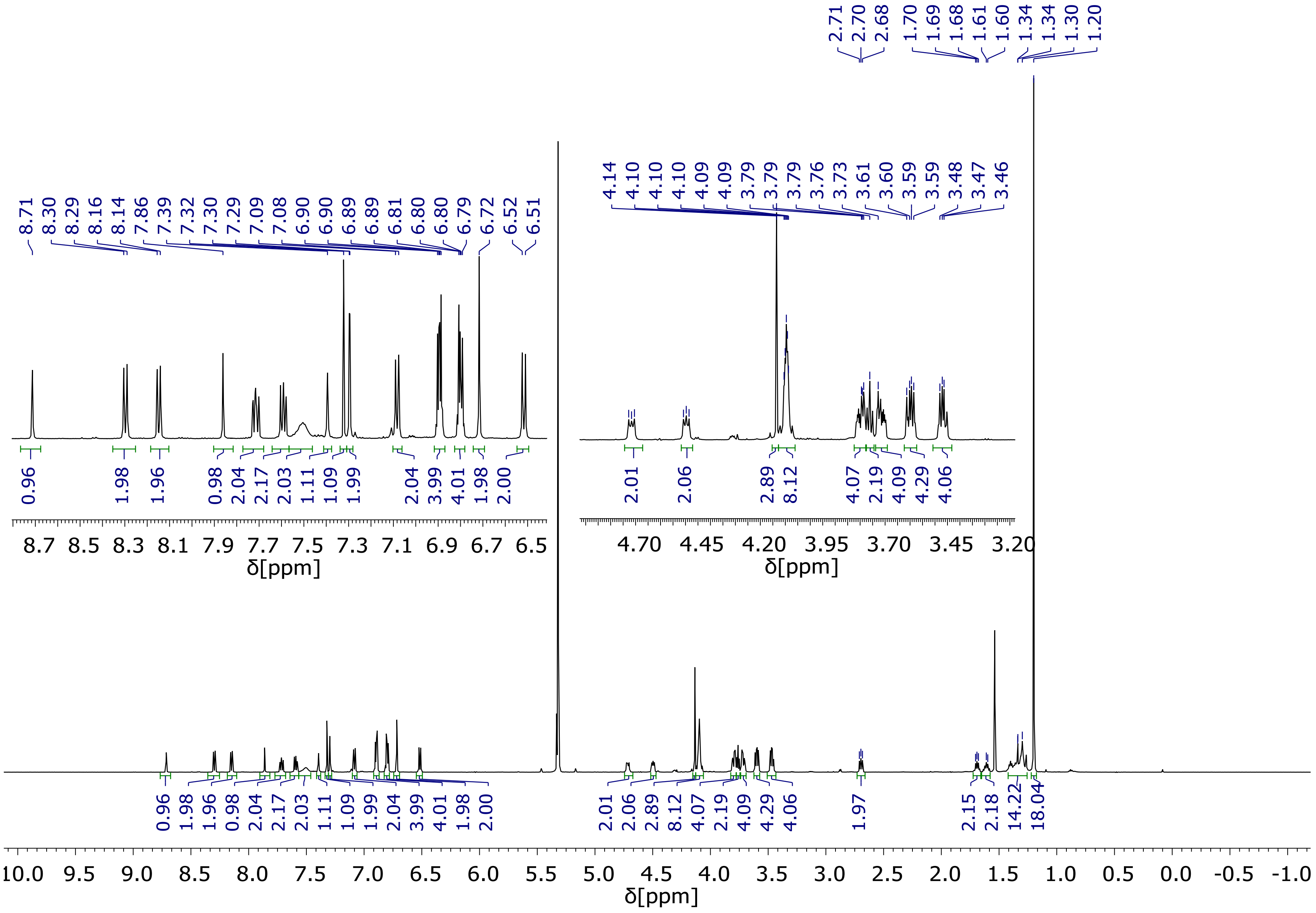

Figure S67:^{1}H-NMR (600 MHz, CD_2Cl_2, 298 K,) of dummy anthracene-rotaxane **S20-H(PF_6)$_2$**

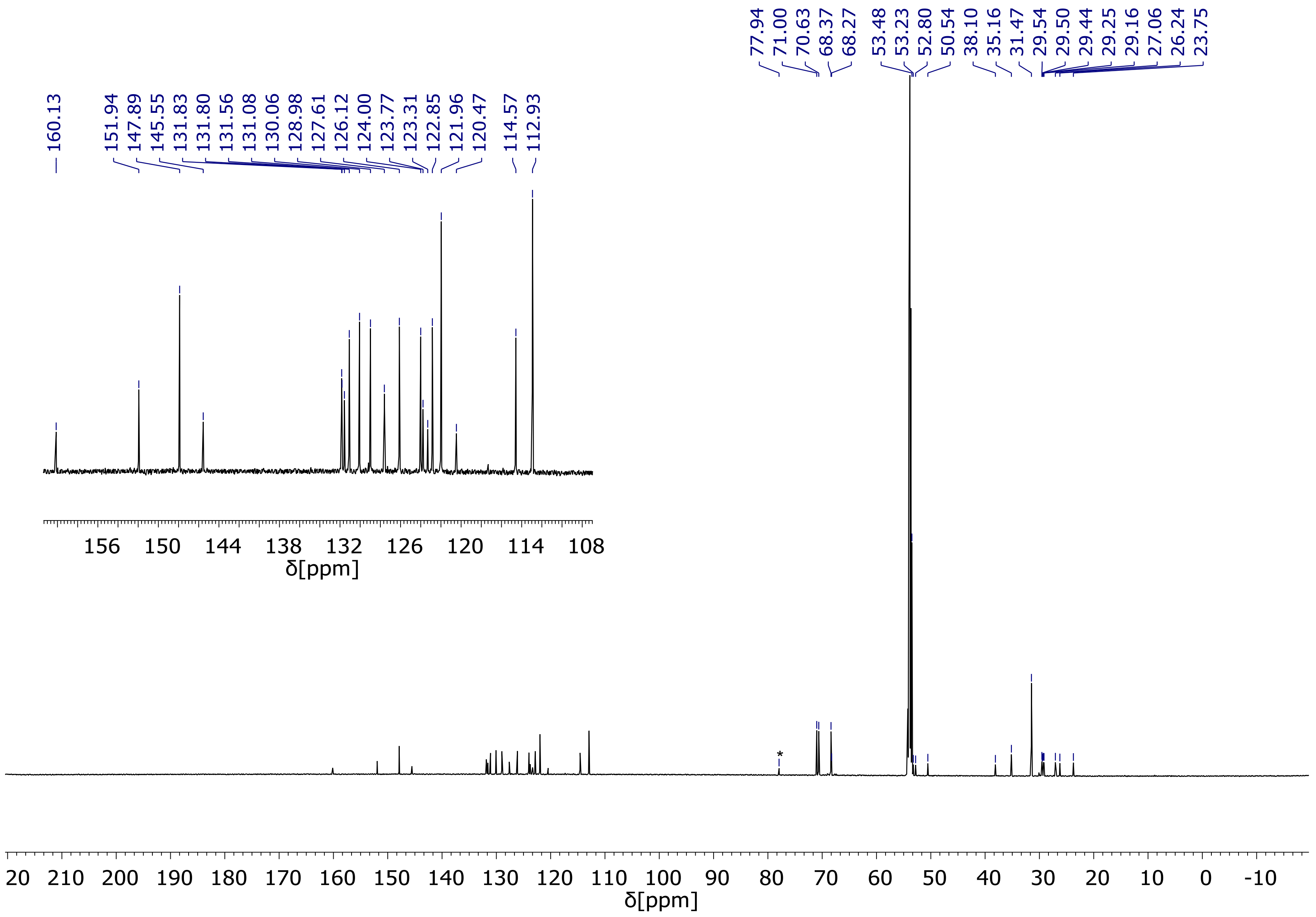

Figure S68: ^{13}C-NMR (151 MHz, CD_2Cl_2, 298 K) of dummy anthracene-rotaxane S**20-H(PF$_6$)$_2$**. * = residual chloroform

4 Supplementary References

1. Adler, A. D., Longo, F. R., Finarelli, J. D., Goldmacher, J., Assour, J. & Korsakoff, L. A simplified synthesis for meso-tetraphenylporphine. *J. Org. Chem.* **32**, 476-476 (1967).
2. Asano, K. & Matsubara, S. Amphiphilic Organocatalyst for Schotten-Baumann-Type Tosylation of Alcohols under Organic Solvent Free Condition. *Org. Lett.* **11**, 1757-1759 (2009).
3. Edwards, G. L., Muldoon, C. A. & Sinclair, D. J. Cyclic enol ether synthesis via arenesulfonyl iodide additions to alkynols. *Tetrahedron* **52**, 7779-7788 (1996).
4. Ranjan, N., *et al.* Selective inhibition of bacterial topoisomerase I by alkynyl-bisbenzimidazoles. *Med. Chem. Commun.* **5**, 816-825 (2014).
5. Ajay, A., *et al.* Diversity Oriented Synthesis of Pyran Based Polyfunctional Stereogenic Macrocyles and Their Conformational Studies. *Org. Lett.* **14**, 4306-4309 (2012).
6. Weiss, H., *et al.* Curcuminoid–BF2 complexes: Synthesis, fluorescence and optimization of BF2 group cleavage. *Beilstein J. Org. Chem.* **13**, 2264-2272 (2017).
7. Verheyde, B. & Dehaen, W. Synthesis of Dendrimers Containing 1,3,4-Oxadiazoles. *J. Org. Chem.* **66**, 4062-4064 (2001).
8. Maillard, M., Baldwin, E.T., Beck, J.T., Hughes, R., John, V., Pulley S.R., Tenbrink, R. Patent WO2004024081 (2004).
9. Bhatt, R. K., Sharma, S. & Nath, M. La(OTf)3-catalyzed one-pot synthesis of meso-substituted porphyrinic thiazolidinones. *Monatsh. Chemie* **143**, 309-316 (2011).
10. Kruper, W. J., Chamberlin, T. A. & Kochanny, M. Regiospecific aryl nitration of meso-substituted tetraarylporphyrins: a simple route to bifunctional porphyrins. *J. Org. Chem.* **54**, 2753-2756 (1989).
11. Konev, A. S., Khlebnikov, A. F., Levin, O. V., Lukyanov, D. A. & Zorin, I. M. Photocurrent in Multilayered Assemblies of Porphyrin–Fullerene Covalent Dyads: Evidence for Channels for Charge Transport. *ChemSusChem* **9**, 676-686 (2016).
12. Liu, D., *et al.* Supramolecular Organogel Based on Crown Ether and Secondary Ammoniumion Functionalized Glycidyl Triazole Polymers. *Macromolecules* **46**, 4617-4625 (2013).
13. Feng, D.-J., Li, X.-Q., Wang, X.-Z., Jiang, X.-K. & Li, Z.-T. Highly stable pseudo[2]rotaxanes co-driven by crown ether–ammonium and donor–acceptor interactions. *Tetrahedron* **60**, 6137-6144 (2004).
14. Chevalier, A., Mercier, C., Saurel, L., Orenga, S., Renard, P.-Y. & Romieu, A. The first latent green fluorophores for the detection of azoreductase activity in bacterial cultures. *Chem. Commun.* **49**, 8815 (2013).
15. Ghosh, A., Paul, I., Adlung, M., Wickleder, C. & Schmittel, M. Oscillating Emission of [2]Rotaxane Driven by Chemical Fuel. *Org. Lett.* **20**, 1046-1049 (2018).
16. Mettry, M., Moehlig, M. P., Gill, A. D. & Hooley, R. J. Alkane oxidation catalysed by a self-folded multi-iron complex. *Supramol. Chem.* **29**, 120-128 (2016).
17. Curcio, M., *et al.* Chemically Induced Mismatch of Rings and Stations in [3]Rotaxanes. *J. Am. Chem. Soc.* **143**, 8046-8055 (2021).

18. Chevalier, A., Renard, P.-Y. & Romieu, A. Straightforward synthesis of bioconjugatable azo dyes. Part 2: Black Hole Quencher-2 (BHQ-2) and BlackBerry Quencher 650 (BBQ-650) scaffolds. *Tetrahedron Lett.* **55**, 6764-6768 (2014).

19. Ormond, A. B. & Freeman, H. S. Effects of substituents on the photophysical properties of symmetrical porphyrins. *Dyes Pigment.* **96**, 440-448 (2013).

20. Hildebrandt, N., How to Apply FRET: From Experimental Design to Data Analysis, in: Medintz, I., Hildebrandt, N. (Eds.), FRET – Förster Resonance Energy Transfer, Wiley-VCH, Weinheim, 2023, pp. 105-163.

21. Srinivas, G. & Bagchi, B. Effect of Orientational Motion of Mobile Chromophores on the Dynamics of Förster Energy Transfer in Polymers. *J. Phys. Chem. B* **105**, 9370-9374 (2001).

22. Sindbert, S., *et al.* Accurate Distance Determination of Nucleic Acids via Förster Resonance Energy Transfer: Implications of Dye Linker Length and Rigidity. *J. Am. Chem. Soc.* **133**, 2463-2480 (2011).

23. Schmidt, R. & Afshari, E. Comment on "Effect of solvent on the phosphorescence rate constant of singlet molecular oxygen". J. *Phys. Chem.* **94,** 4377-4378 (1990).

24. Grimme, S. Exploration of Chemical Compound, Conformer, and Reaction Space with Meta-Dynamics Simulations Based on Tight-Binding Quantum Chemical Calculations. *J. Chem. Theory Comput.* 1**5,** 2847-2862 (2019).

25. Pracht, P., Bohle, F. & Grimme, S. Automated exploration of the low-energy chemical space with fast quantum chemical methods. *Phys. Chem. Chem. Phys.* **22,** 7169-7192 (2020).

26. Bannwarth, C., *et al.* Extended tight-binding quantum chemistry methods. *WIREs Comput. Mol. Sci.* **11,** e1493 (2020).

27. Grimme, S., Bannwarth, C. & Shushkov, P. A Robust and Accurate Tight-Binding Quantum Chemical Method for Structures, Vibrational Frequencies, and Noncovalent Interactions of Large Molecular Systems Parametrized for All spd-Block Elements (Z = 1–86). *J. Chem. Theory Comput.* **13,** 1989-2009 (2017).

28. Bannwarth, C., Ehlert, S. & Grimme, S. GFN2-xTB - An Accurate and Broadly Parametrized Self-Consistent Tight-Binding Quantum Chemical Method with Multipole Electrostatics and Density-Dependent Dispersion Contributions. *J. Chem. Theory Comput.* **15,** 1652-1671 (2019).

29. Spicher, S. & Grimme, S. Robust Atomistic Modeling of Materials, Organometallic, and Biochemical Systems. *Angew. Chem. Int. Ed.* **59,** 15665-15673 (2020).

30. Ehlert, S., Stahn, M., Spicher, S. & Grimme, S. Robust and Efficient Implicit Solvation Model for Fast Semiempirical Methods. *J. Chem. Theory Comput.* **17,** 4250-4261 (2021).

31. Pettersen, E. F., *et al.* UCSF Chimera - A visualization system for exploratory research and analysis. *J. Comput. Chem.* **25,** 1605-1612 (2004).

32. Bursch, M., Mewes, J. M., Hansen, A. & Grimme, S. Best-Practice DFT Protocols for Basic Molecular Computational Chemistry. *Angew. Chem. Int. Ed.* 6**1,** e202205735 (2022).

33. https://github.com/grimme-lab/numsa

34. Spicher, S. & Grimme, S. Single-Point Hessian Calculations for Improved Vibrational Frequencies and Rigid-Rotor-Harmonic-Oscillator Thermodynamics. *J. Chem. Theory Comput.* **17,** 1701-1714 (2021).

35. https://github.com/grimme-lab/xtb

36. Balasubramani, S. G., *et al.* TURBOMOLE: Modular program suite for ab initio quantum-chemical and condensed-matter simulations. *J. Chem. Phys.* **152,** 184107 (2020).

37. https://www.turbomole.org

38. Grimme, S., Brandenburg, J. G., Bannwarth, C. & Hansen, A. Consistent structures and interactions by density functional theory with small atomic orbital basis sets. *J. Chem. Phys.* **143,** 054107 (2015).

39. Klamt, A. The COSMO and COSMO-RS solvation models. W*IREs Comput. Mol. Sci.* **1,** 699-709 (2011).
