# Supplementary material for: Molecular folding governs switchable singlet oxygen photoproduction in porphyrin-decorated bistable rotaxanes
Source: Commun Chem. 2024 Aug 7;7:171. doi: 10.1038/s42004-024-01247-7 (PMC11306352; doi:10.1038/s42004-024-01247-7)
Supplement: Supplementary file 3 — Description of Additional Supplementary Files [file 42004_2024_1247_MOESM3_ESM.pdf]

Description of Additional Supplementary Files

File name: Supplementary Data 1

Description: xyz-483 files of the calculated conformers of the rotaxanes.

File name: Supplementary Data 2

Description: Numerical 484 source data for determination of the singlet oxygen quantum yields.
